# Multi-environment evaluation identifies high-yielding and stable winged bean (*Psophocarpus tetragonolobus* (L.) DC.) accessions for sub-Saharan Africa

**DOI:** 10.3389/fpls.2026.1916275

**Published:** 2026-09-30

**Authors:** Ufuoma L. Akpojotor, Jelili T. Opabode, Olaniyi A. Oyatomi, Rajneesh Paliwal, Michael T. Abberton

**Affiliations:** 1 Genetic Resources Centre, International Institute of Tropical Agriculture, Ibadan, Oyo State, Nigeria; 2 Department of Crop Production and Protection, Obafemi Awolowo University, Ile-Ife, Osun State, Nigeria

**Keywords:** AMMI, genetic variability, GGE biplot, Psophocarpus tetragonolobus, seed yield stability

## Abstract

The improvement of winged bean offers great importance for food and nutritional security in sub-Saharan Africa under climate change, yet limited information exists on the genetic variability and seed yield stability of available germplasm under multi-environment conditions. Therefore, the identification of high-yielding and stable accessions with broad adaptation has become a major objective in winged bean improvement programs. This study evaluated 110 winged bean accessions for 21 agronomic and yield-related traits across six environments in Nigeria during the 2022 and 2023 cropping seasons using an alpha lattice design in three replicates. Data were subjected to combined analysis of variance, estimation of genetic variability parameters, phenotypic and genotypic correlation, additive main effects and multiplicative interaction (AMMI), and accession × environment interaction (GGE) biplot analyses. Highly significant differences (*p* < 0.001) were observed among accessions and environments for all traits, and the accession × environment interaction was significant (*p* < 0.05) for all traits except hundred seed weight. Total seed weight exhibited moderate genotypic and phenotypic coefficients of variation, while total pod weight, seed weight per plant and number of seeds per plant combined low genotypic coefficients of variation with moderate broad-sense heritability and moderate genetic advance, indicating scope for improvement through selection. Highly significant positive correlations were observed among pod and seed yield-related traits. Stability analysis of the 20 best- and 10 poorest-performing accessions partitioned 75.28% of the total variation to the environment, 15.94% to the accession × environment interaction and 8.78% to accessions, confirming that the environment strongly influenced seed yield expression in winged bean. Both AMMI and GGE biplot analyses consistently identified TPt-270 (G70) as the highest-yielding accession, closely followed by TPt-198 (G31), while TPt-187 (G29) was identified as the accession closest to the ideal genotype. TPt-198 was additionally ranked among the early-flowering accessions. In addition, both GGE biplot analysis and AMMI identified ENV3 (Ibadan 2023) and ENV4 (Ikenne 2023) as the ideal environment. Together, TPt-270, TPt-198 and TPt-187 represent the most promising accessions identified in this study and are recommended as elite parental lines for winged bean improvement programmes targeting superior seed yield.

## 1 Introduction

Winged bean (*Psophocarpus tetragonolobus)* is a versatile underutilized tropical legume with significant potential for enhancing food and nutritional security (Chinnadurai and Devi, 2025). It is valued for its exceptional multipurpose nature, as nearly all parts of the plant are edible and highly nutritious, including the seeds, leaves, pods, flowers, and tuberous roots, a trait shared by very few legumes (Bepary et al., 2023). Consequently, it has earned names such as ‘one-species supermarket’, ‘green gold’ and ‘wonder legume’, reflecting its ability to provide a complete food diet (Soni et al., 2025). Although currently grown mainly in marginal areas of Asia, Africa, and Latin America, the crop shows strong promise for widespread cultivation in the tropics and sub-tropics (Mahobia et al., 2024).

Winged bean is rich in various nutrients, including protein, carbohydrates, fats, multiple minerals such as calcium, iron, phosphorus, zinc, and copper and vitamins such as vitamin C, vitamin A, vitamin E, vitamin B6, thiamine, riboflavin, niacin, and folate, as well as edible dietary fibre (Bepary et al., 2023; Olaifa et al., 2022). The protein content of winged bean seed is approximately 38.1%–45%, and oil content of 15%-20%, comparable to that of soybean and a significantly higher lysine content (Sriwichai et al., 2021). In addition to its nutritional value, winged bean is well adapted to tropical climates and contributes to soil fertility through biological nitrogen fixation (Raai et al., 2020). Given the problem of food and nutritional insecurity, and increase demand for alternative and sustainable protein sources, winged bean represents a promising candidate for improving dietary diversity and addressing nutritional insecurity (Kaur et al., 2025). In Southeast Asia, the crop has been reported to improve the livelihoods of smallholder farmers who cultivate it (Mahobia et al., 2024). Furthermore, data on production shows that winged bean produces 2000 kg ha^-1^ of dry seeds and up to 10, 000 kg ha^-1^ of young green pods, but has the potential to produce 6000 kg ha^-1^ of dry seeds, a gap that genetic improvement could close (Dian Rakhmad and Syukur, 2024; Owusu et al., 2021).

Despite its benefits, the crop remains underutilized due to low production, inconsistent yield and few improved varieties (Wong et al., 2015). Quantifying genetic variability through coefficients of variation, broad-sense heritability and genetic advance is a prerequisite for effective selection, since these parameters jointly indicate the extent of genetic variation, the proportion of phenotypic variation that is heritable and the expected improvement in trait following selection (Adhikari et al., 2018; Akinyosoye et al., 2025). Beyond variability estimates, understanding the relationships among yield and yield-related traits is essential for simultaneous improvement of multiple characters (Ajayi et al., 2018). Collectively, knowledge of genetic variability, heritability, inter-trait relationships and stability forms the foundation for evaluating winged bean accessions based on yield performance and for identifying stable, high-performing genotypes suitable for selection and future breeding programs.

Characterisation of winged bean germplasm has advanced considerably over the past decade, though the resources examined remain modest in size. Morphological and agronomic characterisation has been the dominant approach: Bhadmus et al. (2023) evaluated 30 accessions for 17 agro-morphological and seed morphometric traits and resolved three distinct clusters, while Yulianah et al. (2020) reported substantial morphological variation among 21 Indonesian accessions and identified pod and seed traits as the principal contributors to diversity. Adegboyega et al. (2021) extended characterisation to nodulation and nitrogen fixation of 25 winged bean accessions, and Tanzi et al. (2019) examined the developmental relationship between plant architecture and yield components. More recently, Rakshitha et al. (2025) assessed phenotypic variation and genotypic variance component of 28 winged bean accessions for crop improvement purposes. Molecular characterisation has been comparatively limited; Mohanty et al. (2013) combined molecular, biochemical and physiological markers to differentiate 95 accessions using AFLP and ITS, Olawuyi et al. (2025) assessed 20 winged bean accessions using ISSR and SCoT markers, and Shonde et al. (2023) evaluated the genetic diversity of 15 accessions using microsatellite markers. Collectively, these studies establish that winged bean germplasm harbours appreciable diversity for pod and seed yield traits, that yield components are strongly inter-correlated, and that flowering time is negatively associated with yield.

Evidence on the stability of winged bean diversity across environments is markedly thinner. Soni et al. (2022) and Tiwari et al. (2022) reported stability analyses for yield and yield components traits in winged bean, and Dian and Syukur (2024) assessed several lines across three environments, and Akinyosoye et al. (2023) applied GGE biplot analysis to grain yield in 20 accessions in 6 environments. These studies are nevertheless constrained by small panels, few test environments and short evaluation periods, which limits the statistical power available to separate genotype main effects from genotype × environment interaction and increases the risk that stable, high-performing accessions are overlooked. Genotype × environment interaction (GEI), arising from the interplay between genetic and environmental factors, strongly influences trait expression and must be understood if stable, high-yielding genotypes are to be identified (Mushtaq et al., 2021), particularly for underutilized crops such as winged bean where information on environmental responses is limited (Soni et al., 2022). Where GEI is non-significant, mean performance across environments adequately represents genotypic performance; where significant GEI occurs, and especially where interactions are of the crossover type, genotype rankings change across environments and formal stability analysis becomes necessary (Khan et al., 2021). Comparable constraints have been addressed in other underutilised legumes by evaluating larger panels across contrasting agroecologies: multi-environment AMMI and GGE analyses in Bambara groundnut (Khan et al., 2021; Linus et al., 2023; Esan et al., 2023) and cowpea (Kindie et al., 2022; Mekonnen et al., 2022; Sansa et al., 2025) have successfully delineated mega-environments, identified ideal test sites and recommended broadly adapted genotypes. No equivalent evaluation has yet been reported for winged bean in sub-Saharan Africa, where the crop’s potential contribution to dietary protein supply is greatest but where information on its response to the region’s contrasting agroecologies is essentially absent.

This present study addresses these gaps by evaluating 110 winged bean accessions for 21 agronomic and yield-related traits across six environments spanning four agroecological zones in Nigeria, from humid forest to Sudan savannah. This represents, to our knowledge, the largest multi-environment evaluation of winged bean germplasm reported for the region, and provides the statistical power required to partition genotype, environment and interaction effects, to identify accessions combining high seed yield with broad adaptation, and to determine which test environments are most informative for future selection. Specifically, this study aimed to (i) estimate the genetic variability, heritability and genetic advance for seed yield, pod yield, flowering and maturity traits in winged bean; (ii) establish inter-trait relationships among seed yield, pod yield, flowering and maturity traits; and (iii) identify high-yielding, stable accessions and the most ideal test environments using AMMI and GGE biplot analyses.

## 2 Materials and methods

### 2.1 Study location

Field experiments were conducted during the rainy season of the years 2022 and 2023 at four research stations of IITA located in Nigeria (**Figure 1**), except for Kano and Mokwa, which were planted for only one year (2023) due to unforeseen circumstances. The environments spanned a considerable range of conditions in terms of temperature, rainfall, soil structure, and soil pH. Details of environmental conditions are presented in **Table 1**. Composite soil samples were collected from each experimental site after land preparation. At each location, 15 cores were taken at random across the trial area at a depth of 20 cm using a soil auger, bulked into a single composite sample per site, air-dried, gently crushed and passed through a 2 mm sieve before analysis. Ibadan and Ikenne were sampled in both 2022 and 2023, and the values reported in **Table 2** are the means of the two seasons; Kano and Mokwa were sampled in 2023 only. All soil analyses were carried out at the Soil Microbiology Laboratory, International Institute of Tropical Agriculture, Ibadan, in two analytical replicates per sample, and the reported values are means of these replicates. Soil pH was measured using a glass electrode. Particle size distribution was determined by the hydrometer method (Bouyoucos, 1962). Soil organic carbon was determined using Walkley & Black method (Walkley and Black, 1934), while total nitrogen was determined by Kjeldahl digestion following Anderson and Ingram (1989). Available phosphorus was extracted from the soil using Bray-1 solution (Bray and Kurtz, 1945) and quantified using the molybdenum blue method (Murphy and Riley, 1962). Potassium (K), calcium (Ca), magnesium (Mg), sodium (Na), zinc (Zn), copper (Cu), manganese (Mn) and iron (Fe) were determined following nitric acid–hydrogen peroxide (HNO_3_/H_2_O_2_) digestion and quantified using inductively coupled plasma optical emission spectrometry (ICP-OES) (Sah and Miller, 1992). The resulting soil physical and chemical properties are presented in **Table 2**.

**Figure 1:**
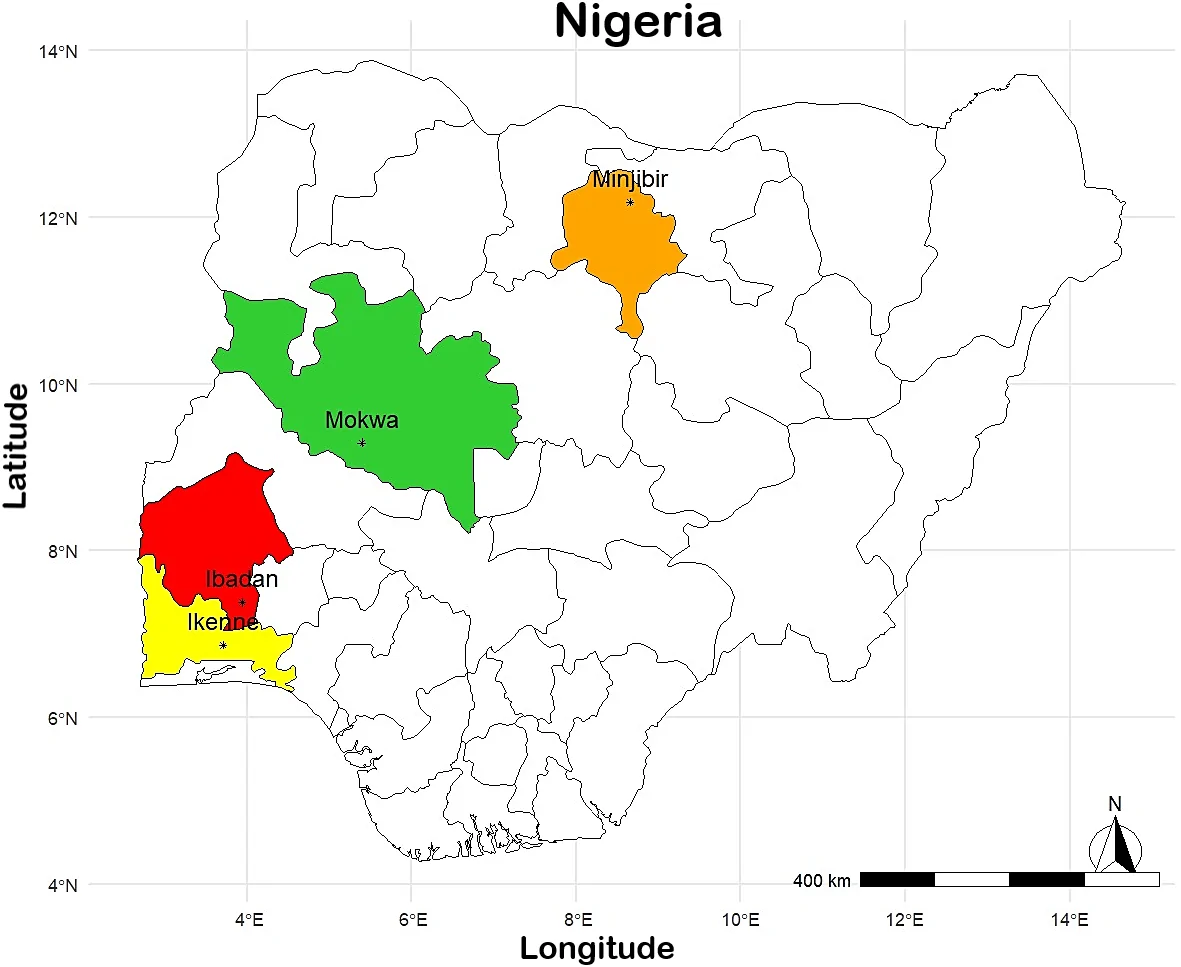
Map showing the geographical locations of the experimental sites.

**Table 1:** Environmental description of experimental locations.

Env	Location	Year	Agroecology	Latitude	Longitude	Altitude (m)	Rainfall (mm)	Mean temperature (°C)	Mean relative humidity (%)
ENV1	Ibadan	2022	Derived savannah	7° 29’N	3° 54’E	230	698	26	73
ENV2	Ikenne	2022	Humid forest	6° 53′N	3°42′E	91	1279	25	81
ENV3	Ibadan	2023	Derived savannah	7° 29’N	3° 54’E	230	956	27	76
ENV4	Ikenne	2023	Humid forest	6° 53′N	3° 42′E	91	1208	26	82
ENV5	Kano	2023	Sudan savannah	12° 10′N	8° 39′E	442	294	28	52
ENV6	Mokwa	2023	Southern Guinea savannah	9° 18’N	5° 4’E	181	598	27	69

*Rainfall values are cumulative totals over the cropping period (July–December); temperature and relative humidity are means over the same period. Trials were planted in early July in all environments. Kano and Mokwa were evaluated in 2023 only. Climatic data were obtained from IITA GIS Unit. Monthly climate series are in **Supplementary Table S1**.

**Table 2:** Characterisation of soil properties of the experimental locations.

Physical analysis	Ibadan	Ikenne	Mokwa	Kano
Sand (%)	77	83	77	87
Silt (%)	8	4	12	4
Clay (%)	15	13	11	9
Soil type	Ferric Luvisol	Eutric nitrisol	Ferrisol	Regosols
Chemical analysis	Value			
pH	6.69	4.89	5.58	6.09
Organic Carbon (%)	0.84	0.55	0.12	0.03
Total Nitrogen (%)	0.19	0.12	0.05	0.02
Available Phosphorus (mg kg^-1^)	36.42	31.03	8.00	9.80
Total Potassium (cmol^+^ kg^-1^)	2.67	1.82	0.16	1.29
Total Calcium (cmol^+^ kg^-1^)	16.33	1.53	1.33	4.35
Total Magnesium (cmol^+^ kg^-1^)	4.14	1.54	0.26	1.25
Total Sodium (cmol^+^ kg^-1^)	0.09	0.10	0.08	0.09
Total Zinc (mg kg^-1^)	108.15	53.84	22.37	18.92
Total Copper (mg kg^-1^)	13.45	7.60	2.62	5.77
Total Manganese (mg kg^-1^)	296.65	140.91	131.40	34.43
Total Iron (mg kg^-1^)	501.97	241.02	223.7	102

Soil properties are reported per location. Values for Ibadan and Ikenne are means of samples collected in 2022 and 2023 and therefore apply to both the 2022 and 2023 environments at those sites; Kano and Mokwa were sampled in 2023 only.

### 2.2 Sources of materials, land preparation, experimental design and land maintenance

One hundred and ten accessions of winged bean from diverse locations used for this study were obtained from the Genetic Resources Centre, IITA in Ibadan, Asian Vegetable Research and Development Centre in Taiwan, Agrobiodiversity and Environment Research Centre in Malaysia and Dr Graham Eagleton in Australia (**Figure 2**). The experiment was laid out in a 10 × 11 alpha lattice design in three replicates in each location. Each accession was planted in three plots of 3 m with 12 stands having a spacing of 1 m within rows and 0.75 m between rows. The land was prepared by ploughing once, harrowing twice, and ridges were done two weeks after harrowing. Pre-emergence herbicide (200 ml), lifeline, with active ingredient glufosinate-ammonium, was sprayed immediately after planting using a 20-litre knapsack sprayer. Fertilizer (5 g of NPK 15:15:15 and TSP), manual weeding, and staking were done at 3, 4, and 6 weeks after planting respectively. After which manual weeding was done at intervals when necessary until harvest. Insecticide application was done using a 20-litre knapsack sprayer filled with 200ml of Lambda-cyhalothrin + Imidacloprid 30.5% SC + mancozeb (for fungi infestation) to ward off insects like termites, beetles, worms, and thrips at four weeks after planting and once every week until harvest (Eagleton, 2020).

**Figure 2:**
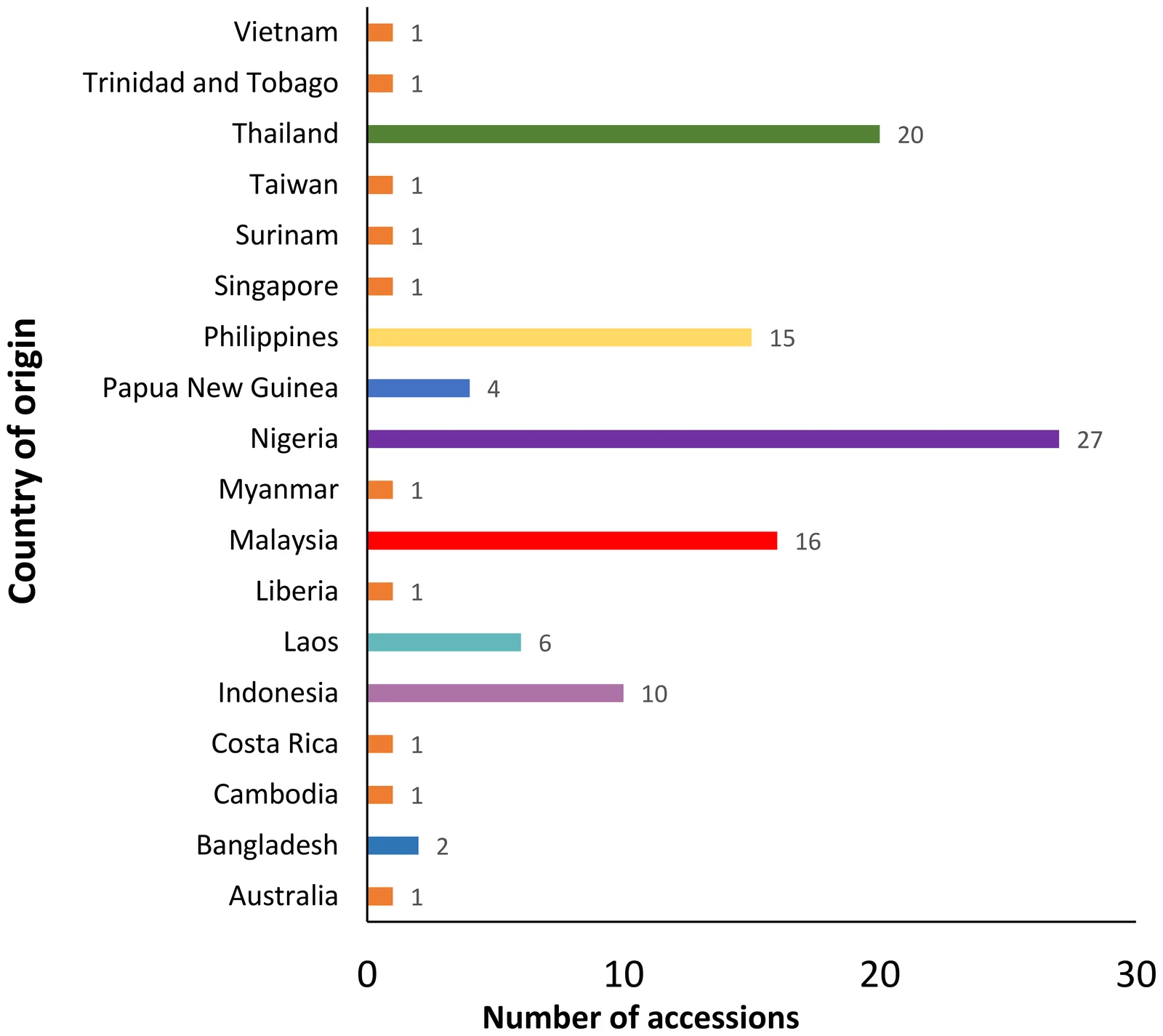
Number of winged bean accessions by country of origin (n = 110).

### 2.3 Data collection

Phenotypic data were recorded based on revised winged bean descriptors (IBPGR, 1982). Some agronomic and yield traits collected were days to 50% flowering, 50% pod emergence, seeds per pod, pods per plant, dry pod length, and hundred-seed weight. Full descriptions of the mode of observation and method of evaluation for each trait are provided in **Supplementary Table S2**. Seven stands per accession were sampled for post-harvest data.

### 2.4 Statistical analysis

#### 2.4.1 Analysis of variance

A combined analysis of variance across the six environments was performed on plot-level data using the alpha lattice linear model, fitted with the aov function in the stats package of R version 4.3.3 (R Core Team, 2023). The model was:


$${\text{Y}}_{ijkl}=\text{µ}+{\text{E}}_{i}+{\text{R}}_{j(i)}+{\text{B}}_{k(ij)}+{\text{G}}_{l}+{\left(\text{GE}\right)}_{li}+{\text{ϵ}}_{ijkl}$$

where Y*_ijkl_* is the observed value of accession l in block k within replicate j of environment i; µ is the grand mean; E*_i_* the effect of the ith environment; R*_j(i)_*the effect of replicate j nested within environment i; B*_k(ij)_*the effect of incomplete block k nested within replicate j and environment i; G*_l_* the effect of accession l; (GE)*_li_* the accession × environment interaction; and ϵ*_ijkl_* the residual error.

Each location–year combination was treated as a discrete environment. For the analysis of variance, all effects were treated as fixed, and differences were declared significant at p < 0.05. A second model, in which accession and accession × environment were treated as random effects, was fitted for the estimation of variance components and genetic parameters described in Section 2.4.2. Although Kano and Mokwa were established in 2023 only, each location–year combination was treated as a discrete environment and no location, year, or location × year terms were fitted. The analysis therefore rests on a complete 110 × 6 accession × environment array in which every accession was represented in all six environments in three replicates of the same 10 × 11 alpha lattice, so that accession means were estimated with equal replication and equal precision in each environment and each environment contributed equal residual degrees of freedom to the pooled error. Because the two-way table of environment-centred accession means submitted to singular value decomposition was likewise complete, the incomplete location × year structure did not affect the orthogonality of the AMMI and GGE decompositions or the resulting principal component structure. To confirm that the conclusions were not contingent on the unequal representation of seasons across locations, a sensitivity analysis was additionally performed on the balanced subset of four environments evaluated in 2023 (ENV3–ENV6), applying the same AMMI and GGE procedures described below.

#### 2.4.2 Genetic parameters

Genotypic, phenotypic and genotype × environment variance components were estimated by restricted maximum likelihood using the StatgenSTA and StatgenGxE packages (van Rossum, 2025) with accession and accession × environment fitted as random effects. The components correspond to the following structure, in which the phenotypic variance is expressed on an accession-mean basis:

a. Genotypic variance (σ^2^g) = $\frac{\text{MSg }-\text{ MSge}}{r\times L}$

b. Phenotypic variance (σ^2^p) = σ^2^g + $\frac{\sigma 2ge}{L}\;$+ $\frac{\text{σ}2\text{e}}{r\times L}$

c. Variance associated with genotype × environment interaction (σ^2^ge) = $\frac{\text{MSge }-\text{ MSe}}{r}$

d. Genotypic coefficient of variation (GCV) = $\frac{\surd \text{σ}2\text{g}}{\bar{\text{x}}}\;\times \;100$

e. Phenotypic coefficient of variation (PCV) = $\frac{\surd \text{σ}2\text{p}}{\bar{\text{x}}}\;\times \;100$

Where MSg = mean squares of genotype, MSge = mean squares of genotype by environment, r = number of replications, L = number of environments, σ²e = MSe = mean squares of error, and $\bar{\text{x}}$ is the grand mean for the phenotypic traits. The estimated values of PCV and GCV were categorised as described by Khan et al. (2020): 0–10% for low, 10–20% for intermediate, and ≥ 20% for high.

Broad-sense heritability (H^2^) was computed as the ratio of the genetic variance to the phenotypic variance (Hallauer et al., 2010);

f. H^2^ = $\frac{\sigma 2g}{\sigma 2g+\frac{\text{σ}2\text{ge}}{L}+\frac{\text{σ}2\text{e}}{r\times L}}$ × 100

Heritability was classified as 0–30% for low, 30–60% for intermediate, and >60% as high (Johnson et al., 1955).

The genetic advance at 5% selection intensity (GA) and genetic advance as a percentage of the mean (GAM) were estimated and described using Johnson et al. (1955) classification scale. The GAM was classified as follows: 0–10% as Low, 10–20% as Moderate, and 20% and above as High, as described in Falconer (1996).

g. GA = k × H^2^ × σp

h. GAM = (GA/μ) × 100

Where σp is the phenotypic standard deviation, respectively, k is a constant value at a selection intensity of 5% (k = 2.06); H^2^ is broad-sense heritability and μ is the grand mean.

#### 2.4.3 Correlation analysis

Phenotypic correlation coefficients (rp) among traits were estimated using Pearson correlations of accession means adjusted for environment, computed in R version 4.3.3 (R Core Team, 2023). Genotypic correlation coefficients (rg) were estimated as Pearson correlations among best linear unbiased predictors (BLUPs) of accession performance, obtained from the multi-environment mixed model fitted in the StatgenGxE package (van Rossum, 2025) with accession as a random effect and averaged across the six environments. The genotypic correlations, therefore, reflect genetic associations among traits independently of environmental and interaction sources of variation. Since phenotypic trait relationships may nonetheless vary across environments, Pearson correlation coefficients were additionally computed separately within each of the six environments.

#### 2.4.4 Multivariate analysis

Principal component analysis was performed on standardised accession means for the 21 traits and visualised using the factoextra package in R. Components with eigenvalues greater than one were retained. Hierarchical cluster analysis was performed on Gower distances using Ward’s minimum variance method (Ward’s D2) with the same package, and accessions were grouped at a 70% similarity threshold.

#### 2.4.5 Stability analysis

To identify the ideal genotype across each testing environment with high performance and stability, mega-environments, and to explain the G × E interaction, multivariate stability analysis was performed graphically based on GGE biplot and AMMI using R statistical software version 4.3.3 developed by the R Core Team. The multi-environment trial analysis (metan) was used to obtain the GGE biplots, while the Agricolae package was used for AMMI. Both analyses were performed on the two-way table of accession × environment means computed from plot-level data. Best linear unbiased predictors were used for the estimation of variance components and genetic parameters (Section 2.4.2) but not for the stability analyses, since the shrinkage applied in BLUP estimation reduces between-environment variation and would attenuate the environment main effect in the AMMI decomposition.

Accessions were ranked for SWPP within each environment, and a rank-sum index was computed across environments for each accession. The 20 highest-ranking and 10 lowest-ranking accessions were selected for stability analysis (Sansa et al., 2025), and the accession codes used throughout are given in **Supplementary Table S3**. The AMMI model partitions the accession × environment interaction by singular value decomposition into interaction principal component axes (IPCA), with accessions having IPCA 1 scores close to zero regarded as stable across environments. GGE biplot analysis was based on environment-centred data, such that the biplot displays accession main effects together with accession × environment interaction. Biplots were interpreted to identify which-won-where patterns and mega-environments, to rank accessions for mean performance and stability, and to evaluate test environments for discriminating ability and representativeness.

## 3 Results

### 3.1 Summary statistics

The presence of genetic variability in a crop’s germplasm is crucial for achieving genetic gains in breeding programs, as it determines the potential for crop improvement through selection and breeding strategies (Hemal and Dodake, 2019). Summary statistics of the traits studied are presented in **Supplementary Table S4**. Traits such as NPPP, PWPP, NSPP, SWPP, TSW, and TPDW exhibited the largest ranges and coefficients of variation, indicating substantial variability among the evaluated accessions. The highest variation was observed in TSW (CV = 0.8) and TPDW (CV = 0.7), which had extremely wide ranges (0–1618.1 and 1.1–3423.2, respectively). This indicates strong variability in total seed yield among the evaluated accessions in the different environments and highlights these traits as important contributors to variations in yield. Traits such as FPF, FPP, FPMP, FPDP, PWT, PT, SL, SWT, ST, HSW, and SHP showed relatively low coefficients of variation (CV ≤ 0.2).

### 3.2 Analysis of variance for agronomic and yield traits

The combined analysis of variance (ANOVA) for six environments revealed highly significant differences (*p < 0.001*) among accessions and environments for flowering and maturity time, pod and seed yield-related parameters (**Table 3**). The effects of accession × environment interaction were significant (*p < 0.05*) for all traits studied except hundred seed weight.

**Table 3:** Analysis of variance (ANOVA) for agro-morphological traits in winged bean.

Traits	Accession	Environment	Rep (environment)	Block (Rep × environment)	Accession × environment	Error
DF	109	5	12	180	535	952
FPF	423***	24174***	809***	69**	75***	53
FPP	295***	15100***	1337***	62	93***	53
FPMP	293***	29040***	2511***	114***	132***	75
FPDP	193***	13146***	979***	72***	95***	46
NPPP	222***	49278***	2180***	110**	106**	85
PWPP	8834***	2157718***	108705***	4782***	3943*	3350
PW	7***	707***	11***	1	2*	1
PL	3***	1834.5***	28***	3	4***	3
PWT	2***	69***	10***	1**	1***	1
PT	2***	257***	28***	1	1	1
TPDW	414502***	127469133***	2445709***	212522***	149670*	128751
NSPPD	7***	481***	8***	2	2***	2
SWPPD	3***	168***	3***	0.4	1***	0.4
SL	1***	24***	4***	0.3	0.3***	0.2
SW	1***	14***	1***	0.2*	0.2**	0.1
ST	1***	42***	2***	0.2*	0.2***	0.2
NSPP	22503***	4936268***	236225***	9616*	9381**	7668
SWPP	2183***	544243***	21294***	984*	918**	760
HSW	64**	3650**	188**	30	28	25
TSW	114337***	31655888***	512934***	49254***	38393*	32996
SHP	77***	4376**	203**	32**	31**	23

*P < 0.05, significant; **P < 0.01; ***P < 0.001. FPF, Fifty Percent Flower; FPP, Fifty Percent Pod emergence; FPMP, Fifty Percent Mature Pod; FPDP, Fifty Percent Dry Pod; NPPP, Number of Pod Per Plant; PWPP, Pod weight per plant; PW, Pod Weight; PL, Pod Length; PWT, Pod Width; PT, Pod Thickness, NSPPD, Number of seeds Per Pod; SWPPD, Seed Weight Per Pod; SL, Seed Length; SWT, Seed Width; ST, Seed Thickness; NSPP, Number of Seeds Per Plant; SWPP, Seed Weight Per Plant; HSW, Hundred Seed weight; TSW, Total Seed Weight; TPDW, Total Pod Weight; SHP, Shelling Percentage.

### 3.3 Performance of agronomic and yield traits

Based on the combined mean across all environments (**Supplementary Table S5**), days to fifty percent flowering (FPF) ranged from 63.47 days (TPt-172) to 90.80 days (TPt-181), and days to fifty percent maturity (FPMP) ranged from 105.25 days (TPt-172) to 130.82 days (TPt-181). The earliest-flowering accessions were TPt-172, TPt-198, TPt-286 and TPt-260. The mean seed weight per plant (SWPP) was 80.38 g, ranging from 51.08 g (TPt-21) to 119.75 g (TPt-216), while the mean total seed weight (TSW) was 489.26 g, ranging from 252.01 g (TPt-21) to 785.45 g (TPt-216). The mean pod weight per plant (PWPP) was 173.66 g, with a range from 113.52 g (TPt-21) to 231.66 g (TPt-216), while the mean total pod weight (TPDW) was 1044.05 g, ranging from 568.17 g (TPt-171) to 1520.98 g (TPt-294). The highest number of pods per plant (NPPP) was observed in TPt-211 (39.11), whereas TPt-3 had the lowest (22.65). TPt-216 produced the highest number of seeds per plant (NSPP) (390.09), while TPt-21 produced the lowest (175.59). The number of seeds per pod (NSPPD) was highest in TPt-237 (13.42) and lowest in TPt-289 (9.44). The longest and shortest pods were observed in TPt-207 (23.70 cm) and TPt-171 (16.41 cm), respectively.

Based on mean separation by Duncan’s Multiple Range Test (**Supplementary Table S6**), TPt-172 flowered the earliest. However, its flowering time was not significantly different from that of TPt-198, TPt-286 and TPt-260, which had a high mean performance for pod weight per plant, seed weight per plant, total pod weight, number of pods per plant and number of seeds per plant. TPt-216 ranked the highest for seed weight per plant but was not significantly different from TPt-270, TPt-198, TPt-187, TPt-180, TPt-260, TPt-286 and TPt-294. In addition, TPt-270 ranked the highest for pod weight per plant but its value was not significantly different from, TPt-198, TPt-187, TPt-180, TPt-260, TPt-286, TPt-216 and TPt-294.

### 3.4 Variance components, coefficients of variation and broad-sense heritability

The estimates of GCV, PCV, broad-sense heritability, genetic advance and genetic advance as a percentage of the mean (GAM) are presented in **Table 4**. Wide ranges of phenotypic and genotypic variance were observed for all traits. The highest values of phenotypic and genotypic variance were found in total pod weight (19864.75; 10185.65) and total seed weight (6307.07; 3699.64), respectively, while the lowest were found in seed width (0.03; 0.02) and seed length (0.07; 0.05), respectively. The PCV estimates were marginally higher than the corresponding GCV values for all traits studied. The range of GCV was 1.90 to 12.42%. Moderate GCV was found for total seed weight (12.42%), while seed weight per plant (9.78%), total pod weight (9.67%), number of seeds per plant (9.44%) and number of pods per plant (8.25%) showed low GCV. The range of PCV was 2.33 to 16.22%. Moderate PCV was observed for total seed weight (16.22%), followed by seed weight per plant (13.82%), total pod weight (13.50%), number of seeds per plant (13.17%), pod weight per plant (11.91%) and number of pods per plant (11.91%). Low PCV was recorded for seed width (2.33%), followed by seed length (3.12%).

**Table 4:** Estimates of genetic variability and broad-sense heritability for agro-morphological traits.

Traits	VE	VG	VGE	VP	GCV	PCV	H^2^ (%)	GA at 5%	GA over mean at 5%
FPF	92.31	32.73	21.67	41.47	7.43	8.37	78.92	10.48	13.63
FPP	102.30	22.80	23.84	32.46	5.46	6.51	70.25	8.26	9.43
FPMP	88.28	13.99	25.43	23.13	3.16	4.06	60.48	6.00	5.07
FPDP	100.41	16.53	31.97	27.44	3.01	3.88	60.25	6.51	4.82
NPPP	88.33	6.34	11.84	13.22	8.25	11.91	47.96	3.60	11.78
PWPP	3653.89	180.48	293.86	432.45	7.70	11.91	41.73	17.90	10.26
PW	1.27	0.31	0.09	0.39	8.18	9.26	78.03	1.00	14.91
PL	3.48	1.52	0.41	1.78	6.36	6.89	85.31	2.35	12.13
PWT	1.96	0.05	0.12	0.18	2.08	3.94	27.85	0.24	2.27
PT	0.98	0.10	0.04	0.16	3.28	4.15	62.22	0.51	5.33
NSPPD	2.04	0.28	0.30	0.45	4.92	6.18	63.41	0.87	8.08
SWPPD	0.45	0.07	0.04	0.10	7.81	9.48	68.00	0.44	13.29
SL	0.35	0.05	0.02	0.07	2.59	3.12	68.65	0.38	4.43
SW	0.20	0.02	0.01	0.03	1.90	2.33	66.07	0.25	3.18
ST	0.30	0.02	0.03	0.04	2.13	2.98	50.91	0.22	3.13
NSPP	8231.62	652.27	963.29	1270.13	9.44	13.17	51.35	37.75	13.96
SWPP	845.19	62.62	93.94	125.24	9.78	13.82	50.00	11.54	14.26
HSW	33.25	2.15	1.29	4.21	4.77	6.67	50.99	2.16	7.02
TSW	33601.41	3699.64	4444.10	6307.07	12.42	16.22	58.66	96.10	19.62
TPDW	127613.72	10185.65	15536.67	19864.75	9.67	13.50	51.28	149.10	14.29
SHP	19.88	2.98	0.79	23.65	3.82	10.77	63.83	31.10	68.86

VE, Environmental Variance; VG, Genotypic Variance; VGE, Genotype by Environment Variance; VP, Phenotypic Variance; GCV, Genotypic Coefficient of Variation; PCV, Phenotypic Coefficient of Variation; H^2^%, Broad Sense Heritability; GA, Genetic Advance. FPF, Fifty Percent Flower; FPP, Fifty Percent Pod emergence; FPMP, Fifty Percent Mature Pod; FPDP, Fifty Percent Dry Pod; NPPP, Number of Pod Per Plant; PWPP, Pod weight per plant; PW, Pod Weight; PL, Pod Length; PWT, Pod Width; PT, Pod Thickness; NSPPD, Number of seeds Per Pod; SWPPD, Seed Weight Per Pod; SL, Seed Length; SWT, Seed Width; ST, Seed Thickness; NSPP, Number of Seeds Per Plant; SWPP, Seed Weight Per Plant; HSW, Hundred Seed weight; TSW, Total Seed Weight; TPDW, Total Pod Weight; SHP, Shelling Percentage.

Genetic advance (GA) varied from 0.22 for seed thickness to 149.10 for total pod weight. Genetic advance as a percentage of the mean at 5% selection intensity varied from 2.1% for shelling percentage to 19.62% for total seed weight. Moderate GAM was recorded for pod weight per plant (10.26%), number of pods per plant (11.78%), seed weight per plant (14.26%), total pod weight (14.29%), number of seeds per plant (13.96%), total seed weight (19.62%), seed weight per pod (13.29%), pod weight (14.91%), days to fifty percent flowering (13.63%) and pod length (12.13%)), while the remaining traits showed low GAM (**Supplementary Figure S1**).

Broad-sense heritability ranged from 27.85% to 85.31% (**Supplementary Figure S2**). The highest heritability was recorded for pod length (85.31%), followed by days to fifty percent flowering (78.92%), pod weight (78.03%) and days to fifty percent pod emergence (70.25%).

Pod width (27.85%) was the only trait classified as low (< 30%); all other traits fell within the intermediate (30–60%) or high (> 60%) classes. High heritability coupled with moderate GAM was recorded for pod length, days to fifty percent flowering, pod weight and seed weight per pod. High heritability with low GAM was recorded for days to fifty percent pod emergence, seed length, seed width, shelling percentage, number of seeds per pod, pod thickness, days to fifty percent mature pod and days to fifty percent dry pod. Moderate heritability with moderate GAM was recorded for total seed weight, number of seeds per plant, total pod weight, seed weight per plant, number of pods per plant and pod weight per plant, while moderate heritability with low GAM was recorded for hundred seed weight and seed thickness. Low heritability and low GAM were recorded for pod width.

### 3.5 Principal component analysis of agronomic and yield traits of winged bean accessions

The mean data of all studied traits were analysed by principal component analysis to determine the contribution of each trait to total variability (**Table 5**). The total variance was divided into 20 components, of which the first five displayed eigenvalues greater than one. The elbow of the scree plot (**Supplementary Figure S3**) occurred at the fifth PC. The eigenvalue for the first PC was highest at 7.31 and declined progressively to 1.13 for the fifth PC. The first, second, third, fourth and fifth PCs accounted for 36.53%, 21.59%, 11.67%, 7.10% and 5.63% of the total variation, respectively, together explaining approximately 83% of the total variation.

**Table 5:** Eigenvectors from principal components analysis (PCA) of traits studied.

Traits	PC1	PC2	PC3	PC4	PC5
FPF	0.01	0.36	0.33	0.12	-0.02
FPP	0.02	0.37	0.30	0.19	0.02
FPMP	-0.01	0.36	0.27	0.24	-0.10
FPDP	0.06	0.37	0.14	0.24	0.02
NPPP	-0.26	-0.22	0.22	0.21	-0.14
PWPP	-0.34	-0.11	0.14	0.08	-0.09
PW	-0.27	0.22	-0.13	-0.20	0.20
PL	-0.24	0.25	0.06	-0.34	-0.03
PWT	-0.11	0.16	-0.16	0.17	-0.35
PT	-0.04	0.03	-0.33	0.45	0.34
NSPPD	-0.21	0.14	0.05	-0.19	0.62
SWPPD	-0.29	0.19	-0.10	-0.16	0.24
SL	-0.19	0.19	-0.13	-0.35	-0.35
SWT	-0.17	0.22	-0.36	0.10	-0.20
ST	-0.14	0.06	-0.40	0.40	0.09
NSPP	-0.29	-0.21	0.24	0.11	0.06
SWPP	-0.33	-0.16	0.11	0.11	-0.02
HSW	-0.22	0.12	-0.26	-0.02	-0.24
TSW	-0.32	-0.17	0.10	0.09	0.02
TPDW	-0.33	-0.14	0.13	0.07	-0.10
Eigenvalue	7.31	4.32	2.34	1.42	1.13
Standard deviation	2.70	2.08	1.53	1.19	1.06
Proportion of Variance	0.37	0.22	0.12	0.07	0.06
Cumulative Proportion	0.37	0.58	0.70	0.77	0.83
Variability %	36.53	21.59	11.67	7.10	5.63

FPF, Fifty Percent Flower; FPP, Fifty Percent Pod emergence; FPMP, Fifty Percent Mature Pod; FPDP, Fifty Percent Dry Pod; NPPP, Number of Pod Per Plant; PWPP, Pod weight per plant; PW, Pod Weight; PL, Pod Length; PWT, Pod Width; PT, Pod Thickness; NSPPD, Number of seeds Per Pod; SWPPD, Seed Weight Per Pod; SL, Seed Length; SWT, Seed Width; ST, Seed Thickness; NSPP, Number of Seeds Per Plant; SWPP, Seed Weight Per Plant; HSW, Hundred Seed weight; TSW, Total Seed Weight; TPDW, Total Pod Weight; SHP, Shelling Percentage.

The first principal component was dominated by pod and seed yield traits, with pod weight per plant (−0.34), seed weight per plant (−0.33), total pod weight (−0.33), total seed weight (−0.32), number of seeds per plant (−0.29) and number of pods per plant (−0.26) loading strongly and in the same direction, whereas the phenological traits contributed negligibly (loadings of 0.01 to 0.06). Accessions with high PC1 scores are high yielding accessions. The contrast between phenology and yield was resolved on the second principal component, on which days to fifty percent flowering (0.36), days to fifty percent pod emergence (0.37), days to fifty percent mature pod (0.36) and days to fifty percent dry pod (0.37) loaded strongly and positively while number of pods per plant (−0.22), number of seeds per plant (−0.21), total seed weight (−0.17) and seed weight per plant (−0.16) loaded negligibly and negatively. Accessions with high PC2 scores were therefore late-flowering and late-maturing with comparatively low pod and seed yield. The principal component scores of the 110 winged bean accessions for the first five principal components, ranked by PC1 based on the accessions is presented in **Supplementary Table S7**.

### 3.6 Biplot of 110 winged bean accessions based on the seed and pod yield-related parameters, and agronomic traits

The measured traits and the accessions studied were represented on a biplot of the first and second principal components, which together accounted for a cumulative variance of 58.12% (**Figure 3**). The accessions were scattered across the four quadrants. The analysis resolved three main groups of traits: (i) FPF, FPP and FPDP; (ii) FPMP, PL, PWT, SWT, SL, PW, SWPPD, NSPPD, HSW, PT and ST; and (iii) PWPP, TPDW, SWPP, TSW, NSPP and NPPP. Accessions such as TPt-181 and TPt-176, which fell in the first quadrant, were characterised by late flowering and late maturity. Yield-related traits, namely pod weight per plant, total pod weight, seed weight per plant, total seed weight, number of seeds per plant and number of pods per plant, fell in the third quadrant. Accessions TPt-260, TPt-270, TPt-187, TPt-198, TPt-294, TPt-216 and TPt-286 clustered close to this group of yield traits.

**Figure 3:**
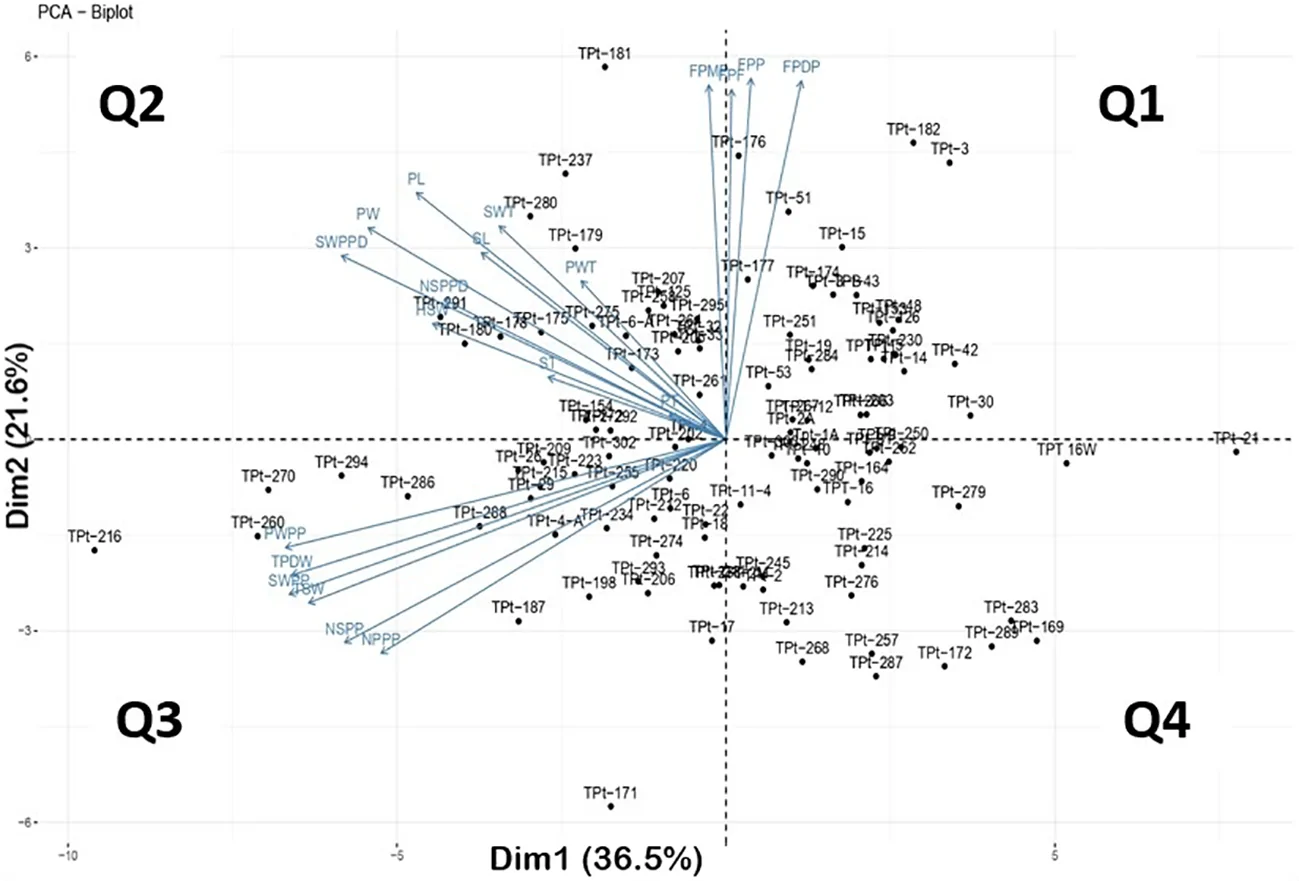
PCA biplot of PCA 1 and PCA 2 of 110 accessions of winged bean with agronomic and yield-related traits.

### 3.7 Cluster analysis of 110 winged bean based on the seed and pod yield-related parameters, and agronomic traits

The accessions were grouped into three distinct clusters at an R-squared distance of 0.7 (70% similarity index), presented as a Ward’s minimum variance hierarchical dendrogram based on Gower distance (**Figure 4**). The first, second and third clusters contained 40.0%, 28.2% and 31.8% of the accessions, respectively (**Supplementary Table S8**). Cluster 3 contained accessions that flowered and matured earliest (mean FPF 72.2 days, FPMP 115.7 days) and recorded the highest pod and seed yield (mean PWPP 192.2 g, SWPP 91.8 g). Cluster 2 comprised the latest-flowering and latest-maturing accessions (mean FPF 80.3 days, FPMP 120.9 days) with intermediate yield (mean PWPP 178.2 g, SWPP 79.4 g), while Cluster 1 was intermediate in phenology (mean FPF 74.1 days, FPMP 116.8 days) but recorded the lowest pod and seed yield (mean PWPP 155.7 g, SWPP 72.0 g). These differences are presented in **Supplementary Figure S4**.

**Figure 4:**
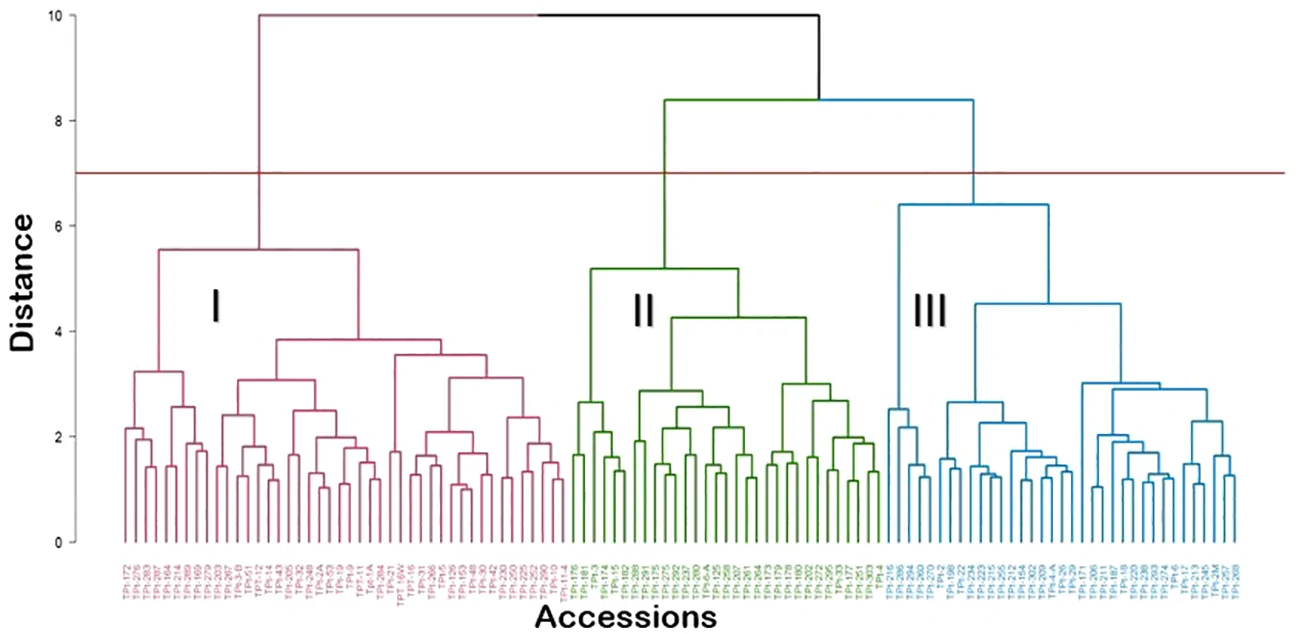
Ward’s minimum variance hierarchical cluster dendrogram of 110 Winged bean accessions evaluated during the 2022 and 2023 growing seasons in four agro-ecologies of Nigeria using Grower distance methods.

### 3.8 Phenotypic and genotypic correlation among seed yield, pod yield, flowering, and maturity time of 110 winged bean accessions

Correlation analysis helps plant breeders understand the relationship between two traits in terms of their strength and direction. This helps to guide the selection of high-yielding genotypes. Traits that have a strong and significantly positive correlation on yield can be focused on during selection process (Russinga et al., 2020). The phenotypic (rp) and genotypic (rg) correlations between the yield and agronomic traits are presented in **Supplementary Table S9**; **Table 6**, respectively. Highly significant positive correlations (p < 0.001) were found between days to fifty percent flowering and days to fifty percent pod emergence (rp = 0.92***, rg = 0.94***), days to fifty percent mature pod (rp = 0.74***, rg = 0.79***) and days to fifty percent dry pod (rp = 0.63***, rg = 0.71***). Among the pod and seed yield-related traits, highly significant and positive correlations were found. Total pod weight was positively and highly correlated with total seed weight (r*p* = 0.93***, r*g* = 0.91***), seed weight per plant (r*p* = 0.87***, r*g* = 0.84***), number of pods per plant (r*p* = 0.74***, r*g* = 0.67***), pod weight per plant (r*p* = 0.90***, r*g* = 0.87***) and number of seeds per plant (r*p* = 0.81***, r*g* = 0.78***). Seed weight per plant and number of seeds per plant were positively and highly correlated with pod weight per plant (r*p* = 0.89***, r*g* = 0.87*** and r*p* = 0.83***, r*g* = 0.81***, respectively) and with number of pods per plant (r*p* = 0.76***, r*g* = 0.71*** and r*p* = 0.85***, r*g* = 0.84***, respectively).

**Table 6:** Genotypic correlation coefficients for seed, pod yield-related traits of 110 accessions of winged bean.

Trait	FPF	FPP	FPMP	FPDP	NPPP	PWPP	PW	PL	PWT	PT	NSPPD	SWPPD	SL	SWT	ST	NSPP	SWPP	HSW	TSW	TPDW
FPP	.94***																			
FPMP	.79***	.81***																		
FPDP	.71***	.73***	.87***																	
NPPP	-.27**	-.21*	-.17	-.24*																
PWPP	-.20*	-.13	-.07	-.18	.72***															
PW	.17	.19	.24*	.19	-.06	.48***														
PL	.38***	.35***	.38***	.30**	-.13	.33***	.80***													
PWT	.16	.19*	.25**	.24*	-.07	.19*	.51***	.40***												
PT	-.11	-.07	-.06	-.04	.04	.14	.34***	-.04	.34***											
NSPPD	.18	.17	.13	.07	.03	.41***	.67***	.54***	.12	.19*										
SWPPD	.14	.16	.22*	.14	.02	.49***	.85***	.66***	.31***	.30**	.76***									
SL	.14	.11	.21*	.13	-.05	.29**	.49***	.59***	.27**	-.01	.19	.51***								
SWT	.05	.06	.15	.18	-.06	.31***	.58***	.39***	.44***	.38***	.13	.57***	.57***							
ST	-.17	-.11	-.03	.06	-.02	.24*	.45***	.09	.28**	.48***	.21*	.43***	.08	.63***						
NSPP	-.26**	-.19*	-.20*	-.30**	.84***	.81***	.12	-.01	-.15	.03	.36***	.26**	-.05	-.05	.05					
SWPP	-.30**	-.24*	-.17	-.25**	.71***	.87***	.35***	.15	-.05	.14	.40***	.48***	.21*	.29**	.31**	.87***				
HSW	.03	.04	.04	.08	-.01	.36***	.51***	.37***	.29**	.21*	.17	.52***	.53***	.65***	.50***	.05	.35***			
TSW	-.30**	-.23*	-.16	-.25**	.65***	.78***	.31**	.11	-.07	.09	.39***	.42***	.15	.21*	.24*	.83***	.92***	.29**		
TPDW	-.24*	-.18	-.09	-.21*	.67***	.87***	.40***	.26**	.11	.09	.34***	.41***	.24*	.25**	.19*	.78***	.84***	.30**	.91***	
SHP	-.29**	-.25**	-.28**	-.26**	.20*	.13	-.17	-.39***	-.39***	.06	.12	.15	-.13	.07	.28**	.40***	.48***	.17	.50***	.19*

*P < 0.05, significant; **P < 0.01; ***P < 0.001. FPF, Fifty Percent Flower; FPP, Fifty Percent Pod emergence; FPMP, Fifty Percent Mature Pod; FPDP, Fifty Percent Dry Pod; NPPP, Number of Pod Per Plant; PWPP, Pod weight per plant; PW, Pod Weight; PL, Pod Length; PWT, Pod Width; PT, Pod Thickness; NSPPD, Number of seeds Per Pod; SWPPD, Seed Weight Per Pod; SL, Seed Length; SWT, Seed Width; ST, Seed Thickness; NSPP, Number of Seeds Per Plant; SWPP, Seed Weight Per Plant; HSW, Hundred Seed weight; TSW, Total Seed Weight; TPDW, Total Pod Weight; SHP, Shelling Percentage.

Days to fifty percent flowering was significantly but weakly and negatively correlated with number of pods per plant (r*p* = −0.19*, r*g* = −0.27**), seed weight per plant (r*p* = −0.22*, r*g* = −0.30**) and total seed weight (r*p* = −0.23*, r*g* = −0.30**). The genotypic correlations were consistently stronger than their phenotypic counterparts in the phenology traits but the opposite in the yield-related traits. Correlation coefficients computed separately within each environment were consistent in sign with those obtained from the combined analysis for 97.6% of trait pairs showing moderate to strong association (|r| ≥ 0.5), and all major yield-component relationships were positive in every environment (**Supplementary Table S10**).

### 3.9 AMMI and GGE biplot analysis

Winged bean accessions were ranked for seed weight per plant across environments using a rank-sum index, and the 20 best-performing and 10 poorest-performing accessions were selected for the stability analysis (**Supplementary Table S3**).

The AMMI model revealed significant mean squares for genotype (G), environment (E) and genotype × environment interaction (GEI) effects for seed weight per plant (**Table 7**).

**Table 7:** Analysis of variance for additive main effects and multiplicative interaction model (AMMI) for seed yield of 30 (best 20 and poorest 10) winged bean accessions across six environments in Nigeria.

Source of variation	Degrees of freedom	Sum of squares	Mean square	Contribution to total variation (%)	F	Sig.
Genotype	29	124, 190.56	4, 282.43	8.78	3.70	***
Environment	5	1, 065, 332.62	213, 066.52	75.28	184.02	***
Interactions (G x E)	145	225, 544.92	1, 555.48	15.94	1.34	*
*IPCA 1*	33	100, 172.45	3, 035.53	44.41	2.62	***
*IPCA 2*	31	47, 159.14	1, 521.26	20.91	1.31	ns
*IPCA 3*	29	43, 801.77	1, 510.41	19.42	1.30	ns
*IPCA 4*	27	23, 613.36	874.57	10.47	0.76	ns
*IPCA 5*	25	10, 798.20	431.93	4.79	0.37	ns
**Total**	**179**	**1**, **415**, **068.10**	**—**	**100.00**	**—**	**—**

*significant at *p < 0.05*; **Significant at *p < 0.01*; ***Significant at *p < 0.001*; ns, not significant. IPCA, Interaction Principal Component Axis.

The environment effect accounted for the largest proportion of the total variation (75.28%). The genotype effect contributed 8.78% of the total variation and was highly significant (*p* < 0.001); accession means ranged almost two-fold, from 48.43 g in G35 (TPt-203) to 100.24 g in G70 (TPt-270). The GEI effect accounted for 15.94% of the total variation and was significant (*p* < 0.05), and therefore exceeded the genotype main effect. Partitioning of the GEI by singular value decomposition showed that the first interaction principal component axis (IPCA 1) explained the largest fraction of the GEI sum of squares (44.41%) and was highly significant (*p* < 0.001). IPCA 2 to IPCA 5 were all non-significant (*p* > 0.05), accounting for 20.91%, 19.42%, 10.47% and 4.79% of the GEI, respectively. Interpretation of the AMMI biplot was therefore based on IPCA 1.

The AMMI biplot displays the SWPP response of the 30 accessions across the six test environments (**Figure 5**). The vertical line represents the grand mean SWPP (77.44 g), thereby dividing the biplot into low- and high-yielding segments. The horizontal line represents an IPCA 1 value of zero, thereby dividing the biplot into above zero and below zero. Accessions are considered stable when they have above average mean and have IPCA score close to zero. G54 (TPt-238), G108 (TPt-6), G26 (TPt-180) and G12 (TPT-16) combined above-average mean SWPP values of 82.79, 80.48, 90.76 and 82.28 g with IPCA 1 scores closest to zero (−0.12, 0.38, −0.45 and 0.87, respectively).

**Figure 5:**
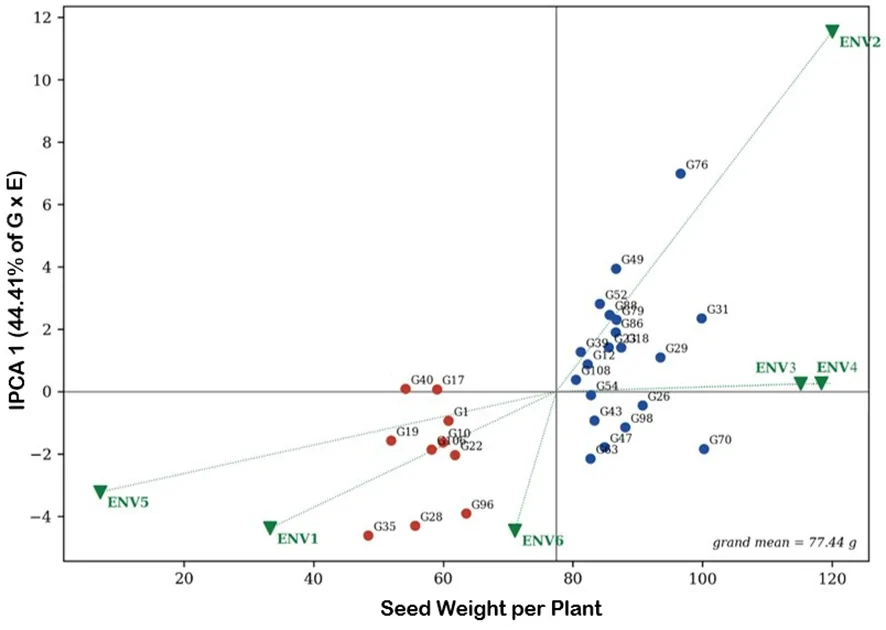
AMMI biplot analysis of seed weight per plant of 30 winged bean accessions evaluated across six environments.

G70 (TPt-270) recorded the highest mean SWPP of 100.24 g, the largest value across all 30 accessions, together with a relatively small IPCA 1 score of −1.84. G31 (TPt-198) followed with a mean SWPP of 99.87 g and an IPCA 1 score of 2.34. G29 (TPt-187) combined the fourth highest mean SWPP (93.51 g) with an IPCA 1 score of 1.09. G76 (TPt-280) recorded the third highest mean SWPP (96.62 g) but the largest IPCA 1 score of any accession evaluated (6.99).In the low-yielding segment, G35 (TPt-203) recorded the lowest mean SWPP of 48.43 g, followed by G19 (TPt-174) at 51.97 g and G40 (TPt-21) at 54.18 g; G35 additionally recorded the largest negative IPCA 1 score (−4.62). ENV2 recorded the largest positive IPCA 1 score. ENV6 and ENV1 recorded the largest negative IPCA 1 scores. ENV3 and ENV4 recorded IPCA 1 scores close to zero.

### 3.10 GGE biplot analysis of the ideal winged bean genotype

An ideal genotype sits where the greatest mean and zero perpendicular deviation mets, so it sits on the AEA itself, at the distance of the longest genotype projection (Linus et al., 2023; Olanrewaju et al., 2021). The GGE biplot ranking of the 30 accessions relative to the ideal genotype is presented in **Figure 6**. The two principal components together accounted for 73.45% of the total genotype and genotype × environment interaction variation, with PC1 contributing 54.41% and PC2 contributing 19.04%. G29 (TPt-187) was the accession closest to the ideal genotype; although its mean SWPP of 93.51 g ranked fourth among the 30 accessions, it showed the smallest deviation from the average environment axis of any high-yielding accession. G31 (TPt-198) ranked second in proximity to the ideal genotype with a mean SWPP of 99.87 g, and G70 (TPt-270) ranked third with the highest mean SWPP recorded (100.24 g). G26 (TPt-180) and G18 (TPt-173) followed, in that order.

**Figure 6:**
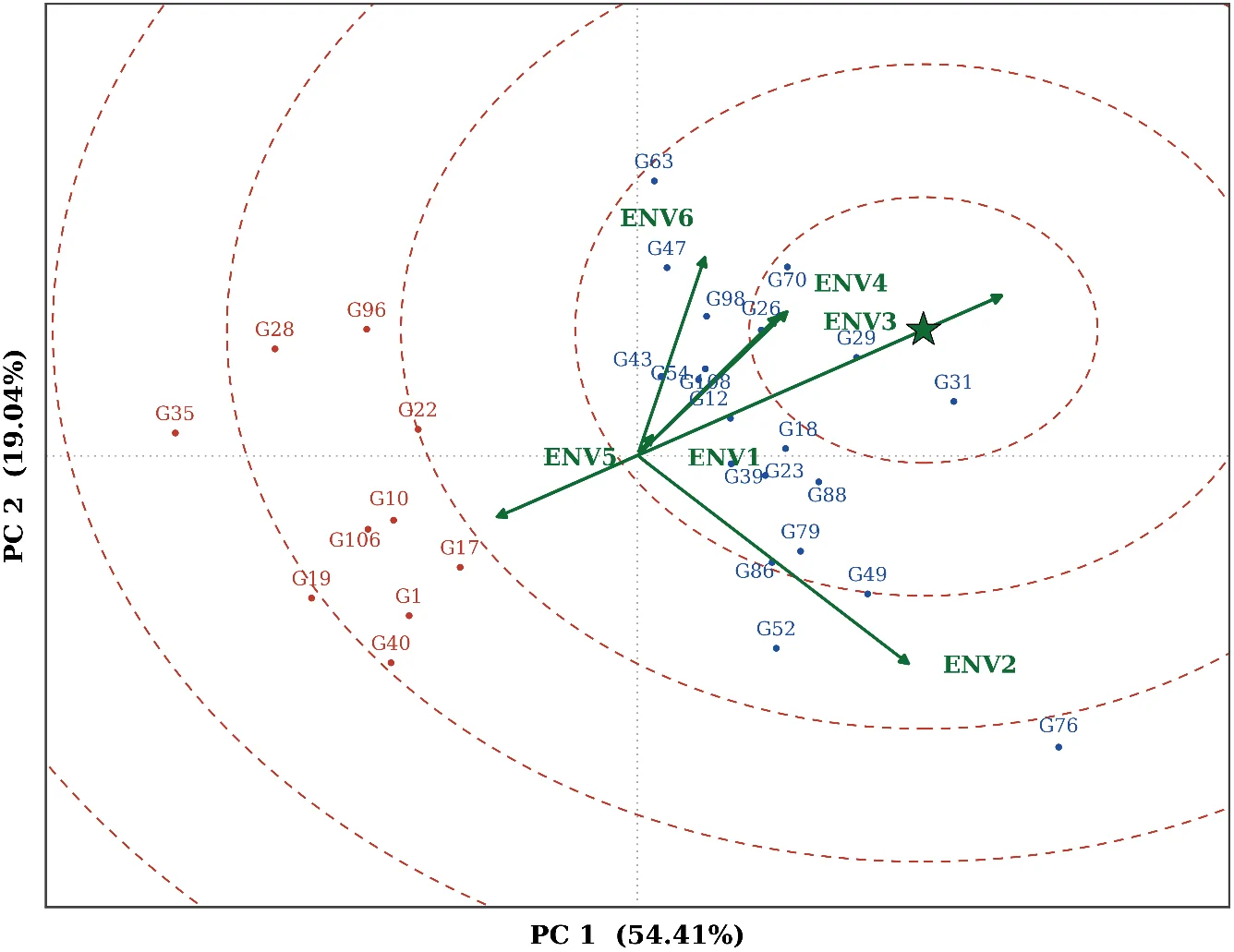
A GGE biplot showing ideal genotype based of seed weight per plant of 30 winged bean accessions (best 20 (Blue) and worst 10 (red) across 6 environments.

### 3.11 GGE biplot analysis of ideal winged bean environment

The GGE biplot view of the ideal environment for seed weight per plant (SWPP) across the 30 winged bean accessions is presented in **Figure 7**. An ideal environment is defined by a long vector length, indicating high discriminating ability, and a small angle to the average environment axis (AEA), indicating high representativeness of the overall test conditions (Ghaffari et al., 2021; Liu et al., 2026). The use of ideal environments in multi-environment trial design has been widely advocated as a cost-effective strategy for optimising genotype evaluation and accelerating genetic gains in crop improvement programmes (Gumede et al., 2022; Kindie et al., 2022; Singh et al., 2022).

**Figure 7:**
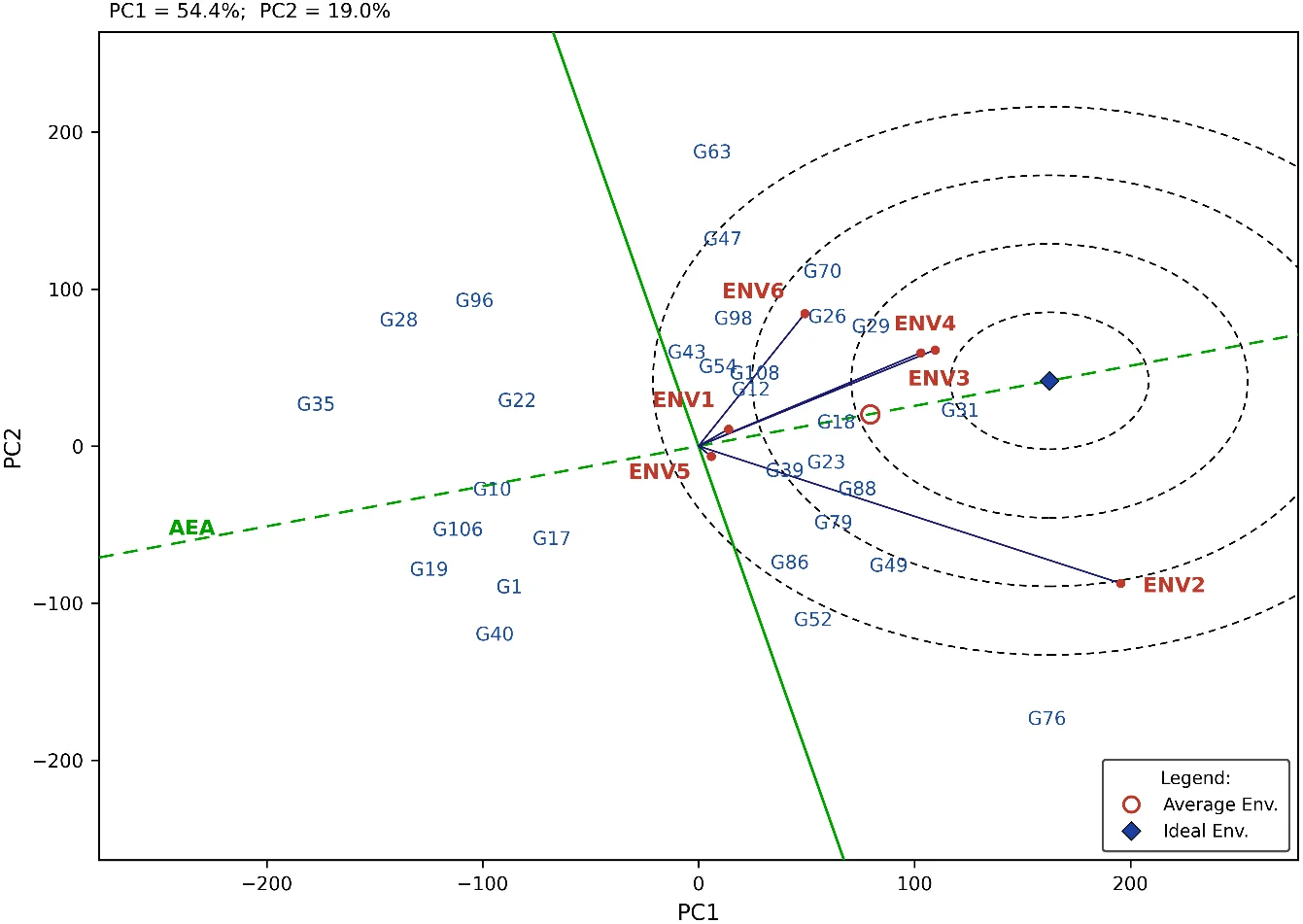
A GGE biplot showing ideal environment based of seed weight per plant of 30 winged bean accessions (best 20 and worst 10) across six environments.

ENV3 (Ibadan 2023) was identified as the environment closest to the ideal among all six test environments, combining a long vector (125.45) with the smallest angle to the AEA (14.89°) and the shortest distance to the ideal point (56.58). ENV4 (Ikenne 2023) ranked second and was almost indistinguishable from ENV3, with a vector length of 118.81, an angle to the AEA of 15.67° and a distance to the ideal point of 62.26. The near-coincidence of these two environments in the biplot indicates that they ranked the accessions in an almost identical manner, despite differing in seasonal rainfall by 251 mm and representing two distinct agroecological zones. This combination of high discriminating power and representativeness makes ENV3 and ENV4 the most suitable environments for evaluating and selecting superior winged bean accessions for SWPP.

ENV6 (Mokwa 2023) and ENV2 (Ikenne 2022) ranked third and fourth in proximity to the ideal. ENV2 recorded by far the longest environment vector (214.12), identifying it as the most discriminating of the six environments; however, its large angle to the AEA (38.42°) indicates that it was poorly representative of the average test conditions, so that selection conducted in this environment alone would favour accessions adapted to its particular conditions rather than those of broad adaptation. ENV1 (Ibadan 2022) and ENV5 (Kano 2023) were the furthest from the ideal, with very short vectors of 17.61 and 8.81, respectively. These short vectors indicate that both environments had very limited ability to discriminate among the accessions, limiting their suitability as representative test environments for broad adaptation studies in winged bean (Ghaffari et al., 2021; Liu et al., 2026).

### 3.12 GGE biplot (‘which-won-where’ pattern)

The polygon view of the “which-won-where” biplot is a crucial component of the GGE biplot analysis, which is extensively used to visualise genotype by environment interactions (GEI). This visualisation technique helps in identifying crossover interactions, differentiating mega-environments, and determining specific adaptations of genotypes (Olanrewaju et al., 2021).

The ‘which-won-where’ polygon view of the GGE biplot is presented in **Figure 8**. The vertex genotypes were G40 (TPt-21), G76 (TPt-280), G31 (TPt-198), G70 (TPt-270), G63 (TPt-26), G28 (TPt-182), G35 (TPt-203) and G19 (TPt-174). The six test environments were distributed across four sectors of the polygon. ENV3 and ENV4 fell within the sector in which G31 (TPt-198) was the vertex accession; ENV2 and ENV5 fell within the sector of G76 (TPt-280); ENV1 fell within the sector of G70 (TPt-270); and ENV6 fell within the sector of G63 (TPt-26). The vertex genotypes occupying sectors containing no environments, G35 (TPt-203), G19 (TPt-174), G40 (TPt-21) and G28 (TPt-182), recorded the lowest mean SWPP values of 48.43 g, 51.97 g, 54.18 g and 55.67 g, respectively.

**Figure 8:**
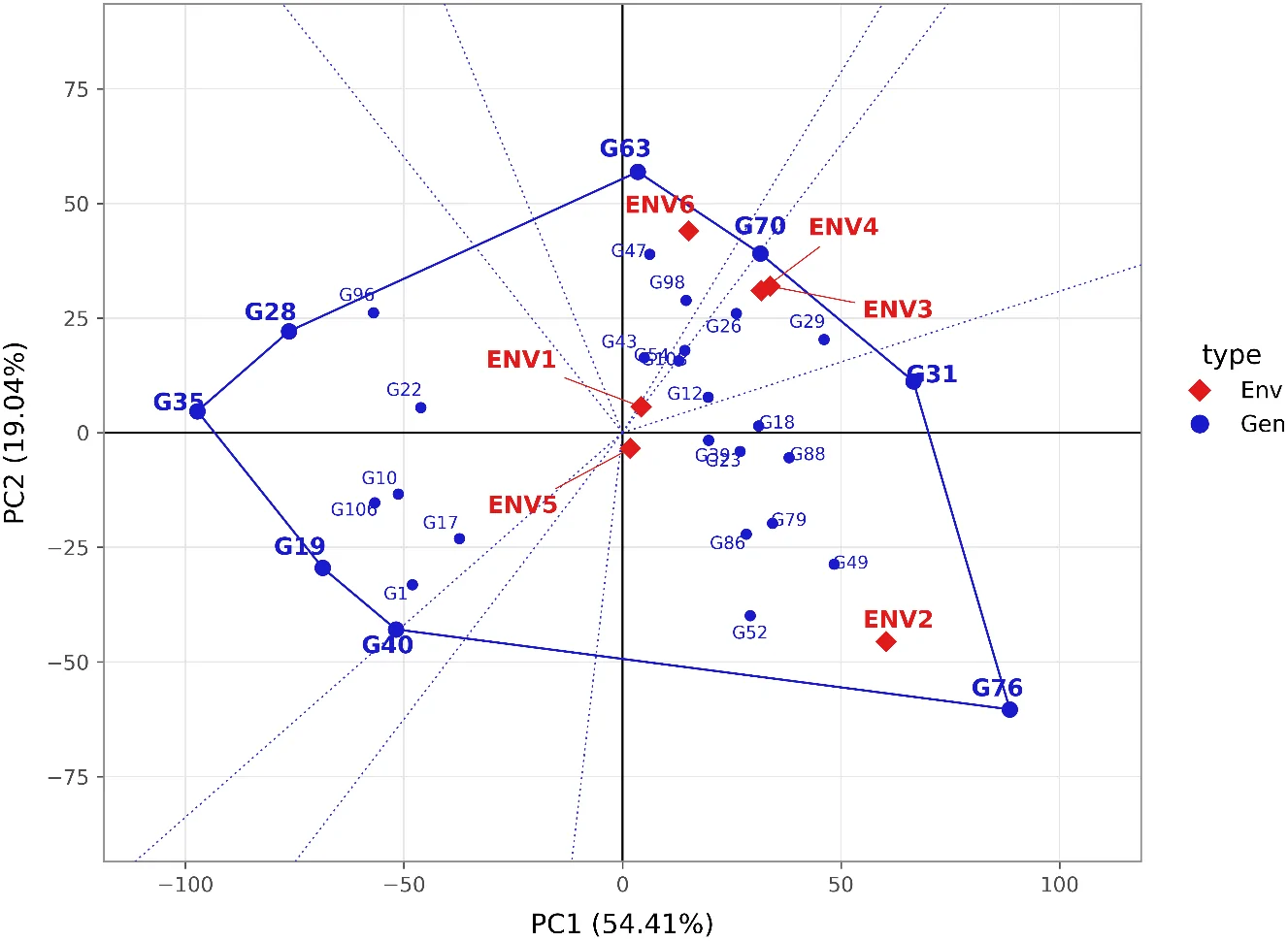
The Which Won Where view of the GGE biplot based on genotype × environment seed weight per plant data of 30 winged bean accessions.

### 3.13 ‘Discriminitiveness vs. representativeness’ pattern of GGE biplot

The determination of the most suitable test environment is a critical component of any successful multi-environment evaluation programme, as it ensures that the environments used for genotype selection are both informative and representative of the broader target region (Khan et al., 2021; Tiwari et al., 2022; Esan et al., 2023). The ‘discriminativeness vs. representativeness’ view of the GGE biplot for seed weight per plant (SWPP) of the 30 winged bean accessions across six test environments is presented in **Figure 9**.

**Figure 9:**
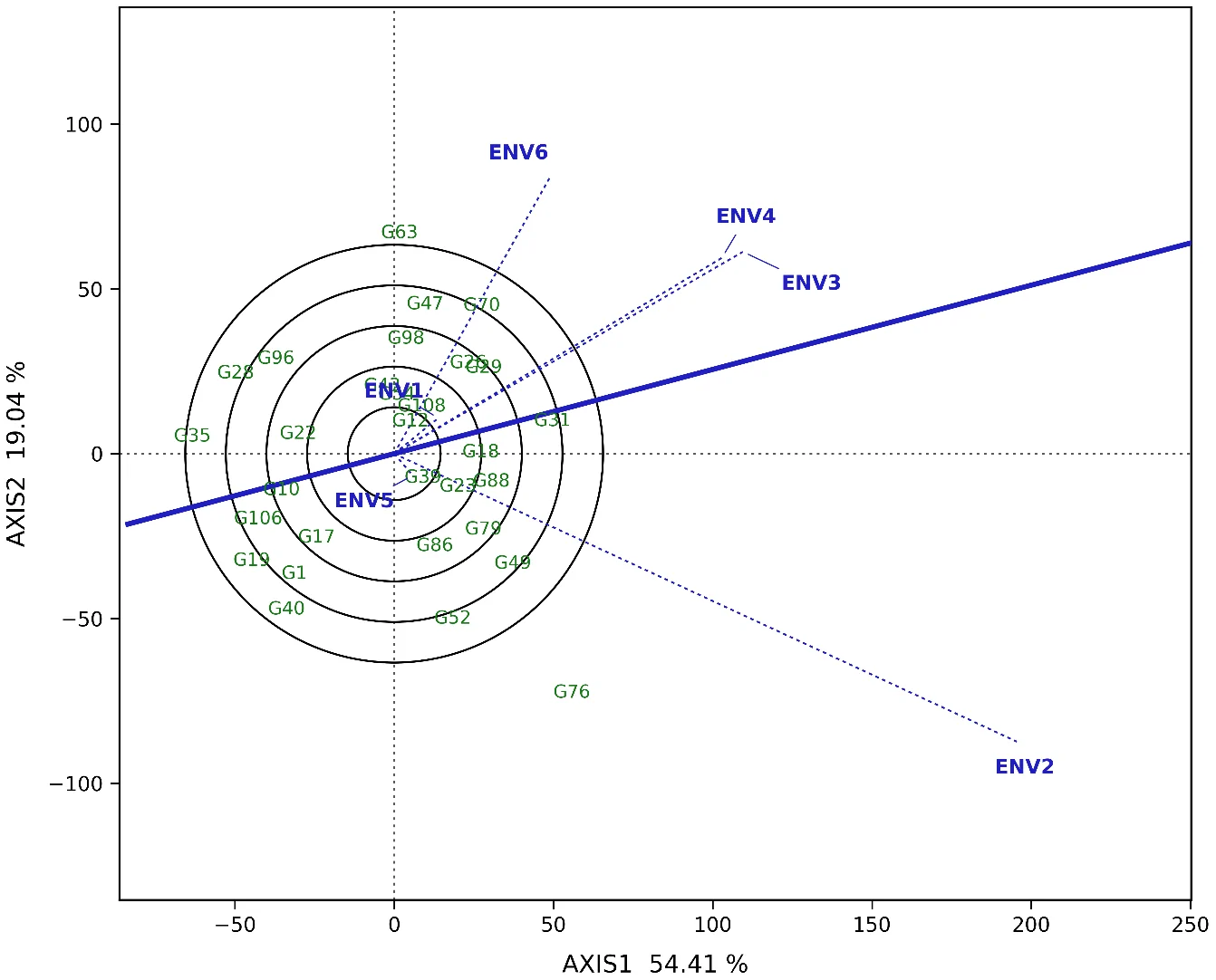
The discriminative and representativeness view of the GGE biplot based on genotype × environment seed weight per plant data of 30 winged bean accessions.

ENV2 (Ikenne 2022) had the longest environment vector, indicating the greatest discriminating ability, but formed a large angle with the average environment axis. ENV3 (Ibadan 2023) had the second longest vector combined with the smallest angle to the AEA. ENV4 (Ikenne 2023) was closely comparable with ENV3. ENV6 (Mokwa 2023) had the second largest angle to the AEA despite moderate discriminating ability. ENV1 (Ibadan 2022) and ENV5 (Kano 2023) had very short vectors and consequently fell within the accession cloud in the biplot; ENV5 additionally recorded the largest angle to the AEA of any environment.

### 3.14 GGE biplot pattern of ‘mean vs. stability’ analysis

The ‘mean vs. stability’ view of the GGE biplot is presented in **Figure 10**. The average environment axis (AEA) is denoted by a single-arrowed line that divides the biplot and indicates the increasing performance of accessions while the high- and low yielding accessions in the biplot are separated by the no-arrow line with accessions on the left section regarded as low yielding and those in the right section as accessions with above average yield. A long projection of an accession to the AEA line (single-arrowed line) indicates a greater contribution to GEI while accessions with a short projection line into the AEA are considered stable. G70 (TPt-270) had the longest positive projection onto the average environment axis, confirming it as the highest-yielding accession with a mean SWPP of 100.24 g, followed by G31 (TPt-198) at 99.87 g, G76 (TPt-280) at 96.62 g and G29 (TPt-187) at 93.51 g. In contrast, G35 (TPt-203) had the longest negative projection with the lowest mean SWPP of 48.43 g, followed by G19 (TPt-174) at 51.97 g and G40 (TPt-21) at 54.18 g. G29 (TPt-187) recorded a very short perpendicular projection from the AEA (0.40). G12 (TPT-16) was similarly stable with an above-average mean SWPP of 82.28 g, and G18 (TPt-173) and G39 (TPt-209) also combined above-average means with short projections. G19 (TPt-174) recorded the shortest projection of all accessions but a low mean SWPP of 51.97 g. G76 (TPt-280), G63 (TPt-26), G52 (TPt-234) and G49 (TPt-223) recorded above-average mean SWPP values but large instability scores.

**Figure 10:**
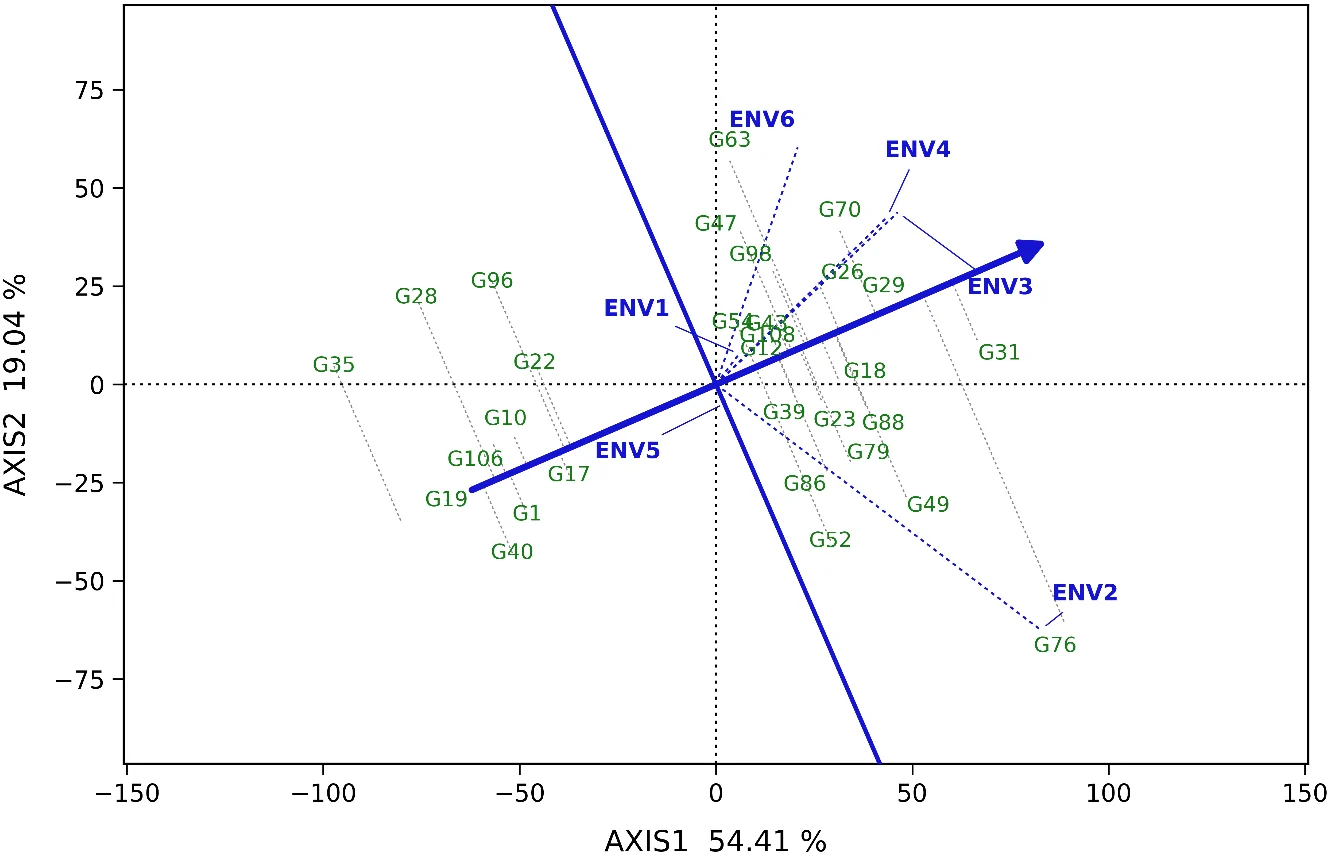
Mean vs. stability view of the GGE biplot based on genotype × environment seed weight per plant data of 30 winged bean accession.

## 4 Discussion

### 4.1 Genetic variability among winged bean accessions

The presence of genetic variability in a crop’s germplasm is crucial for achieving genetic gains in breeding programmes, as it determines the potential for crop improvement through selection and breeding strategies (Hemal and Dodake, 2019). The high variability observed in the yield-related traits suggests that these traits are influenced by both genetic and environmental factors, making them prime targets for breeding programmes aimed at yield improvement (Olanrewaju et al., 2021). By contrast, the low coefficients of variation recorded for the morphological and phenological traits imply that they are more genetically controlled, providing a reliable basis for selection in breeding programmes (Okunlola et al., 2023).

The highly significant differences observed among accessions, environments and their interaction confirm the presence of genetic variability within the germplasm and the influence of the growing environment on trait expression. Similar results were reported by Adegboyega et al. (2021); Tiwari et al. (2022) and Bhadmus et al. (2023), who observed significant differences among accessions, environments and accession × environment interaction in winged bean.

The observation that PCV estimates were only marginally higher than the corresponding GCV values for all traits indicates a limited influence of the environment on trait expression. Total seed weight showed moderate PCV and GCV, implying that sufficient genetic variation exists among the accessions for selection to improve this trait. Traits such as pod weight per plant, number of pods per plant, seed weight per plant, total pod weight, number of seeds per plant and shelling percentage, which combined low GCV with moderate PCV, appear to be more strongly influenced by the environment.

Moderate GAM values indicate the action of both additive and non-additive genes in the control of the traits concerned. High heritability coupled with moderate GAM, as recorded for pod length, days to fifty percent flowering, pod weight and seed weight per pod, implies that selection based on these traits would be effective for their improvement. Similar conclusions were drawn by Adegboyega et al. (2021) for pod weight and seed yield in winged bean germplasm. Comparable heritability estimates for pod length and days to fifty percent flowering were reported by Soni et al. (2022) in winged bean, and high heritability for pod-related traits such as pod length and pod width has also been documented by Adegboyega et al. (2021) and Rakshitha et al. (2025). Contrarily, Hemal and Dodake (2019) reported that pod length showed low heritability.

High heritability combined with low GAM, as recorded for days to fifty percent pod emergence, seed length, seed width, shelling percentage, number of seeds per pod, pod thickness, days to fifty percent mature pod and days to fifty percent dry pod, implies that these traits may not be readily improved through conventional selection. Moderate heritability with moderate GAM, as recorded for total seed weight, number of seeds per plant, total pod weight, seed weight per plant, number of pods per plant and pod weight per plant, implies that although the environment plays a role in the expression of these traits, selection may still be effective in identifying high-performing accessions for use as parental lines. Low heritability and low GAM for pod width indicate that the scope for improving this trait through selection is very limited, which may be attributed to non-additive gene effects. The combination of moderate genotypic and phenotypic coefficients of variation with moderate heritability and GAM observed for total seed weight indicates that this trait can be improved through phenotypic selection. These findings agree with Nandan and Kumar Issar (2009), who reported that seed and pod yield traits in winged bean could be improved through selection.

The range of values recorded for flowering and maturity time was broadly consistent with those reported by Bhadmus et al. (2023) and Soni et al. (2025). The early-flowering accessions TPt-172, TPt-198, TPt-286 and TPt-260 are of particular interest to breeders, since early flowering is associated with early physiological maturity and could be advantageous in short-season or drought-prone production environments (Lepcha et al., 2017). This is of direct relevance to the savannah environments represented in this study, where the crop matured under severe terminal moisture deficit. These accessions are therefore strong candidates as donor parents in crosses aimed at developing early-maturing, high-yielding winged bean varieties for sub-Saharan Africa.

### 4.2 Trait associations and their implications for selection

The dominance of yield-related traits on PC1, which captured the largest proportion of the total phenotypic variation, underscores the biological and agronomic relevance of these characters as primary drivers of genetic diversity in the evaluated germplasm. These traits should therefore be considered priorities for winged bean improvement programmes. Similar results were reported by Bhadmus et al. (2023); Yulianah et al. (2020) and Rakshitha et al. (2025), who found that pod- and seed-related traits were among the most important contributors to PC1.

The contrast between phenology and yield resolved on PC2 indicates that accessions with earlier phenology tended to record higher pod and seed yield. This pattern is consistent with the negative correlations observed between days to fifty percent flowering and the yield traits, and indicates that earliness and high yield are compatible objectives in this germplasm rather than competing ones. This contrasts with Yulianah et al. (2020), who reported that selection for early flowering in Indonesian winged bean accessions could reduce total pod yield, and suggests that the relationship between phenology and yield may differ among germplasm pools.

The clustering of the yield-related traits in a single quadrant of the PCA biplot indicates strong inter-correlations among these traits, which is consistent with the findings of Bhadmus et al. (2023) and Yulianah et al. (2020). Accessions TPt-260, TPt-270, TPt-294, TPt-216 and TPt-286, which clustered close to this group of traits, are characterised by high performance across multiple yield components simultaneously, making them excellent candidates for use as parental lines in winged bean improvement programmes targeting high yield. The broad dispersal of the accessions across the biplot space confirms the substantial genetic diversity present among the evaluated accessions, a finding consistent with that of Bhadmus et al. (2023).

The grouping of the accessions into three clusters indicates the presence of genetically diverse accessions that can be used for cross-breeding. This is consistent with the findings of Bhadmus et al. (2023), who reported that 30 accessions of winged bean were grouped into three major clusters on the basis of 17 agro-morphological traits. Shonde et al. (2023) similarly reported that 15 winged bean accessions were grouped into two clusters on the basis of high yield and maturity traits.

Correlation analysis helps plant breeders to understand the relationship between two traits in terms of both strength and direction, and thereby guides the selection of high-yielding genotypes; traits showing strong and significantly positive correlations with yield can be prioritised during selection (Russinga et al., 2020). The strong positive correlations observed among the phenological traits are consistent with Hansda et al. (2023) and Prasanth et al. (2016), who reported significant positive correlations between days to fifty percent mature pod and days to fifty percent flowering in winged bean genotypes. The significant and positive correlations observed between pod yield-related traits and seed yield-related traits imply that an increase in any of these traits will lead to an increase in pod and seed yield, and these traits should therefore be considered when selecting for improved pod and seed yield in winged bean. Similar findings were reported by Hemal and Dodake (2019); Rakshitha et al. (2025); Sakthivel et al. (2020); Tanzi et al. (2019) and Yulianah et al. (2020), who reported strong positive correlations between pod- and seed-related traits.

The negative correlations recorded between days to fifty percent flowering and the yield traits signify a trade-off between late flowering and yield, such that later-flowering accessions tended to produce fewer pods and lower seed yield. That the genotypic correlations were consistently stronger than their phenotypic counterparts in the phenology traits indicates that this association is genetically based, with environmental variation attenuating its expression at the phenotypic level. This result is in strong agreement with Akeem et al. (2019); Kant and Nandan (2018) and Mohamadali and Madalageri (2004), who reported that days to fifty percent flowering had a significant negative correlation with seed yield per plant in winged bean accessions. The consistency of the trait associations within individual environments further indicates that the relationships reported here are not artefacts of averaging across environments.

### 4.3 Genotype × environment interaction and identification of superior accessions

The large environment main effect implies that SWPP expression in winged bean is strongly modulated by the growing environment, and that selection based on performance at any single site would risk identifying accessions adapted only to the conditions of that site. Evaluation across multiple environments is therefore essential for identifying accessions of broad adaptation. That the accession × environment interaction accounted for a larger share of the variation than the accession main effect indicates that the relative performance of accessions was not fully consistent across environments, and that the identification of broadly adapted accessions requires explicit assessment of stability rather than reliance on mean performance alone. The significant GEI justified the stability analysis undertaken here, as has been reported in similar multi-environment studies in leguminous crops (Pour-Aboughadareh et al., 2022).

A genotype is considered stable when its IPCA 1 score is close to zero, indicating consistent performance regardless of the environment, whereas genotypes with large positive or negative IPCA 1 scores exhibit greater sensitivity to environmental change (Bocianowski et al., 2024). The near-zero IPCA 1 scores of G54 (TPt-238), G108 (TPt-6), G26 (TPt-180) and G12 (TPT-16) indicate that their superior SWPP performance was consistent and largely unaffected by changes in the test environments, making them desirable candidates for further evaluation. For a superior genotype to be widely released and adopted by farmers, its performance must remain consistent across diverse target environments (Abtew et al., 2015; Kondombo et al., 2024). These stable accessions fulfil this criterion and may be advanced to on-farm, sub-regional or regional trials. Several studies have similarly applied the AMMI model to identify stable and high-performing genotypes in leguminous crops under multi-environment conditions (Bomma et al., 2024; Daba et al., 2023; Mwale et al., 2023; Sharma et al., 2025).

G70 (TPt-270) combined the highest mean SWPP with a small IPCA 1 score, indicating that its superior yield was accompanied by comparatively little interaction with the test environments. G31 (TPt-198) followed closely in mean performance, and although its IPCA 1 score indicates somewhat greater sensitivity to environmental conditions, its outstanding yield potential makes it a valuable parental line for crosses targeting high SWPP (Alam et al., 2018). G29 (TPt-187) ranked among both the highest-yielding and the most stable accessions evaluated. G76 (TPt-280) presents a contrasting case: its high mean performance reflects exceptional yield in particular environments rather than consistent performance across the range of conditions tested, and it is better suited to specific than to broad adaptation. Accessions of this type may nonetheless be valuable where the target environment is well defined, or as parents in crosses aimed at combining high yield potential with the stability of accessions such as G54 (TPt-238) and G29 (TPt-187) (Kindie et al., 2022; Olanrewaju et al., 2021).

The proportion of variation explained by the first two principal components of the GGE biplot indicates that the biplot provides an adequate two-dimensional representation of genotypic performance and stability for SWPP across the six test environments (Hagos and Abay, 2013; Mullualem et al., 2024). The ideal genotype, represented by the centre of the innermost concentric circle, signifies a genotype combining the highest possible mean SWPP with the greatest stability across all test environments (Khan et al., 2021; Linus et al., 2023). For effective selection in multi-environment trials, an ideal genotype must possess both high mean performance and broad stability (Kindie et al., 2022; Lee et al., 2023). G29 (TPt-187), G31 (TPt-198) and G70 (TPt-270) fulfil both criteria and are therefore strong candidates for release as improved varieties or for use as elite parental lines in crossing programmes aimed at developing high-yielding and broadly adapted winged bean varieties. Similar findings have been reported in related legume crops, where the GGE biplot ideal genotype view successfully identified superior and stable genotypes suitable for multi-environment production and further genetic advancement (Kebede et al., 2025; Linus et al., 2023; Olanrewaju et al., 2021; Sansa et al., 2025).

An ideal test environment is defined by a long vector length, indicating high discriminating ability, and a small angle to the average environment axis, indicating high representativeness of the overall test conditions (Ghaffari et al., 2021; Liu et al., 2026). The use of ideal environments in multi-environment trial design has been widely advocated as a cost-effective strategy for optimising genotype evaluation and accelerating genetic gains (Gumede et al., 2022; Kindie et al., 2022; Singh et al., 2022). The near-coincidence of ENV3 and ENV4 in the biplot indicates that these environments ranked the accessions in an almost identical manner despite differing in seasonal rainfall by 251 mm and representing two distinct agroecological zones, and their combination of high discriminating power and representativeness makes them the most suitable environments for evaluating and selecting superior winged bean accessions. Although ENV2 was the most discriminating environment overall, its large angle to the AEA indicates that selection conducted there alone would favour accessions adapted to its particular conditions rather than those of broad adaptation. The very short vectors of ENV1 and ENV5 indicate that neither environment discriminated effectively among the accessions, limiting their suitability as test environments for broad adaptation studies (Ghaffari et al., 2021; Liu et al., 2026). Similar findings have been reported in underutilised legumes, where environments combining high discriminating ability with small angles to the AEA were recommended as superior for future genotype selection trials (Esan et al., 2023; Linus et al., 2023). ENV3 and ENV4 are therefore the most recommended environments for future winged bean multi-environment evaluation trials targeting SWPP (Linus et al., 2023; Tiwari et al., 2019).

The polygon view of the ‘which-won-where’ biplot is extensively used to visualise genotype × environment interactions, and assists in identifying crossover interactions, differentiating mega-environments and determining specific adaptations of genotypes (Olanrewaju et al., 2021). The distribution of the six environments across four sectors indicates the presence of crossover genotype × environment interaction for SWPP (Hagos and Abay, 2013; Khan et al., 2021; Mullualem et al., 2024). Notably, ENV3 and ENV4, the two environments closest to the ideal test environment, fell within the same sector, in which G31 (TPt-198) was the vertex accession. This co-location is agronomically important: although the six environments as a whole were divided among four sectors, the two that were both most discriminating and most representative of the target region behaved as a single unit. Selection conducted at either site would therefore yield the same outcome, and the crossover interaction detected across the full set of environments is driven largely by the atypical conditions at ENV2, ENV5 and ENV6 rather than by the conditions under which the crop would normally be evaluated. It should nonetheless be emphasised that sector membership is derived from the two-dimensional approximation of the biplot and does not always coincide with the highest observed mean in a given environment; the polygon view should therefore be interpreted as delineating the broad structure of the interaction rather than as an exact ranking within individual environments. The vertex genotypes occupying sectors containing no environments were the poorest performers across all test environments and are not recommended for use in SWPP improvement programmes. Which-won-where patterns identifying distinct winning genotypes and mega-environment structures have been reported in related leguminous crops evaluated across multi-environment trials (Abebe et al., 2024; Haile and Banteayehu, 2025; Katuuramu et al., 2020; Parihar et al., 2024; Sokolović et al., 2024).

In the mean versus stability view, the length of the projection onto the AEA ordinate determines the stability of a genotype; the longer the projection, the more unstable the genotype (Tiwari et al., 2022). G29 (TPt-187) combined high mean SWPP with outstanding stability, making it the most outstanding accession identified in this study, while G12 (TPT-16), G18 (TPt-173) and G39 (TPt-209) also combined above-average means with short projections, confirming their value as broadly adapted accessions for SWPP improvement (Esan et al., 2023; Olanrewaju et al., 2021). It should be noted that G19 (TPt-174) recorded the shortest projection of all accessions but a low mean SWPP; consistently low performance is not a desirable form of stability, and this accession is not recommended for selection. By contrast, G76 (TPt-280), G63 (TPt-26), G52 (TPt-234) and G49 (TPt-223) recorded above-average mean SWPP values but exhibited large instability scores, indicating strong genotype × environment interaction and environment-specific adaptation. These accessions may still be useful for environment-specific breeding but are not recommended for broad adaptation across the range of growing conditions represented in this study (Khan et al., 2021; Olanrewaju et al., 2021). The stability analysis of yield complements the genotype ranking analysis in determining the adaptability and stability of genotypes based on their agronomic performance across the test locations. Similar patterns of mean versus stability differentiation using GGE biplot analysis have been reported in other underutilised legumes, consistently demonstrating that genotypes combining above-average yield with short AEA perpendicular projections are the most suitable for recommendation in multi-environment breeding programmes (Abebe et al., 2024; Esan et al., 2023; Haile and Banteayehu, 2025; Katuuramu et al., 2020; Khan et al., 2021; Mekonnen et al., 2022; Parihar et al., 2024; Sokolović et al., 2024; Tiwari et al., 2022).

### 4.4 Influence of climatic and edaphic conditions on accession stability and test environment quality

The six test environments differed markedly in both climatic and edaphic characteristics (**Tables 1**, **2**), and these contrasts help to explain the patterns of stability and test environment quality resolved by the AMMI and GGE analyses. ENV4 (Ikenne 2023) combined abundant and well-distributed rainfall (1208 mm), the narrowest diurnal temperature range of any environment (23.1–29.2 °C), the highest relative humidity (82%) and adequate available phosphorus (31.03 mg kg^-1^), representing near-optimum climatic and edaphic conditions. ENV2 (Ikenne 2022) presented a closely comparable climatic profile, differing from ENV4 by only 71 mm in seasonal rainfall and 0.8 °C in mean temperature. The consistency of the Ikenne site across two seasons supports its recommendation as an evaluation site for winged bean, although ENV2 combined the greatest discriminating ability of the six environments with a large angle to the average environment axis, indicating that selection conducted there alone would favour accessions adapted to its particular conditions.

By contrast, ENV5 (Kano 2023) represented the climatic extremes of the study. Kano imposed a combined heat and moisture-deficit stress (mean maximum temperature 35.5 °C, 294 mm seasonal rainfall of which only 16.7 mm fell after September, and mean relative humidity of 52%) on a coarse-textured soil of very low organic matter (0.03%) and total nitrogen (0.02%). Temperatures in this range exceed the optimum for reproductive development in winged bean and are associated with flower abscission and reduced pod set, while the near-absence of late-season rainfall imposed severe terminal drought during pod filling. These conditions are consistent with the mean SWPP of only 7.06 g recorded in this environment, and with its very short environment vector, since severe stress of this kind suppresses the expression of genotypic differences.

Mokwa (ENV6), while climatically less severe, was the most nutrient-limited site, with very low organic matter (0.12%) and available phosphorus (8.00 mg kg^-1^), and received only 87 mm of rainfall after September. Because winged bean derives much of its nitrogen through symbiotic fixation, low soil nitrogen is unlikely to have been the primary constraint at these sites; phosphorus availability, which directly limits nodulation and pod filling in tropical legumes, is the more probable edaphic driver of the reduced yields observed. The combination of moderate terminal water deficit with phosphorus limitation distinguishes ENV6 from the other environments and is consistent with its large angle to the average environment axis (45.41°), which identifies it as the least representative of the test conditions despite its moderate discriminating ability.

ENV3 (Ibadan 2023) recorded the second longest environment vector and the smallest angle to the average environment axis, giving it the strongest overall combination of discriminating ability and representativeness among the six environments. The Ibadan site recorded the highest available phosphorus of the study (36.42 mg kg^-1^), and the 2023 season brought exceptionally high late-season rainfall, including 311 mm in October alone. The absence of both nutritional and terminal moisture constraints allowed fuller expression of genotypic differences and hence high discriminating power. These conditions were nonetheless among the most favourable encountered in the study, and selections made exclusively in this environment would risk favouring accessions adapted to unusually favourable late-season conditions.

These stress gradients inform the performance of the superior accessions. G31 (TPt-198) maintained high SWPP across the full range of conditions examined and recorded the highest observed value at ENV6 (124.34 g), the phosphorus- and organic-matter-limited site, indicating tolerance of nutrient-poor Southern Guinea savannah conditions. G70 (TPt-270) recorded the highest overall mean SWPP (100.24 g) and the highest observed value at ENV1 (98.00 g), while G29 (TPt-187) recorded the highest observed value at ENV3 (167.13 g) and combined a high mean (93.51 g) with the smallest deviation from the average environment axis of any high-yielding accession. These three accessions maintained competitive seed yield across both the favourable humid-forest environments and the nutrient-poor Southern Guinea savannah and heat-stressed Sudan savannah environments. This combination of high mean performance with minimal environmental sensitivity marks G31, G70 and G29 as the accessions best suited to deployment across the low-input production systems that predominate in the target region.

These associations should be interpreted as descriptive rather than causal, since the climatic and edaphic variables were characterised but not experimentally manipulated, and the environments differed simultaneously in several respects. Targeted screening of the identified accessions under controlled water deficit, controlled phosphorus limitation and controlled high-temperature regimes would be required to confirm the physiological basis of the adaptation patterns inferred here.

## 5 Conclusion

From this study, accessions TPt-172, TPt-198, TPt-286 and TPt-260 were considered early flowering accessions. The rank-sum index applied to seed weight per plant (SWPP) performance across environments identified best-performing accessions from the 110 evaluated, which were subsequently subjected to stability analysis. The environment main effect accounted for the largest share of the total variation in SWPP, and the accession × environment interaction exceeded the accession main effect, confirming that mean performance alone is an insufficient basis for recommendation in this crop and that formal stability assessment is required. AMMI and GGE biplot analyses consistently identified TPt-270 (G70) as the highest-yielding accession, closely followed by TPt-198 (G31), while TPt-187 (G29) was identified as the accession closest to the ideal genotype, combining a high mean SWPP with the greatest stability across the six test environments. TPt-198 was additionally ranked among the early-flowering accessions, making it particularly valuable for the development of early-maturing, high-yielding varieties. Together, TPt-270, TPt-198 and TPt-187 represent the most promising accessions identified in this study and are recommended as elite parental lines for winged bean improvement programmes targeting superior seed yield.

The GGE biplot analysis of the ideal environment identified ENV3 (Ibadan 2023) and ENV4 (Ikenne 2023) as the most suitable locations for future multi-environment evaluation trials targeting SWPP in winged bean, combining the strongest balance of discriminating power and representativeness among the six environments tested. These two environments ranked the accessions in an almost identical manner despite representing distinct agroecological zones, indicating that either would provide reliable information for selection. Future evaluation trials should therefore prioritise the Ibadan and Ikenne sites as primary screening environments. In contrast, the Kano environment discriminated poorly among accessions under the severe terminal drought experienced in 2023 and is not recommended as a primary selection site, although evaluation under such conditions remains valuable for assessing adaptation to marginal environments. Although the trials were conducted in Nigeria, the six environments spanned a 4.4-fold range in seasonal rainfall and four agroecological zones from humid forest to Sudan savannah, encompassing much of the climatic and edaphic variation under which winged bean would be cultivated across West and Central Africa. The accessions identified as broadly adapted across this gradient are therefore expected to perform reliably in comparable agroecologies elsewhere in sub-Saharan Africa, although confirmation through multi-country evaluation remains necessary.

Expanding this study through field evaluations in additional agro-ecological zones across Nigeria and other winged bean-growing regions in sub-Saharan Africa and Asia would further validate the stability and broad adaptation of the identified superior accessions, supporting their integration into national and regional winged bean improvement programmes. Such expanded evaluations would also generate the multi-location performance data necessary for formal variety release and recommendation. This work confirms that winged bean germplasm harbours significant untapped genetic potential, and that systematic exploitation of this diversity through intensive and accurate selection and crossing strategies can substantially accelerate genetic gains in this neglected and underutilised legume, thereby contributing to improved food and nutritional security among smallholder farming communities across sub-Saharan Africa.

## Data Availability

The raw data supporting the conclusions of this article will be made available by the authors, without undue reservation.

## Supplementary material

Supplementary Table 1 Monthly rainfall, temperature and relative humidity recorded over the cropping period (July–December) at the four experimental locations during the 2022 and 2023 seasons.

Supplementary Table 2 Agronomic and yield traits, with their mode of collection, observation and method of evaluation used for this study.

Supplementary Table 3 Best 20 and poorest 10 performing accessions based on seed yield used for stability analysis.

Supplementary Table 4 Summary statistics of the traits scored.

Supplementary Table 5 Means and summary statistics of agro-morphological traits of the top 20 winged bean accessions based on total pod weight.

Supplementary Table 6 Mean performance based on the Duncan Multiple Range Test of agro-morphological traits of the top 20 winged bean accession based on total pod weight.

Supplementary Table 7 Principal component scores of the 110 winged bean accessions for the first five principal components, ranked by PC1 based on accessions.

Supplementary Table 8 Grouping of accessions in different clusters based on flowering, pod and seed yield.

Supplementary Table 9 Phenotypic correlation coefficients among 21 agronomic and yield-related traits of 110 winged bean accessions evaluated across six environments in Nigeria.

Supplementary Table 10 Phenotypic correlation coefficients among agronomic and yield-related traits of 110 winged bean accessions, computed separately within each of the six test environments.

Supplementary Table 11 Sensitivity analysis of AMMI and GGE biplot results for seed weight per plant (SWPP) of 30 winged bean accessions: comparison of the full six-environment dataset with the balanced subset of four environments evaluated in 2023.

Supplementary Figure 1 Classification of 21 agronomic and yield-related traits of 110 winged bean accessions by genetic advance as a percentage of the mean at 5% selection intensity.

Supplementary Figure 2 Broad-sense heritability estimates for 21 agronomic and yield-related traits of 110 winged bean accessions evaluated across six environments in Nigeria.

Supplementary Figure 3 Scree plot diagram of Principal Components of agronomic and yield traits of 110 accessions of Winged bean accessions.

Supplementary Figure 4 Bar chart showing the mean of yield and agronomic traits for each cluster in the winged bean accessions studied.
