# Supplementary material for: Multi-environment evaluation identifies high-yielding and stable winged bean (Psophocarpus tetragonolobus (L.) DC.) accessions for sub-Saharan Africa
Source: Front Plant Sci. 2026 Sep 30;17:1916275. doi: 10.3389/fpls.2026.1916275 (PMC13662050; doi:10.3389/fpls.2026.1916275)
Supplement: Supplementary file 1 [file DataSheet1.docx]

**Supplementary Table S1. Monthly rainfall, temperature and relative humidity recorded over the cropping period (July–December) at the four experimental locations during the 2022 and 2023 seasons.**

Data were obtained from the IITA GIS Unit. Trials were sown in early July in all environments. Environment codes ENV1 to ENV6 correspond to the six location–year combinations evaluated in this study; Kano and Mokwa were evaluated in 2023 only, and the 2022 series for these two locations is provided for reference. Rainfall is reported as the monthly total and, in the final row of each block, as the cumulative total over the cropping period; temperature and relative humidity are monthly means, and the final row gives the mean over the cropping period.

**Ibadan 2022 (ENV1)**

| **Month** | **Rainfall (mm)** | **Temperature (°C)** | | | **Relative humidity (%)** | | |
| --- | --- | --- | --- | --- | --- | --- | --- |
|  |  | **Min.** | **Max.** | **Mean** | **Min.** | **Max.** | **Mean** |
| July | 124.3 | 22.4 | 27.4 | 24.9 | 68.0 | 93.0 | 80.5 |
| August | 169.4 | 21.3 | 26.3 | 23.8 | 73.0 | 94.0 | 83.5 |
| September | 231.5 | 22.3 | 27.0 | 24.6 | 69.0 | 94.0 | 81.5 |
| October | 156.9 | 22.3 | 29.0 | 25.6 | 60.0 | 95.0 | 77.5 |
| November | 16.2 | 23.1 | 32.1 | 27.6 | 30.0 | 96.0 | 63.0 |
| December | 0.0 | 21.2 | 32.9 | 27.0 | 21.0 | 88.0 | 54.5 |
| **Total / mean** | **698.3** | **22.1** | **29.1** | **25.6** | **53.5** | **93.3** | **73.4** |

**Ikenne 2022 (ENV2)**

| **Month** | **Rainfall (mm)** | **Temperature (°C)** | | | **Relative humidity (%)** | | |
| --- | --- | --- | --- | --- | --- | --- | --- |
|  |  | **Min.** | **Max.** | **Mean** | **Min.** | **Max.** | **Mean** |
| July | 320.2 | 22.4 | 27.8 | 25.1 | 68.5 | 100.0 | 84.2 |
| August | 226.4 | 21.7 | 27.6 | 24.6 | 66.6 | 100.0 | 83.3 |
| September | 402.3 | 22.5 | 27.9 | 25.2 | 73.0 | 98.9 | 86.0 |
| October | 260.2 | 23.1 | 29.0 | 26.1 | 63.2 | 99.4 | 81.3 |
| November | 40.0 | 22.8 | 29.7 | 26.2 | 52.7 | 100.0 | 76.3 |
| December | 29.4 | 20.6 | 29.1 | 24.9 | 44.1 | 100.0 | 72.0 |
| **Total / mean** | **1278.5** | **22.2** | **28.5** | **25.4** | **61.4** | **99.7** | **80.5** |

**Ibadan 2023 (ENV3)**

| **Month** | **Rainfall (mm)** | **Temperature (°C)** | | | **Relative humidity (%)** | | |
| --- | --- | --- | --- | --- | --- | --- | --- |
|  |  | **Min.** | **Max.** | **Mean** | **Min.** | **Max.** | **Mean** |
| July | 288.5 | 23.1 | 28.7 | 25.9 | 70.0 | 98.0 | 84.0 |
| August | 99.1 | 22.9 | 29.4 | 26.1 | 74.0 | 98.0 | 86.0 |
| September | 251.0 | 22.7 | 28.2 | 25.4 | 70.0 | 96.0 | 83.0 |
| October | 310.8 | 23.0 | 30.0 | 26.5 | 64.0 | 98.0 | 81.0 |
| November | 6.9 | 23.8 | 31.3 | 27.6 | 48.0 | 97.0 | 72.5 |
| December | 0.0 | 21.8 | 33.4 | 27.6 | 15.0 | 88.0 | 51.5 |
| **Total / mean** | **956.3** | **22.9** | **30.2** | **26.5** | **56.8** | **95.8** | **76.3** |

**Ikenne 2023 (ENV4)**

| **Month** | **Rainfall (mm)** | **Temperature (°C)** | | | **Relative humidity (%)** | | |
| --- | --- | --- | --- | --- | --- | --- | --- |
|  |  | **Min.** | **Max.** | **Mean** | **Min.** | **Max.** | **Mean** |
| July | 303.1 | 23.4 | 28.8 | 26.1 | 67.6 | 100.0 | 83.8 |
| August | 188.8 | 23.1 | 28.5 | 25.8 | 67.8 | 99.4 | 83.6 |
| September | 356.3 | 23.5 | 28.6 | 26.1 | 69.4 | 99.2 | 84.3 |
| October | 225.9 | 23.8 | 29.1 | 26.5 | 67.2 | 99.5 | 83.3 |
| November | 131.7 | 23.8 | 29.4 | 26.6 | 66.3 | 100.0 | 83.2 |
| December | 1.8 | 21.0 | 30.7 | 25.9 | 46.2 | 100.0 | 73.1 |
| **Total / mean** | **1207.6** | **23.1** | **29.2** | **26.1** | **64.1** | **99.7** | **81.9** |

**Kano 2023 (ENV5)**

| **Month** | **Rainfall (mm)** | **Temperature (°C)** | | | **Relative humidity (%)** | | |
| --- | --- | --- | --- | --- | --- | --- | --- |
|  |  | **Min.** | **Max.** | **Mean** | **Min.** | **Max.** | **Mean** |
| July | 86.9 | 24.3 | 37.4 | 30.9 | 21.1 | 95.2 | 58.2 |
| August | 81.6 | 23.2 | 34.8 | 29.0 | 26.1 | 98.5 | 62.3 |
| September | 108.6 | 23.2 | 36.1 | 29.6 | 20.4 | 97.7 | 59.0 |
| October | 16.7 | 20.9 | 36.6 | 28.8 | 9.7 | 97.2 | 53.5 |
| November | 0.0 | 19.7 | 35.8 | 27.8 | 10.5 | 83.0 | 46.8 |
| December | 0.0 | 13.3 | 32.2 | 22.8 | 8.2 | 59.4 | 33.8 |
| **Total / mean** | **293.8** | **20.8** | **35.5** | **28.1** | **16.0** | **88.5** | **52.2** |

**Mokwa 2023 (ENV6)**

| **Month** | **Rainfall (mm)** | **Temperature (°C)** | | | **Relative humidity (%)** | | |
| --- | --- | --- | --- | --- | --- | --- | --- |
|  |  | **Min.** | **Max.** | **Mean** | **Min.** | **Max.** | **Mean** |
| July | 196.9 | 23.6 | 30.4 | 27.0 | 55.9 | 99.3 | 77.6 |
| August | 188.1 | 23.3 | 29.7 | 26.5 | 60.5 | 98.8 | 79.7 |
| September | 126.4 | 23.3 | 30.2 | 26.8 | 59.6 | 100.0 | 79.8 |
| October | 66.7 | 23.5 | 31.8 | 27.6 | 36.1 | 100.0 | 68.0 |
| November | 20.3 | 22.9 | 34.0 | 28.4 | 20.3 | 100.0 | 60.1 |
| December | 0.0 | 17.8 | 35.6 | 26.7 | 14.8 | 77.7 | 46.2 |
| **Total / mean** | **598.4** | **22.4** | **31.9** | **27.2** | **41.2** | **96.0** | **68.6** |

**Mokwa 2022 (not a test environment)**

| **Month** | **Rainfall (mm)** | **Temperature (°C)** | | | **Relative humidity (%)** | | |
| --- | --- | --- | --- | --- | --- | --- | --- |
|  |  | **Min.** | **Max.** | **Mean** | **Min.** | **Max.** | **Mean** |
| July | 86.7 | 23.0 | 30.6 | 26.8 | 49.2 | 100.0 | 74.6 |
| August | 158.6 | 22.5 | 29.6 | 26.1 | 53.7 | 98.2 | 76.0 |
| September | 194.0 | 22.6 | 29.5 | 26.1 | 60.9 | 100.0 | 80.5 |
| October | 141.7 | 21.7 | 30.8 | 26.2 | 38.1 | 100.0 | 69.0 |
| November | 0.3 | 18.7 | 33.7 | 26.2 | 22.2 | 97.2 | 59.7 |
| December | 0.4 | 16.3 | 32.8 | 24.5 | 18.8 | 92.8 | 55.8 |
| **Total / mean** | **581.7** | **20.8** | **31.2** | **26.0** | **40.5** | **98.0** | **69.3** |

**Kano 2022 (not a test environment)**

| **Month** | **Rainfall (mm)** | **Temperature (°C)** | | | **Relative humidity (%)** | | |
| --- | --- | --- | --- | --- | --- | --- | --- |
|  |  | **Min.** | **Max.** | **Mean** | **Min.** | **Max.** | **Mean** |
| July | 126.3 | 22.1 | 32.5 | 27.3 | 30.6 | 99.1 | 64.8 |
| August | 180.6 | 21.5 | 30.7 | 26.1 | 34.7 | 100.0 | 67.3 |
| September | 107.8 | 21.6 | 32.3 | 26.9 | 33.5 | 100.0 | 66.8 |
| October | 16.8 | 19.2 | 33.3 | 26.2 | 18.6 | 100.0 | 59.3 |
| November | 0.0 | 13.9 | 32.1 | 23.0 | 13.2 | 79.8 | 46.5 |
| December | 0.0 | 12.4 | 29.7 | 21.1 | 11.1 | 71.4 | 41.2 |
| **Total / mean** | **431.5** | **18.4** | **31.8** | **25.1** | **23.6** | **91.7** | **57.7** |

**Supplementary Table S2: Agronomic and yield traits, with their mode of collection, observation and method of evaluation used for this study.**

| **S/N** | **Traits** | **Mode of Observation/method of evaluation** |
| --- | --- | --- |
| 1 | 50% flower (FPF) | Number of days from sowing to 50% flower opening per plot |
| 2 | 50% pod emergence (FPP) | Number of days from sowing to 50% emergence of pod per plot |
| 3 | 50% mature pod (FPMP) | Number of days from sowing to 50% physiological matured pods |
| 4 | 50% dry pod (FPDP) | Number of days from sowing to 50% dry pods |
| 5 | Number of seeds per pod (NSPPD) | Mean number of seeds in a pod |
| 6 | Seed weight per pod (SWPPD) (g) | Mean weight of seeds in a pod |
| 7 | Number of seeds per plant (NSPP) | Mean number of seeds per plot |
| 8 | Seed length (SL) (mm) | Mean length of seeds per plot |
| 9 | Seed weight per plant (SWPP) (g) | Mean weight of seeds per plot |
| 10 | Seed width (SWT) (mm) | Mean width of seeds per plot |
| 11 | Seed thickness (ST) (mm) | Mean thickness of seeds per plot |
| 12 | Pod length (PL) (cm) | Mean length of dry pods per individual plant |
| 13 | Number of pods per plant (NPPP) | Mean number of pods per plot |
| 14 | Pod weight per plant (PWPP) (g) | Mean weight of dry pods per plot |
| 15 | Pod weight (PW) (g) | Mean weight of dry pods per individual plant |
| 16 | Pod width (PWT) (mm) | Mean width of dry pods per individual plant |
| 17 | Pod Thickness (PT) (mm) | Mean pod thickness per individual plant |
| 18 | Total pod weight (TPDW) (g) | Total number of pods harvested per individual plant and weighed |
| 19 | Total seed weight (TSW) (g) | Total number of seeds harvested per individual plant and weighed |
| 20 | 100 seed weight (HSW) (g) | Weight of a hundred seeds |
| 21 | Shelling percentage | The ratio in percentage of the weight of seeds to the weight of pods |

**Supplementary Table S3: Best 20 and poorest 10 performing accessions based on seed yield used for stability analysis.**

| **RANK** | **GROUP** | **ACCESSION** | **ACCESSION CODE** |
| --- | --- | --- | --- |
| 1 | Best | TPt-198 | G31 |
| 2 | Best | TPt-286 | G79 |
| 3 | Best | TPt-209 | G39 |
| 4 | Best | TPt-173 | G18 |
| 5 | Best | TPt-178 | G23 |
| 6 | Best | TPt-180 | G26 |
| 7 | Best | TPt-187 | G29 |
| 8 | Best | TPt-280 | G76 |
| 9 | Best | TPt-234 | G52 |
| 10 | Best | TPT-16 | G12 |
| 11 | Best | TPt-22 | G47 |
| 12 | Best | TPt-223 | G49 |
| 13 | Best | TPt-26 | G63 |
| 14 | Best | TPt-238 | G54 |
| 15 | Best | TPt-213 | G43 |
| 16 | Best | TPt-270 | G70 |
| 17 | Best | TPt-292 | G86 |
| 18 | Best | TPt-294 | G88 |
| 19 | Best | TPt-6 | G108 |
| 20 | Best | TPt-33 | G98 |
| 21 | Poorest | TPt-153 | G10 |
| 22 | Poorest | TPt-172 | G17 |
| 23 | Poorest | TPt-174 | G19 |
| 24 | Poorest | TPt-182 | G28 |
| 25 | Poorest | TPt-203 | G35 |
| 26 | Poorest | TPt-21 | G40 |
| 27 | Poorest | TPt-51 | G106 |
| 28 | Poorest | TPt-16W | G1 |
| 29 | Poorest | TPt-177 | G22 |
| 30 | Poorest | TPt-31 | G96 |

**Supplementary Table S4: Summary statistics of the traits scored.**

| **Traits** | **Min** | **Max** | **Range** | **Mean** | **StdDev** | **CV** | **StdError** |
| --- | --- | --- | --- | --- | --- | --- | --- |
| FPF | 50.0 | 111.0 | 61.0 | 75.1 | 12.5 | 0.2 | 0.3 |
| FPP | 57.0 | 119.0 | 62.0 | 85.2 | 11.6 | 0.1 | 0.3 |
| FPMP | 77.0 | 156.0 | 79.0 | 117.6 | 14.4 | 0.1 | 0.3 |
| FPDP | 106.0 | 164.0 | 58.0 | 134.3 | 11.0 | 0.1 | 0.3 |
| NPPP | 1.0 | 76.3 | 75.3 | 30.2 | 16.2 | 0.5 | 0.4 |
| PWPP | 1.1 | 489.6 | 488.5 | 173.2 | 105.4 | 0.6 | 2.6 |
| PW | 1.7 | 11.9 | 10.3 | 6.8 | 2.0 | 0.3 | 0.0 |
| PL | 11.0 | 28.1 | 17.1 | 19.6 | 3.3 | 0.2 | 0.1 |
| PWT | 7.7 | 13.6 | 5.9 | 10.6 | 1.1 | 0.1 | 0.0 |
| PT | 6.8 | 12.3 | 5.5 | 9.5 | 1.0 | 0.1 | 0.0 |
| NSPPD | 5.6 | 16.4 | 10.8 | 11.1 | 1.9 | 0.2 | 0.0 |
| SWPPD | 0.6 | 6.0 | 5.4 | 3.3 | 1.0 | 0.3 | 0.0 |
| SL | 6.9 | 10.4 | 3.5 | 8.7 | 0.6 | 0.1 | 0.0 |
| SWT | 6.7 | 9.3 | 2.6 | 8.0 | 0.5 | 0.1 | 0.0 |
| ST | 5.3 | 8.6 | 3.3 | 7.0 | 0.6 | 0.1 | 0.0 |
| NSPP | 1.0 | 753.4 | 752.4 | 268.8 | 159.6 | 0.6 | 3.9 |
| SWPP | 0.5 | 243.7 | 243.2 | 80.2 | 51.7 | 0.6 | 1.3 |
| HSW | 12.6 | 48.8 | 36.2 | 30.6 | 6.4 | 0.2 | 0.2 |
| TSW | 0.0 | 1618.1 | 1618.1 | 488.4 | 370.8 | 0.8 | 9.0 |
| TPDW | 1.1 | 3423.2 | 3422.1 | 1042.0 | 742.5 | 0.7 | 18.0 |
| SHP | 25.8 | 64.1 | 38.3 | 45.2 | 6.8 | 0.1 | 0.2 |

*Min: Minimum, Max: Maximum, StdDev: Standard Deviation, CV: coefficient of Variation, StdError: Standard Error.*

**Supplementary Table S5: Means and summary statistics of agro-morphological traits of the top 20 winged bean accessions based on total pod weight.**

| **ACCESSIONS** | | **FPF** | **FPP** | **FPMP** | **FPDP** | **NPPP** | **PWPP** | **PW** | **PL** | **PWT** | **PT** |
| --- | --- | --- | --- | --- | --- | --- | --- | --- | --- | --- | --- |
|  | **TPt-270** | 74.57 | 82.43 | 117.87 | 132.43 | 35.59 | 233.05 | 7.93 | 21.75 | 10.99 | 9.44 |
|  | **TPt-294** | 69.56 | 83.20 | 120.19 | 134.88 | 36.51 | 233.01 | 7.83 | 21.55 | 11.43 | 9.24 |
|  | **TPt-216** | 70.13 | 83.25 | 117.75 | 134.40 | 38.72 | 231.66 | 8.32 | 21.89 | 10.40 | 9.66 |
|  | **TPt-260** | 68.33 | 79.07 | 115.67 | 131.36 | 38.13 | 227.98 | 8.23 | 21.88 | 10.92 | 9.13 |
|  | **TPt-187** | 72.28 | 82.22 | 111.41 | 130.60 | 38.34 | 218.34 | 7.25 | 19.81 | 10.51 | 9.19 |
|  | **TPt-198** | 67.56 | 79.41 | 115.00 | 129.20 | 34.43 | 215.33 | 6.68 | 18.87 | 10.12 | 9.73 |
|  | **TPt-215** | 73.46 | 84.25 | 118.54 | 136.42 | 35.95 | 213.94 | 7.52 | 19.38 | 10.43 | 9.82 |
|  | **TPt-4-A** | 70.63 | 81.38 | 118.63 | 129.07 | 35.02 | 213.80 | 7.07 | 19.39 | 10.59 | 9.87 |
|  | **TPt-180** | 82.88 | 89.40 | 122.38 | 135.60 | 36.64 | 212.96 | 7.18 | 22.15 | 10.49 | 9.85 |
|  | **TPt-206** | 73.47 | 83.71 | 115.94 | 131.92 | 38.39 | 209.03 | 6.48 | 18.67 | 10.85 | 9.87 |
|  | **TPt-26** | 72.36 | 84.07 | 118.93 | 133.64 | 32.78 | 202.06 | 7.21 | 20.04 | 10.33 | 9.72 |
|  | **TPt-280** | 79.59 | 88.63 | 121.18 | 135.00 | 28.68 | 201.79 | 8.26 | 22.83 | 10.97 | 9.10 |
|  | **TPt-205** | 71.67 | 84.29 | 117.61 | 137.76 | 26.45 | 201.69 | 7.12 | 19.27 | 11.44 | 10.12 |
|  | **TPt-33** | 81.11 | 89.12 | 121.06 | 138.28 | 37.33 | 201.12 | 6.77 | 19.59 | 11.17 | 9.64 |
|  | **TPt-286** | 65.71 | 79.35 | 115.59 | 135.31 | 31.35 | 200.87 | 7.90 | 20.38 | 10.72 | 9.71 |
|  | **TPt-291** | 74.27 | 83.40 | 116.75 | 135.67 | 27.70 | 198.26 | 8.16 | 22.95 | 11.42 | 9.70 |
|  | **TPt-223** | 77.13 | 86.69 | 120.81 | 137.27 | 32.76 | 197.87 | 6.55 | 19.64 | 9.99 | 9.14 |
|  | **TPt-292** | 73.06 | 83.19 | 115.24 | 131.80 | 33.66 | 197.61 | 7.53 | 21.06 | 10.77 | 9.44 |
|  | **TPt-178** | 81.35 | 87.67 | 121.38 | 133.23 | 30.11 | 195.01 | 7.29 | 20.61 | 10.91 | 9.28 |
|  | **TPt-288** | 70.27 | 79.50 | 112.50 | 132.85 | 30.72 | 194.29 | 6.85 | 21.30 | 10.67 | 9.42 |
|  | **MIN** | 63.47 | 73.65 | 105.25 | 124.92 | 22.65 | 113.52 | 5.14 | 16.41 | 9.98 | 8.33 |
|  | **MAX** | 90.80 | 98.27 | 130.82 | 144.75 | 39.11 | 233.05 | 8.32 | 23.70 | 11.44 | 10.39 |
|  | **MEAN** | 75.26 | 85.29 | 117.61 | 134.37 | 30.28 | 173.66 | 6.83 | 19.61 | 10.60 | 9.48 |
|  | **SEM** | 0.50 | 0.42 | 0.41 | 0.35 | 0.37 | 2.34 | 0.07 | 0.14 | 0.03 | 0.03 |
|  | **STDEV** | 5.22 | 4.42 | 4.27 | 3.65 | 3.84 | 24.51 | 0.69 | 1.46 | 0.35 | 0.36 |
|  | **CV (%)** | 6.93 | 5.18 | 3.63 | 2.72 | 12.69 | 14.11 | 10.08 | 7.44 | 3.26 | 3.79 |

*Note: Fifty Percent Flower (FPF); Fifty Percent Pod emergence (FPP); Fifty Percent Mature Pod (FPMP); Fifty Percent Dry Pod (FPDP); Number of Pod Per Plant (NPPP); Pod weight per plant (PWPP); Pod Weight (PW); Pod Length (PL); Pod Width (PWT); Number of seeds Per Pod (NSPPD).*

**Supplementary Table S5 cont’d: Means and summary statistics agro-morphological traits of the top 20 winged bean accession based on total pod weight.**

| **ACCESSIONS** |  | **NSPPD** | **SWPPD** | **SL** | **SWT** | **ST** | **NSPP** | **SWPP** | **HSW** | **TSW** | **TPDW** | **SHP** |
| --- | --- | --- | --- | --- | --- | --- | --- | --- | --- | --- | --- | --- |
| TPt-270 |  | 11.99 | 3.90 | 8.87 | 8.14 | 7.01 | 334.51 | 113.94 | 34.45 | 735.77 | 1507.89 | 46.77 |
| TPt-294 |  | 11.48 | 3.72 | 8.96 | 8.09 | 7.17 | 338.13 | 103.72 | 31.84 | 679.08 | 1520.98 | 42.59 |
| TPt-216 |  | 13.22 | 4.30 | 8.77 | 8.12 | 7.16 | 390.09 | 119.75 | 32.45 | 785.45 | 1516.19 | 48.63 |
| TPt-260 |  | 12.13 | 4.13 | 9.11 | 8.12 | 7.18 | 345.44 | 112.13 | 32.41 | 693.41 | 1385.74 | 46.67 |
| TPt-187 |  | 11.86 | 3.41 | 8.34 | 7.99 | 6.99 | 387.89 | 100.11 | 30.67 | 662.78 | 1290.72 | 49.15 |
| TPt-198 |  | 11.17 | 3.51 | 8.78 | 8.28 | 7.16 | 319.25 | 102.87 | 31.19 | 630.07 | 1306.49 | 46.16 |
| TPt-215 |  | 11.47 | 3.49 | 8.41 | 8.05 | 7.20 | 344.14 | 100.96 | 31.75 | 596.62 | 1248.90 | 45.37 |
| TPt-4-A |  | 11.20 | 3.52 | 8.94 | 8.04 | 7.13 | 310.76 | 90.15 | 32.42 | 515.14 | 1198.79 | 45.16 |
| TPt-180 |  | 12.01 | 3.73 | 9.11 | 8.08 | 7.00 | 335.97 | 98.78 | 31.43 | 608.25 | 1300.81 | 47.54 |
| TPt-206 |  | 10.95 | 3.08 | 8.74 | 7.88 | 6.85 | 326.45 | 89.83 | 28.77 | 571.96 | 1294.18 | 42.15 |
| TPt-26 |  | 11.04 | 3.87 | 8.71 | 8.13 | 6.94 | 290.51 | 98.05 | 35.13 | 543.28 | 1084.85 | 49.32 |
| TPt-280 |  | 12.09 | 4.04 | 9.46 | 8.23 | 6.72 | 277.79 | 90.55 | 33.60 | 495.76 | 1120.11 | 44.84 |
| TPt-205 |  | 10.32 | 3.38 | 8.73 | 8.47 | 7.13 | 235.67 | 78.91 | 34.06 | 497.29 | 1144.38 | 43.54 |
| TPt-33 |  | 11.37 | 3.27 | 8.63 | 8.13 | 6.78 | 317.66 | 88.09 | 30.98 | 508.56 | 1113.24 | 42.83 |
| TPt-286 |  | 11.47 | 3.89 | 8.70 | 8.33 | 7.50 | 298.83 | 101.23 | 32.62 | 681.34 | 1349.30 | 50.06 |
| TPt-291 |  | 11.89 | 3.93 | 9.31 | 8.20 | 7.00 | 267.65 | 85.25 | 32.35 | 512.60 | 1153.81 | 43.11 |
| TPt-223 |  | 11.04 | 3.23 | 8.63 | 8.14 | 7.11 | 309.89 | 101.75 | 32.56 | 609.67 | 1167.73 | 47.62 |
| TPt-292 |  | 11.16 | 3.53 | 9.02 | 8.31 | 6.93 | 287.02 | 86.89 | 32.06 | 542.57 | 1263.73 | 43.37 |
| TPt-178 |  | 11.36 | 3.83 | 9.09 | 8.22 | 7.10 | 282.95 | 94.19 | 34.27 | 599.56 | 1237.39 | 48.02 |
| TPt-288 |  | 10.48 | 3.33 | 9.33 | 8.17 | 6.85 | 232.89 | 80.86 | 34.92 | 469.95 | 1085.66 | 42.36 |
| **MIN** |  | 9.44 | 2.53 | 8.06 | 7.54 | 6.38 | 175.59 | 51.08 | 24.60 | 252.01 | 568.17 | 39.06 |
| **MAX** |  | 13.42 | 4.30 | 9.46 | 8.47 | 7.50 | 390.09 | 119.75 | 35.21 | 785.45 | 1520.98 | 50.25 |
| **MEAN** |  | 11.07 | 3.33 | 8.68 | 8.03 | 6.96 | 269.25 | 80.38 | 30.59 | 489.26 | 1044.05 | 45.21 |
| **SEM** |  | 0.07 | 0.03 | 0.03 | 0.02 | 0.02 | 3.72 | 1.17 | 0.20 | 8.53 | 16.26 | 0.22 |
| **STDEV** |  | 0.71 | 0.35 | 0.27 | 0.18 | 0.20 | 38.97 | 12.28 | 2.13 | 89.44 | 170.53 | 2.36 |
| **CV(%)** |  | 6.39 | 10.38 | 3.07 | 2.22 | 2.89 | 14.47 | 15.27 | 6.96 | 18.28 | 16.33 | 5.22 |

*Note: Seed Weight Per Pod (SWPPD); Seed Length (SL); Seed Width (SWT); Seed Thickness (ST); Number of Seeds Per Plant (NSPP); Seed Weight Per Plant (SWPP); Hundred Seed Weight (HSW); Total Seed Weight (TSW); Total Pod weight (TPDW); Shelling percentage (SHP)*

**Supplementary Table S6: Mean performance based on the Duncan Multiple Range Test of agro-morphological traits of the top 20 winged bean accession based on total pod weight.**

| **Accessions** | **FPF** | **FPP** | **FPMP** | **FPDP** | **NPPP** | **PWPP** | **PW** |
| --- | --- | --- | --- | --- | --- | --- | --- |
| TPt-178 | 81.4d-l | 87.7d-w | 121.4c-j | 133.2f-x | 30.1b-t | 195.0a-l | 7.3b-q |
| TPt-180 | 82.9c-i | 89.4c-m | 122.4b-h | 135.6c-v | 36.6a-g | 213.0a-f | 7.2d-s |
| TPt-187 | 72.3r-E | 82.2q-E | 111.4p-t | 130.6q-z | 38.3a-c | 218.3a-d | 7.2b-r |
| TPt-198 | 67.6D-G | 79.4A-F | 115.0h-s | 129.2u-z | 34.4a-m | 215.3a-e | 6.7l-z |
| TPt-206 | 73.5n-E | 83.7j-C | 115.9g-s | 131.9i-x | 38.4a-c | 209.0a-g | 6.5o-B |
| TPt-215 | 73.5n-E | 84.3i-C | 118.5f-q | 136.4c-r | 35.9a-i | 213.9a-f | 7.5a-o |
| TPt-216 | 70.1v-F | 83.3k-C | 117.8g-q | 134.4d-w | 38.7a-b | 231.7a-b | 8.3a |
| TPt-220 | 75.4j-A | 82.9l-D | 115.8g-s | 133.6e-w | 29.8c-t | 179.5a-p | 6.9e-u |
| TPt-238 | 72.4r-E | 82.8m-D | 112.9l-s | 129.5t-z | 32.0a-q | 186.5a-m | 6.6n-A |
| TPt-255 | 72.7p-E | 88.3d-u | 118.1g-q | 134.8d-w | 31.3a-t | 186.1a-n | 6.8j-x |
| TPt-260 | 68.3B-G | 79.1B-F | 115.7g-s | 131.4n-y | 38.1a-d | 228.0a-c | 8.2a-c |
| TPt-270 | 74.6m-C | 82.4p-E | 117.9g-q | 132.4h-x | 35.6a-k | 233.0a | 7.9a-f |
| TPt-286 | 65.7F-G | 79.4B-F | 115.6g-s | 135.3c-w | 31.4a-t | 200.9a-j | 7.9a-g |
| TPt-29 | 76.8i-v | 85.4f-B | 115.7g-s | 130.8p-z | 33.4a-o | 186.0a-n | 6.8h-w |
| TPt-292 | 73.1o-E | 83.2k-C | 115.2h-s | 131.8i-x | 33.7a-n | 197.6a-k | 7.5a-o |
| TPt-293 | 72.4r-E | 80.5x-E | 112.9l-s | 129.1v-z | 32.1a-q | 183.8a-o | 7.0e-u |
| TPt-294 | 69.6x-F | 83.2k-C | 120.2d-n | 134.9c-w | 36.5a-h | 233.0a | 7.8a-i |
| TPt-4-A | 70.6u-F | 81.4v-E | 118.6e-q | 129.1w-z | 35.0a-l | 213.8a-f | 7.1e-t |
| TPt-6 | 72.9p-E | 82.9l-D | 119.4e-o | 131.6l-x | 31.9a-q | 183.8a-o | 7.1e-t |
| TPt-6-A | 76.6i-v | 87.7d-w | 121.1c-k | 135.3c-w | 29.5d-t | 191.7a-m | 7.8a-j |

*Note: Fifty Percent Flower (FPF); Fifty Percent Pod emergence (FPP); Fifty Percent Mature Pod (FPMP); Fifty Percent Dry Pod (FPDP); Number of Pod Per Plant (NPPP); Pod weight per plant (PWPP)*

**Supplementary Table S6 cont’d: Mean performance based on the Duncan Multiple Range Test of agro-morphological traits of the top 20 winged bean accession based on total pod weight.**

| **Accessions** | **PL** | **PWT** | **PT** | **NSPPD** | **SWPPD** | **SL** | **SWT** |
| --- | --- | --- | --- | --- | --- | --- | --- |
| TPt-178 | 20.6e-q | 10.9a-l | 9.3e-s | 11.4c-u | 3.8a-i | 9.1a-g | 8.2a-i |
| TPt-180 | 22.1b-e | 10.5c-o | 9.8a-k | 12.0b-f | 3.7b-l | 9.1a-f | 8.1b-t |
| TPt-187 | 19.8l-B | 10.5c-o | 9.2h-s | 11.9b-l | 3.4e-x | 8.3s-A | 8.0c-y |
| TPt-198 | 18.9s-N | 10.1i-o | 9.7a-o | 11.2d-w | 3.5c-u | 8.8f-u | 8.3a-g |
| TPt-206 | 18.7u-O | 10.8a-o | 9.9a-k | 10.9d-x | 3.1o-C | 8.7f-w | 7.9i-A |
| TPt-215 | 19.4o-J | 10.4d-o | 9.8a-l | 11.5bc-q | 3.5d-v | 8.4o-A | 8.1b-w |
| TPt-216 | 21.9b-g | 10.4d-o | 9.7a-p | 13.2a | 4.3a | 8.8f-u | 8.1b-q |
| TPt-220 | 19.9l-A | 10.8a-o | 9.4c-s | 11.1d-x | 3.4e-x | 8.5l-A | 8.1b-u |
| TPt-238 | 19.1q-M | 10.5c-o | 9.2f-s | 11.4c-r | 3.3i-z | 8.7f-w | 7.8m-B |
| TPt-255 | 18.2B-Q | 10.4d-o | 9.7a-o | 10.7j-z | 3.4e-x | 8.8f-u | 8.1a-n |
| TPt-260 | 21.9bc-g | 10.9a-l | 9.1h-s | 12.1b-d | 4.1a-b | 9.1a-f | 8.1b-q |
| TPt-270 | 21.7b-h | 11.0a-j | 9.4b-s | 12.0b-g | 3.9a-g | 8.9d-o | 8.1a-o |
| TPt-286 | 20.4g-t | 10.7a-o | 9.7a-o | 11.5b-q | 3.9a-h | 8.7f-w | 8.3a-c |
| TPt-29 | 19.7m-E | 10.8a-o | 9.7a-o | 10.6m-z | 3.4e-x | 8.7f-w | 8.1a-o |
| TPt-292 | 21.1d-n | 10.8a-o | 9.4b-s | 11.2d-w | 3.5c-s | 9.0b-h | 8.3a-e |
| TPt-293 | 20.3h-w | 10.6a-o | 9.6a-q | 11.2d-w | 3.5d-v | 8.6h-y | 8.0c-y |
| TPt-294 | 21.5b-k | 11.4a | 9.2f-s | 11.5b-q | 3.7b-l | 9.0b-k | 8.1b-t |
| TPt-4-A | 19.4o-I | 10.6a-o | 9.9a-k | 11.2d-w | 3.5c-t | 8.9b-l | 8.0b-x |
| TPt-6 | 20.9e-p | 10.1i-o | 9.3c-s | 11.6b-o | 3.3g-z | 8.7f-w | 7.9j-B |
| TPt-6-A | 20.5f-s | 11.0a-k | 9.5b-s | 12.0b-g | 3.7b-o | 8.8f-t | 8.1b-q |

*Note: Pod Weight (PW); Pod Length (PL); Pod Width (PWT); Number of seeds Per Pod (NSPPD); Seed Weight Per Pod (SWPPD); Seed Length (SL); Seed Width (SWT)*

**Supplementary Table S6 cont’d: Mean performance based on the Duncan Multiple Range Test of agro-morphological traits of the top 20 winged bean accession based on total pod weight.**

| **Accessions** | **ST** | **NSPP** | **SWPP** | **HSW** | **TSW** | **TPDW** | **SHP** |
| --- | --- | --- | --- | --- | --- | --- | --- |
| TPt-178 | 7.1b-q | 303.9b-m | 94.2b-m | 34.3a-e | 599.6b-i | 1237.4a-k | 48.0a-i |
| TPt-180 | 7.0b-t | 334.5a-f | 98.8a-j | 31.4a-p | 608.3b-h | 1300.8a-f | 47.5a-l |
| TPt-187 | 7.0c-v | 319.2a-i | 100.1a-i | 30.7a-r | 662.8a-e | 1290.7a-h | 49.2a-e |
| TPt-198 | 7.2a-i | 336.0a-e | 102.9a-e | 31.2a-q | 630.1b-f | 1306.5a-f | 46.2a-r |
| TPt-206 | 6.8e-x | 326.5a-g | 89.8b-q | 28.8j-s | 572.0c-o | 1294.2a-g | 42.2o-y |
| TPt-215 | 7.2a-f | 310.0b-l | 101.0a-h | 31.8a-o | 596.6b-j | 1248.9a-j | 45.4b-v |
| TPt-216 | 7.2a-i | 387.9a | 119.7a | 32.5a-l | 785.5a | 1516.2a-b | 48.6a-f |
| TPt-220 | 7.2a-i | 280.6b-r | 84.2d-v | 29.7d-r | 564.0c-o | 1202.1b-m | 46.2a-r |
| TPt-238 | 6.8e-x | 298.8b-o | 87.5c-t | 28.9i-s | 578.6b-n | 1217.6a-l | 44.5e-x |
| TPt-255 | 7.2a-g | 289.0b-p | 91.9b-p | 32.1a-m | 603.5b-h | 1209.1a-m | 45.8a-t |
| TPt-260 | 7.2a-g | 344.1a-c | 112.1a-c | 32.4a-m | 693.4a-c | 1385.7a-d | 46.7a-p |
| TPt-270 | 7.0b-t | 345.4a-b | 113.9a-b | 34.5a-d | 735.8a-b | 1507.9a-c | 46.8a-o |
| TPt-286 | 7.5a | 338.1a-d | 101.2a-g | 32.6a-l | 681.3a-d | 1349.3a-e | 50.1a-b |
| TPt-29 | 7.2a-i | 291.7b-p | 93.9b-n | 34.3a-e | 579.8b-n | 1211.6a-l | 47.1a-m |
| TPt-292 | 6.9d-w | 310.8b-k | 86.9d-u | 32.1a-m | 542.6c-p | 1263.7a-i | 43.4h-y |
| TPt-293 | 6.8e-x | 283.0b-q | 86.9d-u | 29.3f-r | 576.5b-o | 1203.9b-m | 46.7a-p |
| TPt-294 | 7.2a-i | 390.1a | 103.7a-d | 31.8a-o | 679.1a-d | 1521.0a | 42.6m-y |
| TPt-4-A | 7.1a-l | 276.0b-s | 90.1b-q | 32.4a-m | 515.1d-s | 1198.8b-m | 45.2c-v |
| TPt-6 | 6.8f-x | 287.0b-q | 87.4c-t | 29.2g-r | 589.0b-k | 1204.3b-m | 45.9a-t |
| TPt-6-A | 6.9d-w | 306.5b-m | 82.3d-v | 30.0d-r | 547.0c-p | 1237.9a-k | 43.8g-x |

*Note:* *Seed Thickness (ST); Number of Seeds Per Plant (NSPP); Seed Weight Per Plant (SWPP); Hundred Seed Weight (HSW); Total Seed Weight (TSW); Total Pod weight (TPDW); Shelling percentage (SHP)*

**Supplementary Table S7: Principal component scores of the 110 winged bean accessions for the first five principal components, ranked by PC1 based on accessions.**

| **Rank** | **Accession** | **PC1** | **PC2** | **PC3** | **PC4** | **PC5** |
| --- | --- | --- | --- | --- | --- | --- |
| 1 | **TPt-216** | -8.11 | -1.89 | 1.14 | 0.25 | 2.15 |
| 2 | **TPt-260** | -6.75 | -1.88 | -0.19 | -1.33 | 0.00 |
| 3 | **TPt-270** | -6.65 | -1.14 | 0.49 | -0.31 | -0.32 |
| 4 | **TPt-294** | -5.84 | -0.90 | 0.44 | 0.14 | -1.08 |
| 5 | **TPt-286** | -4.64 | -1.16 | -2.44 | 0.95 | 0.52 |
| 6 | **TPt-180** | -4.54 | 0.82 | 1.95 | 0.58 | 0.14 |
| 7 | **TPt-280** | -3.93 | 3.04 | 0.39 | -2.92 | -0.75 |
| 8 | **TPt-291** | -3.67 | 2.21 | -1.75 | -1.91 | -0.12 |
| 9 | **TPt-187** | -3.63 | -3.69 | 1.55 | 0.22 | 0.90 |
| 10 | **TPt-178** | -3.51 | 1.47 | -0.05 | -0.27 | -1.13 |
| 11 | **TPt-215** | -3.33 | -1.46 | 0.58 | 2.06 | 0.88 |
| 12 | **TPt-198** | -3.21 | -3.30 | -0.94 | 0.76 | -0.15 |
| 13 | **TPt-292** | -2.92 | -0.39 | -0.77 | -0.98 | -1.08 |
| 14 | **TPt-209** | -2.74 | -0.59 | -1.12 | -0.81 | 0.65 |
| 15 | **TPt-26** | -2.68 | -0.45 | -0.50 | 0.18 | -0.20 |
| 16 | **TPt-4-A** | -2.64 | -1.69 | -0.85 | 0.38 | -0.25 |
| 17 | **TPt-237** | -2.45 | 4.26 | -0.43 | -1.24 | 2.22 |
| 18 | **TPt-29** | -2.36 | -0.95 | -0.79 | 1.05 | -1.01 |
| 19 | **TPt-6-A** | -2.21 | 1.32 | 0.54 | -0.45 | 0.65 |
| 20 | **TPt-154** | -2.04 | 0.15 | -0.97 | -0.16 | 0.06 |
| 21 | **TPt-223** | -1.93 | -0.71 | 1.51 | 1.19 | -0.72 |
| 22 | **TPt-125** | -1.90 | 1.81 | -0.56 | 0.53 | 2.32 |
| 23 | **TPt-173** | -1.86 | 0.71 | 2.57 | 1.38 | -0.18 |
| 24 | **TPt-264** | -1.84 | 1.22 | -1.62 | -2.09 | 0.80 |
| 25 | **TPt-33** | -1.73 | 0.67 | 1.78 | 1.56 | -0.80 |
| 26 | **TPt-302** | -1.70 | -0.26 | -0.41 | 0.38 | 0.13 |
| 27 | **TPt-258** | -1.68 | 2.02 | -0.96 | -1.29 | 2.25 |
| 28 | **TPt-207** | -1.67 | 2.03 | -1.76 | -2.33 | 0.03 |
| 29 | **TPt-255** | -1.58 | -0.75 | -0.33 | 1.66 | -0.57 |
| 30 | **TPt-181** | -1.58 | 5.94 | 2.75 | -0.20 | -0.69 |
| 31 | **TPt-206** | -1.54 | -2.68 | 1.23 | 1.17 | -0.50 |
| 32 | **TPt-275** | -1.44 | 1.93 | 0.06 | -1.29 | -1.56 |
| 33 | **TPt-179** | -1.39 | 3.57 | 1.72 | 1.15 | -0.05 |
| 34 | **TPt-205** | -1.37 | 1.44 | -3.20 | 1.60 | -1.65 |
| 35 | **TPt-234** | -1.35 | -1.52 | 0.42 | 0.23 | -0.89 |
| 36 | **TPt-175** | -1.28 | 2.57 | 0.70 | -2.03 | 0.51 |
| 37 | **TPt-6** | -1.24 | -1.51 | 1.64 | -1.01 | 0.92 |
| 38 | **TPt-288** | -1.22 | -0.31 | -2.03 | -2.15 | -2.40 |
| 39 | **TPt-293** | -1.15 | -2.17 | -0.08 | -0.90 | 0.61 |
| 40 | **TPt-220** | -0.96 | -0.66 | -0.35 | 0.36 | 0.29 |
| 41 | **TPt-32** | -0.93 | 1.34 | -0.59 | 3.49 | -1.36 |
| 42 | **TPt-22** | -0.81 | -1.50 | -2.83 | 0.62 | 0.04 |
| 43 | **TPt-212** | -0.80 | -1.22 | -1.97 | 1.53 | -2.02 |
| 44 | **TPt-261** | -0.74 | 0.58 | -1.40 | -1.59 | 0.67 |
| 45 | **TPt-238** | -0.64 | -2.65 | 0.97 | -0.95 | 0.41 |
| 46 | **TPt-176** | -0.34 | 4.48 | 0.55 | 0.96 | -1.65 |
| 47 | **TPt-4** | -0.32 | 0.06 | 1.72 | 0.77 | 0.83 |
| 48 | **TPt-274** | -0.28 | -1.77 | 1.59 | 0.04 | 1.25 |
| 49 | **TPt-18** | -0.09 | -1.40 | -0.13 | 0.92 | -0.06 |
| 50 | **TPt-11-4** | -0.01 | -0.98 | -2.16 | -0.05 | 0.60 |
| 51 | **TPt-251** | 0.01 | 1.18 | 0.71 | 0.89 | -0.07 |
| 52 | **TPt-295** | 0.07 | 2.07 | -0.27 | 0.63 | -0.19 |
| 53 | **TPt-211** | 0.08 | -2.38 | 0.91 | 0.74 | -0.61 |
| 54 | **TPt-202** | 0.10 | 0.27 | 1.88 | -1.71 | -0.64 |
| 55 | **TPt-53** | 0.20 | 0.77 | -0.11 | 1.00 | -0.14 |
| 56 | **TPt-2** | 0.31 | -2.51 | -1.46 | 2.26 | 0.99 |
| 57 | **TPt-303** | 0.33 | -0.39 | 0.37 | -0.34 | 0.33 |
| 58 | **TPt-3-B** | 0.36 | 2.05 | -1.27 | -0.05 | 0.65 |
| 59 | **TPt-1A** | 0.42 | -0.50 | -2.66 | 1.77 | 0.12 |
| 60 | **TPt-17** | 0.42 | -3.11 | 0.24 | -1.29 | 1.68 |
| 61 | **TPt-245** | 0.48 | -2.40 | 1.67 | 0.23 | 0.91 |
| 62 | **TPt-2M** | 0.49 | -2.29 | 2.46 | 0.45 | -0.64 |
| 63 | **TPt-213** | 0.50 | -3.12 | 2.12 | -1.66 | 0.81 |
| 64 | **TPt-11** | 0.70 | 0.85 | -2.54 | -0.68 | 0.47 |
| 65 | **TPt-2A** | 0.72 | -0.07 | -0.22 | 1.08 | -1.17 |
| 66 | **TPt-12** | 0.72 | 0.20 | -1.79 | 1.04 | -0.05 |
| 67 | **TPt-267** | 0.74 | 0.55 | -0.23 | -2.72 | -1.60 |
| 68 | **TPt-51** | 0.76 | 3.71 | -1.72 | 0.30 | -0.80 |
| 69 | **TPt-16** | 0.79 | -1.41 | 0.94 | 1.12 | 0.85 |
| 70 | **TPt-284** | 0.99 | 1.16 | -1.55 | 1.40 | 0.80 |
| 71 | **TPt-290** | 1.05 | -1.08 | -0.42 | 0.54 | 1.06 |
| 72 | **TPt-10** | 1.07 | -0.54 | -1.75 | 0.36 | 0.30 |
| 73 | **TPt-43** | 1.11 | 2.18 | -1.84 | 0.44 | -0.31 |
| 74 | **TPt-19** | 1.17 | 1.44 | -1.24 | 0.88 | 0.20 |
| 75 | **TPt-272** | 1.27 | 1.43 | 3.56 | 0.25 | -0.97 |
| 76 | **TPt-15** | 1.30 | 3.00 | 1.56 | 0.20 | -0.51 |
| 77 | **TPt-248** | 1.34 | -0.04 | -0.82 | -0.18 | -1.58 |
| 78 | **TPt-230** | 1.36 | 1.12 | -0.66 | -0.98 | -0.56 |
| 79 | **TPt-177** | 1.45 | 3.15 | 0.88 | 0.56 | 1.25 |
| 80 | **TPt-9** | 1.46 | -0.41 | -0.55 | 0.97 | -0.23 |
| 81 | **TPt-268** | 1.56 | -3.36 | 0.70 | -0.82 | -2.44 |
| 82 | **TPt-266** | 1.60 | -0.39 | 1.84 | 1.38 | 0.73 |
| 83 | **TPt-250** | 1.62 | -0.45 | -1.45 | -1.46 | -1.27 |
| 84 | **TPt-48** | 1.77 | 1.63 | -0.05 | -0.42 | 0.45 |
| 85 | **TPt-214** | 1.82 | -2.04 | 2.34 | -0.14 | 2.07 |
| 86 | **TPt-225** | 1.89 | -2.02 | 0.96 | -0.36 | 1.98 |
| 87 | **TPt-257** | 2.01 | -3.46 | 1.43 | -1.16 | -1.81 |
| 88 | **TPt-287** | 2.04 | -3.82 | 0.28 | -0.97 | -0.82 |
| 89 | **TPt-252** | 2.13 | -0.30 | -1.43 | 0.70 | 2.09 |
| 90 | **TPt-164** | 2.18 | -0.47 | 0.98 | -1.60 | 2.29 |
| 91 | **TPt-276** | 2.20 | -2.21 | -0.74 | -0.62 | -0.72 |
| 92 | **TPt-3** | 2.22 | 4.34 | 0.61 | 0.09 | 1.81 |
| 93 | **TPt-14** | 2.35 | 1.07 | -1.08 | 0.25 | -0.63 |
| 94 | **TPt-153** | 2.37 | 2.18 | -0.98 | -0.34 | -0.06 |
| 95 | **TPt-126** | 2.39 | 1.66 | 0.76 | 0.71 | -0.37 |
| 96 | **TPt-174** | 3.04 | 3.31 | 3.61 | -0.94 | -0.15 |
| 97 | **TPt-30** | 3.05 | 0.21 | 1.10 | 0.20 | -1.43 |
| 98 | **TPt-31** | 3.07 | 0.33 | 0.35 | 0.82 | -0.39 |
| 99 | **TPt-42** | 3.11 | 1.31 | -0.81 | 1.20 | -1.44 |
| 100 | **TPt-182** | 3.32 | 5.50 | 2.24 | -0.26 | -0.40 |
| 101 | **TPt-279** | 3.40 | -1.06 | 3.18 | 0.06 | 0.22 |
| 102 | **TPt-203** | 3.44 | 1.19 | -0.44 | -1.58 | 0.36 |
| 103 | **TPt-289** | 3.65 | -3.37 | 2.93 | -0.45 | -1.94 |
| 104 | **TPt-283** | 4.10 | -2.60 | -0.42 | -1.20 | -1.25 |
| 105 | **TPt-5** | 4.19 | 2.69 | 1.02 | 0.50 | 1.07 |
| 106 | **TPt-172** | 4.25 | -2.84 | -3.66 | -2.92 | -0.23 |
| 107 | **TPt-169** | 4.50 | -3.05 | 1.84 | -0.29 | -0.32 |
| 108 | **TPt-16W** | 4.55 | 0.01 | -2.63 | 2.25 | 1.27 |
| 109 | **TPt-171** | 6.75 | -3.10 | -1.74 | -0.37 | 1.52 |
| 110 | **TPt-21** | 6.95 | 0.17 | -1.98 | 0.36 | 0.84 |
| **Eigenvalue** | | **7.02** | **4.52** | **2.39** | **1.40** | **1.16** |
| **Standard deviation** | | **2.65** | **2.12** | **1.55** | **1.18** | **1.08** |
| **Proportion of Variance** | | **0.35** | **0.23** | **0.12** | **0.07** | **0.06** |
| **Cumulative Proportion** | | **0.35** | **0.58** | **0.70** | **0.77** | **0.82** |
| **Variability %** | | **35.12** | **22.58** | **11.97** | **7.00** | **5.81** |

*Scores were obtained from a correlation-matrix principal component analysis of accession means for 20 agronomic and yield traits across six environments. Component signs follow those of Table 9; accordingly, negative PC1 scores identify accessions with superior pod and seed yield, whereas positive PC2 scores identify late-flowering and late-maturing accessions. Accessions are ranked in ascending order of PC1, so that the highest-yielding accessions appear first.*

## **Supplementary Table S8: Grouping of accessions in different clusters based on flowering, pod and seed yield.**

| **ACC** | **FPF** | **FPMP** | **PWPP** | **SWPP** | **ACC** | **FPF** | **FPMP** | **PWPP** | **SWPP** | **ACC** | **FPF** | **FPMP** | **PWPP** | **SWPP** |
| --- | --- | --- | --- | --- | --- | --- | --- | --- | --- | --- | --- | --- | --- | --- |
| **CLUSTER 1** | | | | | **CLUSTER 2** | | | | | **CLUSTER 3** | | | | |
| TPt-19 | 79.61 | 116.41 | 150.86 | 70.58 | TPt-180 | 82.88 | 122.38 | 212.96 | 98.78 | TPt-270 | 74.57 | 117.87 | 233.05 | 113.94 |
| TPt-48 | 76.33 | 119.67 | 149.23 | 67.52 | TPt-280 | 79.59 | 121.18 | 201.79 | 90.55 | TPt-294 | 69.56 | 120.19 | 233.01 | 103.72 |
| TPt-284 | 73.94 | 119.35 | 148.62 | 71.98 | TPt-33 | 81.11 | 121.06 | 201.12 | 88.09 | TPt-216 | 70.13 | 117.75 | 231.66 | 119.75 |
| TPt-31 | 72.75 | 119.67 | 148.42 | 71.33 | TPt-291 | 74.27 | 116.75 | 198.26 | 85.25 | TPt-260 | 68.33 | 115.67 | 227.98 | 112.13 |
| TPt-51 | 80.53 | 121.25 | 148.19 | 61.53 | TPt-292 | 73.06 | 115.24 | 197.61 | 86.89 | TPt-187 | 72.28 | 111.41 | 218.34 | 100.11 |
| TPt-250 | 72.28 | 112.72 | 147.94 | 76.60 | TPt-178 | 81.35 | 121.38 | 195.01 | 94.19 | TPt-198 | 67.56 | 115.00 | 215.33 | 102.87 |
| TPt-290 | 70.39 | 117.00 | 147.89 | 81.05 | TPt-288 | 70.27 | 112.50 | 194.29 | 80.86 | TPt-215 | 73.46 | 118.54 | 213.94 | 100.96 |
| TPt-30 | 76.76 | 119.25 | 146.77 | 70.54 | TPt-6-A | 76.63 | 121.06 | 191.69 | 82.32 | TPt-4-A | 70.63 | 118.63 | 213.80 | 90.15 |
| TPt-42 | 80.00 | 117.17 | 146.29 | 67.83 | TPt-4 | 81.78 | 118.13 | 189.19 | 80.79 | TPt-206 | 73.47 | 115.94 | 209.03 | 89.83 |
| TPt-126 | 78.88 | 122.22 | 144.13 | 68.33 | TPt-258 | 76.06 | 114.31 | 188.28 | 75.41 | TPt-26 | 72.36 | 118.93 | 202.06 | 98.05 |
| TPt-14 | 70.88 | 121.00 | 144.12 | 65.87 | TPt-237 | 81.46 | 121.14 | 187.67 | 76.24 | TPt-286 | 65.71 | 115.59 | 200.87 | 101.23 |
| TPt-203 | 75.88 | 116.69 | 141.80 | 55.93 | TPt-173 | 84.41 | 122.00 | 187.31 | 93.38 | TPt-223 | 77.13 | 120.81 | 197.87 | 101.75 |
| TPt-153 | 76.06 | 118.47 | 138.22 | 62.92 | TPt-179 | 87.64 | 126.29 | 184.92 | 84.12 | TPt-302 | 73.86 | 117.57 | 192.45 | 88.86 |
| TPt-172 | 63.47 | 105.25 | 131.50 | 63.57 | TPt-272 | 86.15 | 123.23 | 183.31 | 73.76 | TPt-211 | 73.83 | 117.19 | 192.44 | 82.66 |
| TPt-5 | 84.07 | 121.57 | 127.62 | 59.47 | TPt-202 | 77.44 | 119.94 | 183.19 | 77.00 | TPt-2M | 74.88 | 118.25 | 190.64 | 86.90 |
| TPt-16W | 69.00 | 117.00 | 124.61 | 60.81 | TPt-125 | 75.80 | 117.31 | 180.86 | 84.30 | TPt-238 | 72.44 | 112.94 | 186.48 | 87.48 |
| TPt-21 | 75.59 | 113.35 | 113.52 | 51.08 | TPt-275 | 80.64 | 117.21 | 179.30 | 78.86 | TPt-209 | 72.67 | 115.75 | 186.08 | 91.19 |
| TPt-2A | 74.88 | 118.53 | 167.05 | 78.02 | TPt-181 | 90.80 | 130.82 | 178.68 | 76.55 | TPt-255 | 72.73 | 118.13 | 186.08 | 91.88 |
| TPt-1A | 69.13 | 117.22 | 166.46 | 80.57 | TPt-207 | 74.61 | 116.67 | 176.10 | 73.41 | TPt-29 | 76.76 | 115.69 | 185.97 | 93.94 |
| TPt-266 | 77.14 | 120.33 | 166.09 | 77.39 | TPt-295 | 77.81 | 121.44 | 174.88 | 74.95 | TPt-234 | 72.56 | 116.88 | 185.64 | 95.71 |
| TPt-9 | 79.11 | 115.11 | 165.14 | 77.08 | TPt-175 | 83.71 | 117.23 | 173.81 | 75.70 | TPt-154 | 78.41 | 113.69 | 184.58 | 90.12 |
| TPt-11-4 | 71.19 | 112.38 | 164.95 | 76.49 | TPt-303 | 75.71 | 116.12 | 172.76 | 79.29 | TPt-22 | 69.71 | 112.65 | 183.93 | 89.78 |
| TPt-3-B | 76.76 | 123.19 | 162.09 | 74.31 | TPt-264 | 71.33 | 117.83 | 171.23 | 81.32 | TPt-6 | 72.93 | 119.44 | 183.84 | 87.44 |
| TPt-43 | 75.00 | 120.00 | 161.44 | 68.40 | TPt-261 | 74.38 | 114.94 | 168.85 | 81.94 | TPt-293 | 72.35 | 112.88 | 183.78 | 86.89 |
| TPt-11 | 73.67 | 115.00 | 160.04 | 75.36 | TPt-176 | 84.71 | 129.25 | 164.30 | 74.23 | TPt-245 | 73.82 | 116.56 | 182.56 | 87.65 |
| TPt-169 | 69.88 | 116.29 | 157.86 | 69.70 | TPt-251 | 81.33 | 121.24 | 163.23 | 79.68 | TPt-257 | 71.71 | 112.71 | 180.89 | 78.89 |
| TPt-10 | 72.65 | 112.17 | 157.76 | 79.40 | TPt-3 | 79.20 | 127.19 | 152.83 | 64.18 | TPt-220 | 75.40 | 115.75 | 179.49 | 84.21 |
| TPt-279 | 76.11 | 119.76 | 155.41 | 72.40 | TPt-15 | 81.73 | 128.06 | 152.13 | 70.86 | TPt-274 | 74.41 | 115.65 | 178.40 | 85.43 |
| TPt-230 | 74.13 | 117.33 | 153.87 | 66.64 | TPt-177 | 85.94 | 121.50 | 148.24 | 72.34 | TPt-213 | 73.78 | 112.82 | 176.61 | 87.56 |
| TPt-252 | 73.38 | 115.75 | 153.71 | 71.53 | TPt-174 | 86.43 | 126.06 | 141.98 | 62.38 | TPt-212 | 71.29 | 115.00 | 175.43 | 83.65 |
| TPt-248 | 73.00 | 118.94 | 153.19 | 76.42 | TPt-182 | 90.31 | 128.21 | 129.59 | 62.56 | TPt-18 | 74.60 | 113.00 | 173.80 | 85.51 |
| TPt-164 | 74.94 | 114.41 | 152.90 | 68.38 |  |  |  |  |  | TPt-268 | 68.00 | 113.69 | 172.17 | 82.29 |
| TPt-225 | 72.88 | 114.00 | 151.23 | 81.54 |  |  |  |  |  | TPt-2 | 68.06 | 114.35 | 170.45 | 82.53 |
| TPt-32 | 78.65 | 122.06 | 179.89 | 81.55 |  |  |  |  |  | TPt-17 | 71.53 | 109.00 | 170.26 | 85.43 |
| TPt-53 | 77.56 | 119.82 | 177.42 | 85.45 |  |  |  |  |  | TPt-171 | 67.50 | 113.67 | 126.42 | 62.47 |
| TPt-12 | 74.41 | 115.12 | 175.59 | 73.08 |  |  |  |  |  |  |  |  |  |  |
| TPt-289 | 70.39 | 116.53 | 173.41 | 79.74 |  |  |  |  |  |  |  |  |  |  |
| TPt-214 | 76.76 | 115.35 | 172.98 | 76.87 |  |  |  |  |  |  |  |  |  |  |
| TPt-267 | 72.76 | 116.82 | 171.88 | 69.24 |  |  |  |  |  |  |  |  |  |  |
| TPt-205 | 71.67 | 117.61 | 201.69 | 78.91 |  |  |  |  |  |  |  |  |  |  |
| TPt-16 | 72.33 | 115.35 | 186.68 | 82.28 |  |  |  |  |  |  |  |  |  |  |
| TPt-287 | 67.44 | 111.29 | 158.21 | 80.34 |  |  |  |  |  |  |  |  |  |  |
| TPt-276 | 70.72 | 112.24 | 155.77 | 74.38 |  |  |  |  |  |  |  |  |  |  |
| TPt-283 | 69.18 | 109.53 | 149.44 | 66.35 |  |  |  |  |  |  |  |  |  |  |
| **Mean** | **74.14** | **116.82** | **155.72** | **72.02** | **Mean** | **80.28** | **120.89** | **178.24** | **79.36** | **Mean** | **72.24** | **115.70** | **192.15** | **91.80** |

**Supplementary Table S9: Phenotypic correlation coefficients among 21 agronomic and yield-related traits of 110 winged bean accessions evaluated across six environments in Nigeria.**

| **Trait** | **FPF** | **FPP** | **FPMP** | **FPDP** | **NPPP** | **PWPP** | **PW** | **PL** | **PWT** | **PT** | **NSPPD** | **SWPPD** | **SL** | **SWT** | **ST** | **NSPP** | **SWPP** | **HSW** | **TSW** | **TPDW** |
| --- | --- | --- | --- | --- | --- | --- | --- | --- | --- | --- | --- | --- | --- | --- | --- | --- | --- | --- | --- | --- |
| **FPP** | **.92***** |  |  |  |  |  |  |  |  |  |  |  |  |  |  |  |  |  |  |  |
| **FPMP** | **.74***** | **.76***** |  |  |  |  |  |  |  |  |  |  |  |  |  |  |  |  |  |  |
| **FPDP** | **.63***** | **.67***** | **.82***** |  |  |  |  |  |  |  |  |  |  |  |  |  |  |  |  |  |
| **NPPP** | **-.19*** | **-.18** | **-.11** | **-.26**** |  |  |  |  |  |  |  |  |  |  |  |  |  |  |  |  |
| **PWPP** | **-.12** | **-.10** | **-.02** | **-.17** | **.75***** |  |  |  |  |  |  |  |  |  |  |  |  |  |  |  |
| **PW** | **.18** | **.18** | **.22*** | **.17** | **.06** | **.54***** |  |  |  |  |  |  |  |  |  |  |  |  |  |  |
| **PL** | **.39***** | **.34***** | **.35***** | **.23*** | **.04** | **.45***** | **.82***** |  |  |  |  |  |  |  |  |  |  |  |  |  |
| **PWT** | **.16** | **.19** | **.23*** | **.22*** | **-.05** | **.20*** | **.45***** | **.36***** |  |  |  |  |  |  |  |  |  |  |  |  |
| **PT** | **-.10** | **-.03** | **-.06** | **-.00** | **.00** | **.06** | **.20*** | **-.14** | **.23*** |  |  |  |  |  |  |  |  |  |  |  |
| **NSPPD** | **.20*** | **.19*** | **.15** | **.12** | **.08** | **.44***** | **.70***** | **.56***** | **.10** | **.14** |  |  |  |  |  |  |  |  |  |  |
| **SWPPD** | **.16** | **.17** | **.21*** | **.13** | **.12** | **.56***** | **.85***** | **.71***** | **.29**** | **.20*** | **.75***** |  |  |  |  |  |  |  |  |  |
| **SL** | **.15** | **.10** | **.19*** | **.08** | **.07** | **.38***** | **.54***** | **.62***** | **.31***** | **-.02** | **.25**** | **.57***** |  |  |  |  |  |  |  |  |
| **SWT** | **.07** | **.09** | **.19** | **.21*** | **-.02** | **.32***** | **.56***** | **.40***** | **.41***** | **.34***** | **.16** | **.56***** | **.58***** |  |  |  |  |  |  |  |
| **ST** | **-.17** | **-.10** | **-.01** | **.08** | **-.01** | **.20*** | **.35***** | **.07** | **.27**** | **.43***** | **.18** | **.36***** | **.05** | **.59***** |  |  |  |  |  |  |
| **NSPP** | **-.18** | **-.15** | **-.14** | **-.29**** | **.85***** | **.83***** | **.21*** | **.14** | **-.13** | **-.03** | **.38***** | **.33***** | **.06** | **-.02** | **.04** |  |  |  |  |  |
| **SWPP** | **-.22*** | **-.21*** | **-.11** | **-.24*** | **.76***** | **.89***** | **.41***** | **.28**** | **-.03** | **.08** | **.42***** | **.53***** | **.29**** | **.29**** | **.27**** | **.89***** |  |  |  |  |
| **HSW** | **.04** | **.06** | **.04** | **.08** | **.09** | **.41***** | **.53***** | **.42***** | **.29**** | **.19*** | **.21*** | **.53***** | **.57***** | **.64***** | **.43***** | **.12** | **.40***** |  |  |  |
| **TSW** | **-.23*** | **-.20*** | **-.11** | **-.22*** | **.71***** | **.82***** | **.39***** | **.25**** | **-.02** | **.04** | **.40***** | **.47***** | **.24*** | **.24*** | **.22*** | **.86***** | **.93***** | **.36***** |  |  |
| **TPDW** | **-.16** | **-.14** | **-.04** | **-.19*** | **.74***** | **.90***** | **.48***** | **.39***** | **.14** | **.01** | **.35***** | **.47***** | **.33***** | **.26**** | **.16** | **.81***** | **.87***** | **.37***** | **.93***** |  |
| **SHP** | **-.24*** | **-.22*** | **-.25**** | **-.25**** | **.22*** | **.13** | **-.14** | **-.31***** | **-.34***** | **.10** | **.11** | **.13** | **-.12** | **.07** | **.28**** | **.39***** | **.45***** | **.17** | **.46***** | **.18** |

**: 0.05, **: 0.01, ***:0.001. Fifty Percent Flower (FPF); Fifty Percent Pod emergence (FPP); Fifty Percent Mature Pod (FPMP); Fifty Percent Dry Pod (FPDP); Number of Pod Per Plant (NPPP); Pod weight per plant (PWPP); Pod Weight (PW); Pod Length (PL); Pod Width (PWT); Pod Thickness (PT); Number of seeds Per Pod (NSPPD); Seed Weight Per Pod (SWPPD); Seed Length (SL); Seed Width (SWT); Seed Thickness (ST); Number of Seeds Per Plant (NSPP); Seed Weight Per Plant (SWPP); Hundred Seed weight (HSW); Total Seed Weight (TSW), Total Pod Weight (TPDW) and Shelling Percentage (SHP).*

**Supplementary Table S10: Phenotypic correlation coefficients among agronomic and yield-related traits of 110 winged bean accessions, computed separately within each of the six test environments.**

**ENV1 (Ibadan 2022)**

| **Trait** | **FPF** | **FPP** | **FPMP** | **FPDP** | **NPPP** | **PWPP** | **PW** | **PL** | **PWT** | **PT** | **NSPPD** | **SWPPD** | **SL** | **SWT** | **ST** | **NSPP** | **SWPP** | **HSW** | **TSW** | **TPDW** |
| --- | --- | --- | --- | --- | --- | --- | --- | --- | --- | --- | --- | --- | --- | --- | --- | --- | --- | --- | --- | --- |
| **FPP** | .51*** |  |  |  |  |  |  |  |  |  |  |  |  |  |  |  |  |  |  |  |
| **FPMP** | .57*** | .46*** |  |  |  |  |  |  |  |  |  |  |  |  |  |  |  |  |  |  |
| **FPDP** | .35*** | .33*** | .68*** |  |  |  |  |  |  |  |  |  |  |  |  |  |  |  |  |  |
| **NPPP** | -.11 | -.14 | -.01 | -.09 |  |  |  |  |  |  |  |  |  |  |  |  |  |  |  |  |
| **PWPP** | -.08 | -.09 | .07 | -.02 | .83*** |  |  |  |  |  |  |  |  |  |  |  |  |  |  |  |
| **PW** | .23* | .17 | .21* | .13 | .04 | .31** |  |  |  |  |  |  |  |  |  |  |  |  |  |  |
| **PL** | .45*** | .25* | .37*** | .31** | -.10 | .13 | .68*** |  |  |  |  |  |  |  |  |  |  |  |  |  |
| **PWT** | .00 | .07 | .07 | -.03 | .03 | .14 | .26** | .24* |  |  |  |  |  |  |  |  |  |  |  |  |
| **PT** | -.06 | .03 | -.01 | -.06 | .21* | .35*** | .38*** | .04 | .36*** |  |  |  |  |  |  |  |  |  |  |  |
| **NSPPD** | .17 | .08 | .28** | .13 | .19 | .38*** | .47*** | .53*** | .10 | .07 |  |  |  |  |  |  |  |  |  |  |
| **SWPPD** | .14 | -.01 | .16 | .04 | .22* | .47*** | .73*** | .55*** | .25** | .32*** | .71*** |  |  |  |  |  |  |  |  |  |
| **SL** | .11 | .04 | -.00 | .18 | .01 | .14 | .42*** | .31** | -.03 | .12 | .13 | .37*** |  |  |  |  |  |  |  |  |
| **SWT** | .10 | .02 | .04 | .10 | .12 | .24* | .48*** | .23* | .13 | .38*** | .11 | .49*** | .55*** |  |  |  |  |  |  |  |
| **ST** | .08 | .12 | .07 | .16 | .11 | .19* | .31** | .13 | .01 | .33*** | .26** | .32*** | .27** | .54*** |  |  |  |  |  |  |
| **NSPP** | -.10 | -.13 | .05 | -.05 | .93*** | .92*** | .13 | -.00 | .07 | .21* | .35*** | .34*** | .05 | .15 | .14 |  |  |  |  |  |
| **SWPP** | -.13 | -.16 | .03 | -.06 | .84*** | .91*** | .26** | .05 | .16 | .31** | .45*** | .51*** | .13 | .27** | .20* | .92*** |  |  |  |  |
| **HSW** | .05 | -.03 | -.06 | .03 | .17 | .37*** | .42*** | .28** | .16 | .24* | .27** | .58*** | .52*** | .54*** | .30** | .24* | .37*** |  |  |  |
| **TSW** | -.04 | -.02 | .09 | -.03 | .67*** | .60*** | .21* | .07 | .03 | .13 | .32** | .39*** | .11 | .30** | .15 | .71*** | .73*** | .33*** |  |  |
| **TPDW** | -.00 | .03 | .14 | .00 | .70*** | .72*** | .27** | .13 | .01 | .19* | .30** | .38*** | .12 | .29** | .13 | .75*** | .73*** | .33*** | .95*** |  |
| **SHP** | -.04 | -.12 | -.01 | -.12 | .34*** | .38*** | .03 | -.16 | -.11 | -.05 | .26** | .30** | .08 | .16 | .26** | .47*** | .47*** | .28** | .39*** | .33*** |

**ENV2 (Ikenne 2022)**

| **Trait** | **FPF** | **FPP** | **FPMP** | **FPDP** | **NPPP** | **PWPP** | **PW** | **PL** | **PWT** | **PT** | **NSPPD** | **SWPPD** | **SL** | **SWT** | **ST** | **NSPP** | **SWPP** | **HSW** | **TSW** | **TPDW** |
| --- | --- | --- | --- | --- | --- | --- | --- | --- | --- | --- | --- | --- | --- | --- | --- | --- | --- | --- | --- | --- |
| **FPP** | .87*** |  |  |  |  |  |  |  |  |  |  |  |  |  |  |  |  |  |  |  |
| **FPMP** | .65*** | .75*** |  |  |  |  |  |  |  |  |  |  |  |  |  |  |  |  |  |  |
| **FPDP** | .74*** | .80*** | .79*** |  |  |  |  |  |  |  |  |  |  |  |  |  |  |  |  |  |
| **NPPP** | -.10 | -.01 | .05 | .05 |  |  |  |  |  |  |  |  |  |  |  |  |  |  |  |  |
| **PWPP** | -.12 | -.10 | -.11 | -.12 | .73*** |  |  |  |  |  |  |  |  |  |  |  |  |  |  |  |
| **PW** | -.06 | -.10 | -.07 | -.15 | .22* | .59*** |  |  |  |  |  |  |  |  |  |  |  |  |  |  |
| **PL** | .15 | .07 | .09 | .04 | .10 | .48*** | .78*** |  |  |  |  |  |  |  |  |  |  |  |  |  |
| **PWT** | .17 | .13 | .09 | .09 | .19 | .14 | .28** | .20* |  |  |  |  |  |  |  |  |  |  |  |  |
| **PT** | -.05 | -.09 | .07 | .02 | .18 | -.02 | .08 | -.03 | .44*** |  |  |  |  |  |  |  |  |  |  |  |
| **NSPPD** | -.13 | -.17 | -.12 | -.17 | .31** | .54*** | .71*** | .58*** | .07 | -.00 |  |  |  |  |  |  |  |  |  |  |
| **SWPPD** | -.06 | -.06 | -.07 | -.10 | .27** | .61*** | .91*** | .67*** | .14 | .01 | .79*** |  |  |  |  |  |  |  |  |  |
| **SL** | .12 | .05 | .02 | -.01 | .11 | .51*** | .60*** | .60*** | .31** | .03 | .39*** | .56*** |  |  |  |  |  |  |  |  |
| **SWT** | .08 | .04 | -.03 | -.01 | .21* | .55*** | .63*** | .49*** | .27** | .01 | .33*** | .57*** | .74*** |  |  |  |  |  |  |  |
| **ST** | .02 | .03 | -.19 | .00 | .11 | .24* | .36*** | .21* | .24* | .03 | .27** | .40*** | .21* | .42*** |  |  |  |  |  |  |
| **NSPP** | -.15 | -.10 | -.08 | -.08 | .84*** | .89*** | .34*** | .24* | .07 | .00 | .50*** | .43*** | .28** | .28** | .13 |  |  |  |  |  |
| **SWPP** | -.18 | -.15 | -.14 | -.14 | .73*** | .93*** | .52*** | .35*** | .07 | .05 | .50*** | .59*** | .40*** | .46*** | .27** | .88*** |  |  |  |  |
| **HSW** | -.06 | -.12 | -.08 | -.05 | .24* | .50*** | .46*** | .31** | .10 | -.02 | .26** | .46*** | .44*** | .53*** | .26** | .28** | .47*** |  |  |  |
| **TSW** | -.15 | -.09 | -.15 | -.18 | .67*** | .63*** | .34*** | .15 | .22* | .19 | .41*** | .39*** | .18 | .19* | .26** | .69*** | .75*** | .21* |  |  |
| **TPDW** | -.12 | -.09 | -.18 | -.21* | .66*** | .62*** | .42*** | .25** | .31** | .20* | .37*** | .42*** | .21* | .31** | .39*** | .62*** | .70*** | .22* | .93*** |  |
| **SHP** | -.19 | -.11 | -.03 | -.01 | .21* | .24* | -.10 | -.28** | -.23* | -.05 | .22* | .13 | -.01 | -.13 | -.09 | .43*** | .36*** | .16 | .27** | .01 |

**ENV3 (Ibadan 2023)**

| **Trait** | **FPF** | **FPP** | **FPMP** | **FPDP** | **NPPP** | **PWPP** | **PW** | **PL** | **PWT** | **PT** | **NSPPD** | **SWPPD** | **SL** | **SWT** | **ST** | **NSPP** | **SWPP** | **HSW** | **TSW** | **TPDW** |
| --- | --- | --- | --- | --- | --- | --- | --- | --- | --- | --- | --- | --- | --- | --- | --- | --- | --- | --- | --- | --- |
| **FPP** | .91*** |  |  |  |  |  |  |  |  |  |  |  |  |  |  |  |  |  |  |  |
| **FPMP** | .46*** | .49*** |  |  |  |  |  |  |  |  |  |  |  |  |  |  |  |  |  |  |
| **FPDP** | .43*** | .42*** | .68*** |  |  |  |  |  |  |  |  |  |  |  |  |  |  |  |  |  |
| **NPPP** | -.24* | -.22* | -.12 | -.08 |  |  |  |  |  |  |  |  |  |  |  |  |  |  |  |  |
| **PWPP** | -.26** | -.24* | -.10 | -.16 | .70*** |  |  |  |  |  |  |  |  |  |  |  |  |  |  |  |
| **PW** | .01 | .07 | .03 | .03 | -.16 | .16 |  |  |  |  |  |  |  |  |  |  |  |  |  |  |
| **PL** | .20* | .20* | .21* | .11 | -.27** | .18 | .72*** |  |  |  |  |  |  |  |  |  |  |  |  |  |
| **PWT** | .05 | .04 | -.02 | .13 | -.23* | .01 | .26** | .21* |  |  |  |  |  |  |  |  |  |  |  |  |
| **PT** | -.13 | -.12 | -.05 | -.08 | -.03 | .11 | .31*** | .02 | .42*** |  |  |  |  |  |  |  |  |  |  |  |
| **NSPPD** | .05 | .04 | .09 | .00 | .02 | .37*** | .55*** | .58*** | .17 | .26** |  |  |  |  |  |  |  |  |  |  |
| **SWPPD** | -.07 | -.05 | .04 | -.04 | -.09 | .36*** | .86*** | .65*** | .31** | .35*** | .81*** |  |  |  |  |  |  |  |  |  |
| **SL** | -.09 | -.14 | .07 | -.02 | -.07 | .27** | .23* | .41*** | .12 | .04 | .06 | .35*** |  |  |  |  |  |  |  |  |
| **SWT** | -.18 | -.16 | .01 | -.05 | -.27** | .11 | .31** | .33*** | .44*** | .34*** | .11 | .49*** | .68*** |  |  |  |  |  |  |  |
| **ST** | -.27** | -.22* | -.05 | -.10 | -.08 | .17 | .30** | .12 | .42*** | .48*** | .17 | .50*** | .25** | .67*** |  |  |  |  |  |  |
| **NSPP** | -.28** | -.28** | -.18 | -.20* | .81*** | .84*** | .07 | -.05 | -.17 | .05 | .38*** | .25** | -.00 | -.15 | .03 |  |  |  |  |  |
| **SWPP** | -.32*** | -.31*** | -.16 | -.21* | .67*** | .89*** | .18 | .08 | -.02 | .16 | .44*** | .45*** | .23* | .14 | .27** | .89*** |  |  |  |  |
| **HSW** | -.10 | -.05 | .07 | -.11 | -.18 | .09 | .46*** | .29** | .20* | .19 | .25** | .49*** | .34*** | .46*** | .37*** | -.05 | .16 |  |  |  |
| **TSW** | -.35*** | -.29** | -.20* | -.25** | .56*** | .73*** | .35*** | .13 | -.09 | .13 | .39*** | .47*** | .13 | .08 | .17 | .79*** | .85*** | .26** |  |  |
| **TPDW** | -.30** | -.24* | -.17 | -.19* | .60*** | .81*** | .31*** | .21* | -.08 | .04 | .28** | .35*** | .16 | .03 | .03 | .73*** | .72*** | .13 | .87*** |  |
| **SHP** | -.27** | -.27** | -.14 | -.23* | .12 | .09 | .08 | -.18 | -.11 | .20* | .18 | .25** | .01 | .10 | .28** | .39*** | .47*** | .16 | .48*** | .10 |

**ENV4 (Ikenne 2023)**

| **Trait** | **FPF** | **FPP** | **FPMP** | **FPDP** | **NPPP** | **PWPP** | **PW** | **PL** | **PWT** | **PT** | **NSPPD** | **SWPPD** | **SL** | **SWT** | **ST** | **NSPP** | **SWPP** | **HSW** | **TSW** | **TPDW** |
| --- | --- | --- | --- | --- | --- | --- | --- | --- | --- | --- | --- | --- | --- | --- | --- | --- | --- | --- | --- | --- |
| **FPP** | .95*** |  |  |  |  |  |  |  |  |  |  |  |  |  |  |  |  |  |  |  |
| **FPMP** | .65*** | .68*** |  |  |  |  |  |  |  |  |  |  |  |  |  |  |  |  |  |  |
| **FPDP** | .49*** | .53*** | .74*** |  |  |  |  |  |  |  |  |  |  |  |  |  |  |  |  |  |
| **NPPP** | -.18 | -.10 | .13 | .13 |  |  |  |  |  |  |  |  |  |  |  |  |  |  |  |  |
| **PWPP** | -.21* | -.15 | .05 | .07 | .84*** |  |  |  |  |  |  |  |  |  |  |  |  |  |  |  |
| **PW** | .13 | .09 | .06 | .08 | -.15 | .12 |  |  |  |  |  |  |  |  |  |  |  |  |  |  |
| **PL** | .25** | .22* | .10 | .09 | -.20* | .03 | .79*** |  |  |  |  |  |  |  |  |  |  |  |  |  |
| **PWT** | .05 | .06 | .16 | .08 | -.13 | -.03 | .32*** | .20* |  |  |  |  |  |  |  |  |  |  |  |  |
| **PT** | -.08 | -.08 | -.04 | .05 | .07 | .19* | .19* | -.15 | .35*** |  |  |  |  |  |  |  |  |  |  |  |
| **NSPPD** | .05 | -.02 | -.10 | .01 | -.00 | .13 | .57*** | .49*** | -.08 | -.11 |  |  |  |  |  |  |  |  |  |  |
| **SWPPD** | .03 | -.03 | -.01 | .01 | -.01 | .23* | .83*** | .60*** | .11 | .16 | .75*** |  |  |  |  |  |  |  |  |  |
| **SL** | .16 | .11 | .09 | .05 | -.22* | .01 | .50*** | .57*** | .28** | .03 | .05 | .43*** |  |  |  |  |  |  |  |  |
| **SWT** | -.10 | -.11 | -.07 | .00 | -.13 | .07 | .36*** | .18 | .31*** | .47*** | -.06 | .36*** | .61*** |  |  |  |  |  |  |  |
| **ST** | -.22* | -.23* | -.12 | .00 | .02 | .14 | .20* | -.11 | .17 | .59*** | -.06 | .28** | .18 | .71*** |  |  |  |  |  |  |
| **NSPP** | -.23* | -.20* | .00 | -.00 | .87*** | .90*** | -.07 | -.16 | -.21* | .05 | .19* | .15 | -.22* | -.15 | .03 |  |  |  |  |  |
| **SWPP** | -.24* | -.22* | -.02 | -.03 | .81*** | .93*** | .06 | -.08 | -.12 | .16 | .16 | .28** | -.04 | .07 | .19 | .93*** |  |  |  |  |
| **HSW** | .07 | .09 | .11 | .08 | .01 | .11 | .19* | .13 | .14 | .27** | -.18 | .16 | .47*** | .47*** | .33*** | -.08 | .11 |  |  |  |
| **TSW** | -.24* | -.22* | -.06 | -.06 | .78*** | .90*** | .08 | -.06 | -.14 | .16 | .20* | .30** | -.04 | .05 | .16 | .90*** | .97*** | .14 |  |  |
| **TPDW** | -.18 | -.14 | .04 | .06 | .81*** | .97*** | .13 | .06 | -.07 | .20* | .16 | .24* | .00 | .05 | .12 | .87*** | .91*** | .16 | .92*** |  |
| **SHP** | -.28** | -.34*** | -.32*** | -.33*** | .11 | .09 | -.09 | -.28** | -.27** | -.04 | .19* | .26** | -.09 | .09 | .19 | .31*** | .39*** | -.02 | .37*** | .07 |

**ENV5 (Kano 2023)**

| **Trait** | **FPF** | **FPP** | **FPMP** | **FPDP** | **NPPP** | **PWPP** | **PW** | **PL** | **PWT** | **NSPPD** | **SWPPD** | **SL** | **SWT** | **ST** | **NSPP** | **SWPP** | **HSW** | **TSW** | **TPDW** |
| --- | --- | --- | --- | --- | --- | --- | --- | --- | --- | --- | --- | --- | --- | --- | --- | --- | --- | --- | --- |
| **FPP** | .84*** |  |  |  |  |  |  |  |  |  |  |  |  |  |  |  |  |  |  |
| **FPMP** | .53*** | .66*** |  |  |  |  |  |  |  |  |  |  |  |  |  |  |  |  |  |
| **FPDP** | .28** | .34*** | .58*** |  |  |  |  |  |  |  |  |  |  |  |  |  |  |  |  |
| **NPPP** | .17 | .12 | .10 | -.05 |  |  |  |  |  |  |  |  |  |  |  |  |  |  |  |
| **PWPP** | .08 | .06 | .07 | -.00 | .44*** |  |  |  |  |  |  |  |  |  |  |  |  |  |  |
| **PW** | .12 | -.06 | -.03 | .00 | .07 | .06 |  |  |  |  |  |  |  |  |  |  |  |  |  |
| **PL** | .03 | -.05 | .02 | .09 | .08 | .10 | .17 |  |  |  |  |  |  |  |  |  |  |  |  |
| **PWT** | -.07 | -.05 | .00 | .10 | .10 | .18 | .05 | .34** |  |  |  |  |  |  |  |  |  |  |  |
| **NSPPD** | .33** | .21 | .10 | -.21 | .32** | .17 | .08 | .10 | -.16 |  |  |  |  |  |  |  |  |  |  |
| **SWPPD** | .09 | -.01 | .03 | -.19 | .17 | .23* | -.04 | .41*** | .08 | .33** |  |  |  |  |  |  |  |  |  |
| **SL** | -.14 | -.16 | -.20 | -.08 | .08 | .09 | .00 | .02 | .01 | -.06 | .17 |  |  |  |  |  |  |  |  |
| **SWT** | -.11 | -.07 | -.08 | .14 | .18 | .21* | .19 | .38*** | .05 | -.16 | .06 | .46*** |  |  |  |  |  |  |  |
| **ST** | -.06 | .04 | -.05 | .11 | .11 | .11 | .07 | .09 | -.02 | .08 | .02 | .28** | .51*** |  |  |  |  |  |  |
| **NSPP** | .04 | -.01 | -.04 | -.19 | .60*** | .49*** | .02 | .28** | .21* | .34** | .40*** | .08 | .19 | .16 |  |  |  |  |  |
| **SWPP** | -.03 | -.03 | .06 | -.07 | .59*** | .51*** | -.03 | .14 | .12 | .25* | .35*** | .12 | .22* | .17 | .84*** |  |  |  |  |
| **HSW** | -.26* | -.26** | -.27** | -.01 | -.08 | .15 | .06 | .06 | .00 | -.12 | .23* | .08 | .12 | .20 | .18 | .23* |  |  |  |
| **TSW** | -.12 | -.11 | -.18 | -.12 | .50*** | .37*** | -.01 | -.08 | .06 | .08 | .00 | .00 | .09 | .01 | .58*** | .56*** | .34*** |  |  |
| **TPDW** | -.19 | -.16 | -.17 | -.18 | .31** | .60*** | -.04 | -.03 | .10 | .02 | .05 | .12 | .09 | .07 | .41*** | .49*** | .19 | .50*** |  |
| **SHP** | .07 | -.03 | .04 | -.07 | -.12 | -.08 | -.04 | -.06 | -.16 | .10 | .12 | -.17 | -.11 | .04 | .11 | .08 | .14 | -.01 | -.03 |

**ENV6 (Mokwa 2023)**

| **Trait** | **FPF** | **FPP** | **FPMP** | **FPDP** | **NPPP** | **PWPP** | **PW** | **PL** | **PWT** | **PT** | **NSPPD** | **SWPPD** | **SL** | **SWT** | **ST** | **NSPP** | **SWPP** | **HSW** | **TSW** | **TPDW** |
| --- | --- | --- | --- | --- | --- | --- | --- | --- | --- | --- | --- | --- | --- | --- | --- | --- | --- | --- | --- | --- |
| **FPP** | .92*** |  |  |  |  |  |  |  |  |  |  |  |  |  |  |  |  |  |  |  |
| **FPMP** | .73*** | .87*** |  |  |  |  |  |  |  |  |  |  |  |  |  |  |  |  |  |  |
| **FPDP** | .63*** | .75*** | .86*** |  |  |  |  |  |  |  |  |  |  |  |  |  |  |  |  |  |
| **NPPP** | -.17 | -.18 | -.10 | -.09 |  |  |  |  |  |  |  |  |  |  |  |  |  |  |  |  |
| **PWPP** | -.14 | -.11 | -.03 | -.04 | .80*** |  |  |  |  |  |  |  |  |  |  |  |  |  |  |  |
| **PW** | .17 | .17 | .13 | .08 | .34*** | .34*** |  |  |  |  |  |  |  |  |  |  |  |  |  |  |
| **PL** | .03 | .08 | .08 | .01 | .20* | .32*** | .64*** |  |  |  |  |  |  |  |  |  |  |  |  |  |
| **PWT** | .17 | .17 | .18 | .09 | -.14 | -.11 | .21* | .03 |  |  |  |  |  |  |  |  |  |  |  |  |
| **PT** | .03 | -.04 | -.06 | -.11 | .05 | -.03 | .37*** | .06 | .14 |  |  |  |  |  |  |  |  |  |  |  |
| **NSPPD** | .17 | .23* | .21* | .15 | .31** | .37*** | .65*** | .48*** | .07 | .19* |  |  |  |  |  |  |  |  |  |  |
| **SWPPD** | .11 | .12 | .13 | .12 | .35*** | .43*** | .72*** | .43*** | .12 | .34*** | .62*** |  |  |  |  |  |  |  |  |  |
| **SL** | .12 | .12 | .10 | .01 | .14 | .27** | .40*** | .42*** | .19* | .18 | .21* | .48*** |  |  |  |  |  |  |  |  |
| **SWT** | .12 | .09 | .11 | .08 | .30** | .33*** | .52*** | .22* | .23* | .20* | .23* | .60*** | .40*** |  |  |  |  |  |  |  |
| **ST** | -.10 | -.10 | -.01 | -.04 | .31*** | .30** | .44*** | .08 | .20* | .15 | .26** | .39*** | -.02 | .52*** |  |  |  |  |  |  |
| **NSPP** | -.16 | -.12 | -.02 | -.02 | .82*** | .83*** | .28** | .18 | -.19* | -.04 | .37*** | .32*** | .07 | .24* | .29** |  |  |  |  |  |
| **SWPP** | -.17 | -.15 | -.03 | -.02 | .77*** | .82*** | .38*** | .22* | -.09 | .06 | .36*** | .44*** | .15 | .37*** | .42*** | .90*** |  |  |  |  |
| **HSW** | -.10 | -.07 | -.03 | -.09 | .05 | .22* | .29** | .27** | .23* | .18 | .15 | .43*** | .27** | .39*** | .29** | .08 | .24* |  |  |  |
| **TSW** | -.23* | -.17 | -.06 | -.01 | .38*** | .43*** | .26** | .31*** | -.04 | .06 | .33*** | .44*** | .20* | .23* | .24* | .42*** | .51*** | .29** |  |  |
| **TPDW** | -.19* | -.13 | -.06 | -.02 | .36*** | .47*** | .22* | .33*** | -.04 | .04 | .28** | .39*** | .25** | .17 | .14 | .34*** | .35*** | .25** | .91*** |  |
| **SHP** | -.09 | -.10 | -.00 | .02 | .02 | -.05 | -.05 | -.24* | -.22* | .10 | .00 | .09 | -.13 | .12 | .10 | .22* | .28** | .03 | .19* | -.04 |

**Supplementary Table S11. Sensitivity analysis of AMMI and GGE biplot results for seed weight per plant (SWPP) of 30 winged bean accessions: comparison of the full six-environment dataset with the balanced subset of four environments evaluated in 2023.**

| **Code** | **Accession** | **Six environments (ENV1–ENV6)** | | | | **2023 environments only (ENV3–ENV6)** | | | |
| --- | --- | --- | --- | --- | --- | --- | --- | --- | --- |
|  |  | **Rank** | **Mean SWPP (g)** | **IPCA 1** | **Distance to ideal** | **Rank** | **Mean SWPP (g)** | **IPCA 1** | **Distance to ideal** |
| **G31** | **TPt-198** | **1** | **53.15** | **0.89** | **2.33** | **1** | **54.34** | **-0.61** | **2.40** |
| **G70** | **TPt-270** | **2** | **51.40** | **-0.17** | **5.57** | **2** | **49.91** | **0.10** | **9.40** |
| **G29** | **TPt-187** | **3** | **50.58** | **0.06** | **6.88** | **3** | **49.80** | **0.15** | **9.55** |
| G49 | TPt-223 | 4 | 47.69 | 1.87 | 16.87 | 4 | 49.51 | -1.79 | 12.96 |
| G98 | TPt-33 | 5 | 47.39 | -0.50 | 15.36 | 7 | 47.90 | 0.55 | 13.65 |
| G88 | TPt-294 | 6 | 47.30 | 0.74 | 14.96 | 6 | 48.04 | -0.61 | 13.17 |
| G26 | TPt-180 | 7 | 47.17 | -0.04 | 15.24 | 9 | 46.57 | 0.12 | 16.12 |
| G79 | TPt-286 | 8 | 46.84 | 0.70 | 16.85 | 5 | 48.69 | -0.64 | 11.90 |
| G18 | TPt-173 | 9 | 45.92 | 1.00 | 18.68 | 8 | 47.50 | -0.84 | 14.07 |
| G76 | TPt-280 | 10 | 45.68 | 1.19 | 19.48 | 10 | 45.85 | -1.17 | 18.55 |
| G47 | TPt-22 | 11 | 45.62 | -1.16 | 20.62 | 14 | 45.13 | 1.32 | 20.24 |
| G23 | TPt-178 | 12 | 45.46 | 1.02 | 19.80 | 11 | 45.85 | -0.97 | 17.93 |
| G63 | TPt-26 | 13 | 45.29 | -1.04 | 21.38 | 12 | 45.71 | 1.43 | 19.64 |
| G86 | TPt-292 | 14 | 45.10 | 0.77 | 21.57 | 13 | 45.66 | -0.95 | 18.35 |
| G43 | TPt-213 | 15 | 44.39 | -0.46 | 22.52 | 15 | 44.20 | 0.45 | 20.98 |
| G54 | TPt-238 | 16 | 44.30 | 0.14 | 22.14 | 16 | 44.17 | -0.09 | 20.56 |
| G52 | TPt-234 | 17 | 44.28 | 1.50 | 24.44 | 18 | 43.67 | -1.79 | 24.26 |
| G12 | TPT-16 | 18 | 43.90 | 0.52 | 22.68 | 17 | 44.00 | -0.27 | 21.39 |
| G39 | TPt-209 | 19 | 43.89 | 0.95 | 23.38 | 19 | 43.65 | -0.96 | 22.16 |
| G108 | TPt-6 | 20 | 42.93 | 0.90 | 25.20 | 20 | 43.57 | -0.71 | 21.59 |
| G96 | TPt-31 | 21 | 33.40 | -1.82 | 50.42 | 21 | 32.53 | 1.71 | 45.21 |
| G22 | TPt-177 | 22 | 31.10 | -1.07 | 54.95 | 23 | 30.67 | 1.09 | 48.38 |
| G10 | TPt-153 | 23 | 31.08 | -1.02 | 55.42 | 22 | 31.40 | 0.92 | 46.89 |
| G1 | TPT 16W | 24 | 31.00 | -0.49 | 56.12 | 24 | 30.44 | -0.00 | 48.76 |
| G17 | TPt-172 | 25 | 29.89 | 0.35 | 57.48 | 26 | 29.70 | -0.42 | 49.99 |
| G106 | TPt-51 | 26 | 29.39 | -0.95 | 59.68 | 27 | 28.95 | 0.66 | 51.69 |
| G28 | TPt-182 | 27 | 28.74 | -1.83 | 61.53 | 25 | 30.03 | 1.91 | 50.17 |
| G40 | TPt-21 | 28 | 28.35 | 0.17 | 61.95 | 28 | 28.69 | -0.37 | 52.32 |
| G19 | TPt-174 | 29 | 27.41 | -0.79 | 64.25 | 29 | 27.19 | 0.58 | 55.08 |
| G35 | TPt-203 | 30 | 23.44 | -1.45 | 74.35 | 30 | 23.57 | 1.25 | 62.56 |

**Supplementary Figure S1: Classification of 21 agronomic and yield-related traits of 110 winged bean accessions by genetic advance as a percentage of the mean at 5% selection intensity.**

**Supplementary Figure S2: Broad-sense heritability estimates for 21 agronomic and yield-related traits of 110 winged bean accessions evaluated across six environments in Nigeria.**

**Supplementary Figure S3: Scree plot diagram of Principal Components of agronomic and yield traits of 110 accessions of Winged bean accessions.**

|  | Cluster 1 | Cluster 2 | Cluster 3 |
| --- | --- | --- | --- |
| FPF | 72.24 | 80.28 | 74.14 |
| FPMP | 115.701 | 120.892 | 116.823 |
| PWPP | 192.15 | 178.24 | 155.72 |
| SWPP | 91.8 | 79.36 | 72.02 |

## **Supplementary Figure S4: Bar chart showing the mean of yield and agronomic traits for each cluster in the winged bean accessions studied.**
